# Delivery of Basic Fibroblast Growth Factor Through an *In Situ* Forming Smart Hydrogel Activates Autophagy in Schwann Cells and Improves Facial Nerves Generation *via* the PAK-1 Signaling Pathway

**DOI:** 10.3389/fphar.2022.778680

**Published:** 2022-04-01

**Authors:** Binbin Hu, Hanbo Zhang, Menglu Xu, Lei Li, Man Wu, Susu Zhang, Xuejun Liu, Weidong Xia, Ke Xu, Jian Xiao, Hongyu Zhang, Liyan Ni

**Affiliations:** ^1^ Department of Otorhinolaryngology, The Second Affiliated Hospital and Yuying Children’s Hospital of Wenzhou Medical University, Wenzhou, China; ^2^ Key Laboratory of Biotechnology and Pharmaceutical Engineering, School of Pharmaceutical Sciences, Cixi Biomedical Research Institute, Wenzhou Medical University, Wenzhou, China; ^3^ Department of Burn, First Affiliated Hospital of Wenzhou Medical University, Wenzhou, China

**Keywords:** facial nerve injury, autophagy, Pak1, *in situ* forming hydrogel, basic fibroblast growth factor

## Abstract

Although studies have shown that basic fibroblast growth factor (bFGF) can activate autophagy and promote peripheral nerve repair, the role and the molecular mechanism of action of bFGF in the facial nerve are not clear. In this study, a thermosensitive *in situ* forming poloxamer hydrogel was used as a vehicle to deliver bFGF for treating facial nerve injury (FNI) in the rat model. Using H&E and Masson’s staining, we found that bFGF hydrogel can promote the functional recovery and regeneration of the facial nerve. Furthermore, studies on the mechanism showed that bFGF can promote FNI recovery by promoting autophagy and inhibiting apoptosis. Additionally, this study demonstrated that the role of hydrogel binding bFGF in nerve repair was mediated through the activation of the PAK1 signaling pathway in Schwann cells (SCs). These results indicated that poloxamer thermosensitive hydrogel loaded with bFGF can significantly restore the morphology and function of the injured facial nerve by promoting autophagy and inhibiting apoptosis by activating the PAK1 pathway, which can provide a promising strategy for FNI recovery.

## Introduction

About 20 out of 100,000 people are diagnosed with facial nerve injury (FNI) annually, and it is mostly caused by trauma and iatrogenic injury (Brown et al., 2019). Currently, even if an injured nerve can be repaired by experienced surgeons through delicate surgery, it is still difficult to completely restore the damaged nerve function (Ali et al., 2020). After facial nerve damage, the lesion area and its distal stump can undergo Wallerian degeneration (WD), including axon degeneration, demyelination and phagocytosis. The destruction of myelin sheaths during WD produces a large amount of myelin debris and seriously hinders the regeneration and functional recovery of the nerves (Colavincenzo and Levine, 2000; Zhou et al., 2019). Around 40–50% of residual myelin debris was removed by the Schwann cells (SCs) in the first five to 7 days after injury (Niemi et al., 2013; Jessen and Mirsky, 2016). Moreover, some studies reported that the cellular mechanism of impaired myelin degradation during WD was closely related to the activation of autophagy in SCs (Gomez-Sanchez et al., 2015; Wakatsuki et al., 2017; Wang et al., 2019). Autophagy is a common cellular protection process in eukaryotes. It plays a key role in maintaining the homeostasis of cells and tissues by removing damaged organelles, pathological proteins, and dysfunctional macromolecules in cells. Autophagy regulates various physiological and pathological processes through lysosomal degradation pathways, such as nerve regeneration, myelin development, myelin degradation and neuropathic pain (Marinelli et al., 2014; Gomez-Sanchez et al., 2015; Jang et al., 2015; Jang et al., 2016).

Hence, a new therapeutic strategy to repair FNI is to activate the autophagy pathway in the early stage after FNI. The basic fibroblast growth factor (bFGF) is a powerful neurotrophic factor secreted by SCs and different neuronal populations (Jungnickel et al., 2006). After peripheral nerve injury (PNI), the upregulation of the mRNA and protein of bFGF cannot maintain the survival of neurons, and the axon prolongs (Grosheva et al., 2016). The overexpression of bFGF through a lentivirus transfection in SCs promotes muscular reinnervation and stimulates the regeneration of motor and sensory neurons in the peripheral nervous system (PNS) (Allodi et al., 2014). Additionally, bFGF has several biological properties associated with nerve protection, neuronal occurrence and angiogenesis in the PNS (Beenken and Mohammadi, 2009; Anderson et al., 2018). However, bFGF has a very short half-life and is easily inactivated in body fluids by various proteases (Rapraeger et al., 1991). Thus, it is important to use a suitable drug carrier to maintain the biological activity and availability of bFGF in the lesion and to control the release of bFGF. Thermosensitive hydrogel poloxamer 407 (P 407) is a suitable carrier and a controlled-release system for protein drugs (Al Khateb et al., 2016; Gausterer et al., 2020; Yang et al., 2020). It can maintain the stability of the protein and improve its bioavailability. We found that a thermosensitive heparin-poloxamer hydrogel combined with bFGF can effectively facilitate SC proliferation, axonal regeneration and recovery of motor function in diabetic rats with PNI (Li et al., 2018). Heparin can cause coagulation dysfunction, and the synthesis of heparin-poloxamer is time-consuming and laborious. In our study, poloxamer loaded with bFGF (P-bFGF) was used, and once the thermosensitive P-bFGF was injected into the FNI lesion, it turned from liquid to hydrogel *in situ*. The hydrogel remained at the site of the lesion for a long time and maintains the biological activity and availability of bFGF. Additionally, the protein was released slowly, resulting in a lasting bFGF therapy for FNI. Additionally, previous studies have also shown that the role of bFGF in the regulation of early peripheral nerve regeneration was closely related to the SC-mediated autophagy activation to remove myelin debris (Jiang et al., 2021).

Nevertheless, the molecular mechanism underlying the relationship between bFGF-induced nerve regeneration and SC-mediated myelin phagocytosis remains to be elucidated. P21-activated kinases 1 (PAK1) is a conserved serine/threonine-protein kinase. It needs phosphorylation of threonine 423 to be fully activated and is involved in various signal cascades on the surface of and within cells. It affects the cell cycle, proliferation, apoptosis, transformation, and migration of cells, and redox, inflammation, metabolism, gene expression and other processes. It is also associated with many diseases, such as cancer, nervous system diseases and heart diseases (Diebold et al., 2004; Han et al., 2017). A study reported that P21 activated autophagy by regulating the phosphorylation of Akt and AMPK to protect against cardiac hypertrophy, which was validated in a P21-knockout mouse model (Xu et al., 2019). The expression of PAK1 in DU145 (a kind of human prostate cancer cell) was knocked out by the short hairpin RNA (shRNA) method, the expressions of p-PAK1, mTOR and Beclin1 decreased, and the ratio of LC3B2/LC3B1 (microtubule-associated protein 1 light chain 3 beta) showed a downward trend, implying autophagy degradation and suggesting that PAK-1 was involved in cell autophagy (Wang et al., 2017a). However, there are no more studies on the relationship between PAK-1 and autophagy. Although bFGF has been shown to activate PAK-1, additional relevant reports and studies are lacking (Wary, 2003).

In this study, bFGF was loaded with thermosensitive poloxamer hydrogel to treat injured facial nerves, and it was uniformly distributed and slowly released at the site of the injury. We speculated that bFGF facilitated the recovery of nerve injury by activating the PAK1 pathway to promote autophagy and inhibit apoptosis. Our study provided a new target and therapeutic strategy for the treatment of peripheral nerve injury by bFGF.

## Methods and Materials

### Reagents

We obtained bFGF lyophilized powder from the School of Pharmacy, Wenzhou Medical University (Wenzhou, China). Poloxamer 407 (P2443) was obtained from Sigma-Aldrich (Shanghai, China). Inhibitor targeting PAK1 activation-3 (IPA-3) and Bafilomycin A1 (Baf A1) were obtained from Selleck Chemicals (42521-82-4, 88899-55-2, Shanghai, China). Cy5.5-NHS was obtained from Aladdin (1469277-96-0, Shanghai, China). The primary and secondary antibodies used in this study are listed here. Rabbit monoclonal anti-PAK1 (ab223849), rabbit polyclonal anti-Bcl2 (ab196495), mouse monoclonal anti-P62 (ab56416), rabbit polyclonal anti-Beclin1 (ab62557), donkey anti-mouse IgG H&L Alexa Fluor 488-conjugated secondary antibody (ab150105), donkey anti-rabbit IgG H&L Alexa Fluor 647-conjugated secondary antibody (ab150075) were obtained from Abcam. Rabbit polyclonal anti-Myelin basic protein (MBP, 10458-1-AP), mouse monoclonal anti-S100 (66616-1-Ig), mouse monoclonal anti-GAPDH (60004-1-Ig), horseradish peroxidase (HRP)-conjugated Affinipure Goat Anti-Mouse IgG (H + L) (SA00001-1), and HRP-conjugated Affinipure Goat Anti-Rabbit IgG (H + L) (SA00001-2) were obtained from Proteintech. Rabbit polyclonal phospho-PAK1 (Thr423) (AF4463) was obtained from Affinity. Rabbit anti-BAX (#2772), and rabbit anti-Cleaved Caspase-3 (#9661) were obtained from Cell Signaling Technology. Rabbit polyclonal anti-LC3B (L7543) was purchased from Sigma-Aldrich. Rabbit polyclonal anti-ATG5 (autophagy-related protein 5, AP6026) was purchased from Bioworld.

### The Fabrication and Characterization of P-bFGF Hydrogel

Poloxamer 407 and bFGF powder were mixed in saline and stirred gently at 4°C. The final concentration of the P-bFGF solution was 200 mg/ml for poloxamer and 0.1 mg/ml (or 0.3 mg/ml) for bFGF. The P-bFGF solution was lyophilized, fixed to a copper sheet, and sprayed with gold to take shape. Its microstructure was observed using a scanning electron microscope (SEM, Regulus8230; HITACHI, Tokyo, Japan) and its elemental composition was determined by SEM-EDS (energy-dispersive X-ray spectroscopy).

### Cell Culture and Treatment

The Schwann cell line RSC96 (ScienCell, Shanghai, China) was cultured in Dulbecco’s Modified Eagle Medium (DMEM)containing 10% fetal bovine serum and 1% penicillin-streptomycin solution in an incubator at 37°C and 5% CO_2_. The cells were seeded in a six-well plate at an initial density of 2 × 10^5^ cells/mL. To determine the effect of P-bFGF, we divided the RSC96 cells into six groups: control group, H_2_O_2_ group, poloxamer group, bFGF group, P-bFGF group and IPA-3 group. The control group was cultured in normal DMEM, while the other groups were stimulated with 500 μmol H_2_O_2_ for 2 h. Four hours before H_2_O_2_ stimulation, the poloxamer group, bFGF group, and P-bFGF group were pretreated with poloxamer (200 mg/ml), bFGF (0.1 mg/ml), and P-bFGF (0.1 mg/ml) respectively. While the IPA-3 group was treated with P-bFGF (0.1 mg/ml) and IPA-3 (25 μM). To determine how bFGF and IPA-3 affect autolysosomal flux, we divided the RSC96 cells into seven groups: control group, H_2_O_2_ group, H_2_O_2_+bFGF group, H_2_O_2_+Baf group, H_2_O_2_+bFGF + Baf group, H_2_O_2_+bFGF + Baf + IPA-3 group, and H_2_O_2_+bFGF + IPA-3 group. We treated the cells with Baf A1 (1 nM) 4 h before H_2_O_2_ stimulation.

### Peripheral Nerve Injury Animal Model and Tissue Preparation

Male Sprague Dawley (SD) rats (200–220 g) were purchased from Charles River Laboratories (Beijing, China). The animal experiment in this study was approved by the Animal Experiment Ethics Committee of Wenzhou Medical University. The FNI model was estabilished following a previous study (Xia et al., 2020). Briefly, the rats were anesthetized by an intraperitoneal injection of 1% pentobarbital sodium (40 mg/kg). We exposed the main trunk of the left facial nerve 0.5 cm away from the stylomastoid foramen, and clamped a nerve (∼2 mm long) for 60 s with a hemostatic forceps. The rats were randomly divided into five groups (*n* = 10 per group): sham group, FNI group, poloxamer group (FNI treated with poloxamer), bFGF group (FNI treated with bFGF) and P-bFGF group (FNI treated with P-bFGF). The poloxamer group and P-bFGF group were injected with 15 μL poloxamer (200 mg/ml) and P-bFGF (200 mg/ml poloxamer and 0.3 mg/ml bFGF) on D0, respectively. The sham group, FNI group and bFGF group were injected with 15 μL of normal saline, normal saline, and bFGF (0.1 mg/ml) on D0, D3, and D7, respectively. On D7, five rats in each group were sacrificed. Facial nerves (1.5 cm long), including those from injured sites, were dissected and immediately stored in liquid nitrogen for western blotting. On D14, four to five rats in each group were sacrificed. The facial nerve was fixed in 4% paraformaldehyde (PFA) overnight, dehydrated with gradient absolute ethanol, and embedded in paraffin. The sample was cut into sections (5 μm thick) using a microtome (HistoCore AUTOCUT; Leica, Wetzlar, Germany).

### Fluorescence Labeling of bFGF With Cy5.5-NHS and Fluorescence Imaging *In Vivo*

To track the distribution and residence time of bFGF in the *in vivo* experiment, bFGF used in both P-bFGF and bFGF solutions was labeled with Cy5.5-NHS (Zhao et al., 2014). Briefly, after Cy5.5-NHS was mixed with bFGF (mass ratio 1:100), they were stirred overnight at 4°C in the dark. The unconjugated Cy5.5-NHS was eliminated through dialysis and gel chromatography, and the Cy5.5-bFGF solution was freeze-dried to obtain the Cy5.5-bFGF powder.

The Cy5.5-conjugated P-bFGF and bFGF were used to treat FNI rats. At 0, 6 h, 1, 3, and 7 day, the bioluminescence intensities of the two groups were recorded using an *in vivo* imaging instrument (IVIS Lumina XRMS, Shanghai, China). The luminous intensity was analyzed and quantified by the Living Image^®^ Software.

### Facial Nerve Functional Scoring

According to the method described by Wang et al. (2006), Blink reflex (BR), Vibrissae movement (VM) and tip position (TP) are the three main aspects to assess facial nerve function. For BR, the air was discharged 2 cm away from the injured eye quickly with a 5 ml syringe, and the eyelid movement of the rats was scored with 0 points for no difference between the two sides, 1 point for delayed action on the injured side, and 2 points for unclosed eyelid on the injured side. For VM, bilateral beard movement was counted for 30 s with 0 points for no difference between the two sides, 1 point for weakened movement on the injured side, and 2 points for the loss of beard movement. For TP, 0 points represented nose tip centered, and 1 point for nose tip not centered. The FNI model was considered to be successfully established when the score was 5. After modeling, the facial nerve function of rats was evaluated on D0, D3, D7 and D14, respectively. H&E and Masson’s staining were also performed to confirm that the modeling was successful.

### Hematoxylin and Eosin and Masson’s Trichrome Staining

According to the manufacturer’s protocol, the prepared sections were stained by H&E or Masson’s trichrome staining. Initially, the sections were dried at 56°C for 30 min, then immersed in xylene to dewax, and hydrated with gradient absolute ethanol and pure water. Then, different staining reagents were added to the sections. For H&E staining, the sample was immersed in hematoxylin for 7 min and eosin for 2 min. For Masson’s trichrome staining, the nucleus of the section was stained with hematoxylin for 5 min, and Ponceau/Acid Fuchsin solution was added to cover the entire tissue for 5 min. Then, the section was stained in 1% phosphomolybdic acid solution for 30 s and stained in aniline blue reagent for 1 min. Finally, the glass slide was mounted with neutral resin. The stained section was observed and imaged using an inverted microscope (DMILLED; Leica).

### Immunofluorescence Staining

The tissue sections were subjected to antigen repair with high temperature and pressure. The cells were fixed in 4% PFA at room temperature for 30 min and then incubated with 0.5% Triton X-100 for 15 min. Then, the tissue sections or cells were blocked with 5% BSA for 30 min, and the tissues or cells were incubated with primary antibodies against MBP (1:100), S100 (1:100), LC3B (1:300), and Cleaved Caspase-3 (1:400) at 4°C overnight. After washing, these samples were incubated with Alexa Fluor conjugated secondary antibody (1:200) at room temperature for 1 h, and finally mounted with DAPI. Fluorescence images were captured using a laser confocal scanning fluorescence microscope (TCS SP8; Leica), and the fluorescence intensity was quantified by the ImageJ software.

### Western Blot

The facial nerve was placed in the RIPA lysis buffer containing 1% PMSF and lysed in a multi-sample tissue homogenizer (Bionoon-24LD; BIONOON, Shanghai, China) for 10 min. The RSC96 cells were placed in the RIPA lysis buffer containing 1% PMSF. Subsequently, the homogenized facial nerve or cell suspension was centrifuged at 12,000 rpm at 4°C for 15 min, and the protein concentration in the supernatant was quantified by the BCA reagent. The same amount of total protein was separated by SDS (Sodium dodecyl sulfate)-polyacrylamide gel electrophoresis and transferred to a PVDF (polyvinylidene fluoride) membrane. The membrane was blocked using skim milk and incubated overnight with primary antibodies (including GAPDH, Bcl2, BAX, beclin1, LC3B, ATG5, p62, p-PAK1 and PAK1) at 4°C. On the following day, the membrane was incubated with a secondary antibody conjugated with HRP for 2 h at room temperature. An enhanced chemiluminescence kit was used to detect the protein band, and the band density was quantified using the ImageJ software.

### Statistical Analysis

The data were analyzed using Graphpad Prism9 statistical software. One-way ANOVA was performed for analyzing data with Figures 2D,E, 3B,D–G, 4B,D,E, 5B–G,I,J, 6B,D,E. For two variables, two-way ANOVA was performed (Figure 2A). The difference was considered to be statistically significant at *p* < 0.05.

## Results

### Poloxamer Hydrogel Loaded With Basic Fibroblast Growth Factor Maintained Thermal Sensitivity and the Porous Structure and P-bFGF Constantly Released bFGF *In Situ*

**FIGURE 1:**
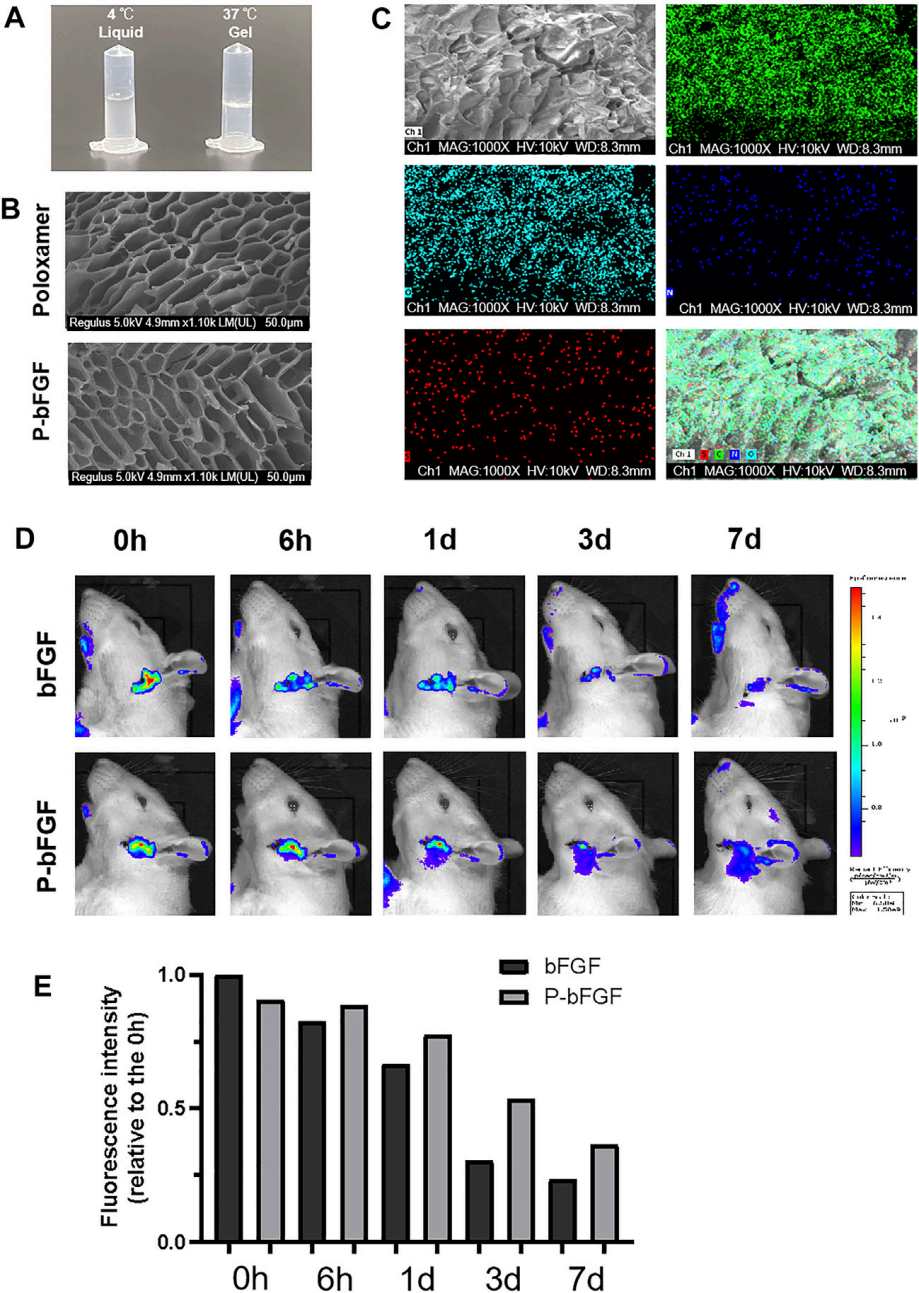
Thermosensitivity, microstructure, relevant element analysis and *in vivo* local residence time of P-bFGF hydrogel. **(A)** The state of P-bFGF at 4 and 37°C. **(B)** The SEM image of poloxamer and P-bFGF hydrogel morphology; scale bar = 50 μm. **(C)** The SEM-EDS image analysis of P-bFGF hydrogel morphology. Channel 1 (Ch1), Channel C, Channel O, Channel N and Channel S were merged as one Channel. Scale bar = 50 μm. **(D)** Representative bioluminescence imaging of rats in different treatment groups at different times. **(E)** Quantitative calculation of the luminous intensity.

To gelate at body temperature, we prepared a poloxamer solution with a concentration of 17% (w/w), which was found to be the most suitable in previous studies (Wang et al., 2017b). The poloxamer was in a liquid form at 4°C and turned into a hydrogel at 37°C (Figure 1A). Moreover, the ultrastructure of the poloxamer hydrogel was observed by SEM (Figure 1B). The results showed that the internal crosslinking of hydrogel formed a loose porous network, and the internal porous network was unchanged after bFGF was loaded. SEM-EDS was performed to analyze the elements of P-bFGF. The C and O elements represented poloxamer hydrogel, and the N and S elements represented bFGF. We found that bFGF was evenly distributed in the poloxamer hydrogel (Figure 1C).

To observe whether P-bFGF can continuously release bFGF *in situ*, Cy5.5-bFGF was used to treat FNI rats. Through *in vivo* imaging, we discovered that in the bFGF group, the bFGF solution diffused in the tissue, while in the P-bFGF group, bFGF was attached to the facial nerve, which allowed us to see the contour of the facial nerve clearly. With the passage of time, we found that the bioluminescence intensity of bFGF decreasd, while the bioluminescence intensity of the P-bFGF group rats became stronger than that of the bFGF group rats (Figure 1D). The quantification of bioluminescence intensity showed the same trend (Figure 1E), which proved that P-bFGF constantly released bFGF in the injured nerve *in situ*, and maximized the therapeutic effect of bFGF *in vivo*. The results showed that P-bFGF was suitable for local uniform delivery and slow-release after nerve injury *in vivo*.

### P-bFGF Improved Functional Recovery of Early Facial Nerve Injury

**FIGURE 2:**
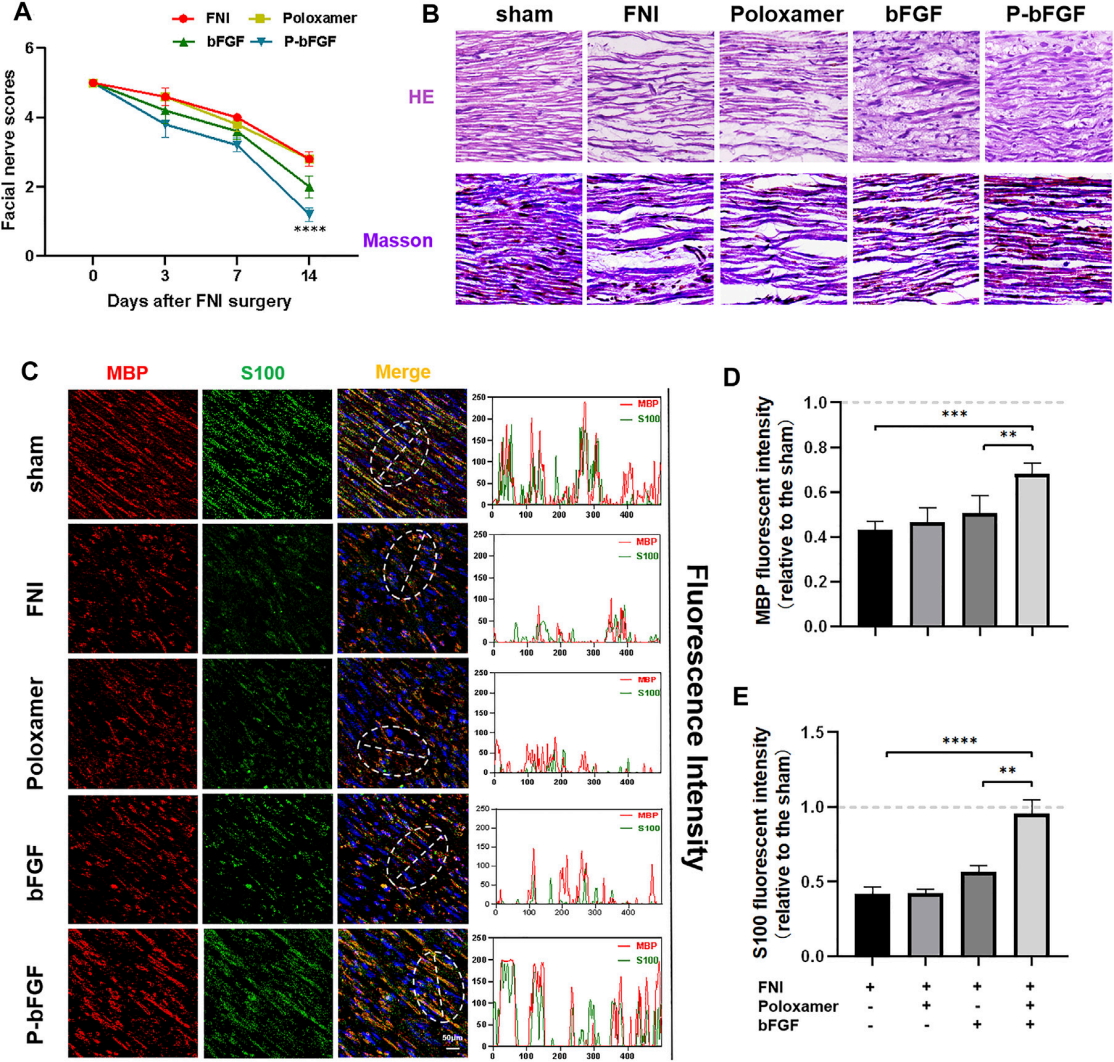
The functional and histological recovery of the injured nerve 2 weeks after treatment. **(A)** Facial nerve function scoring was performed 0, 3, 7, and 14 days after surgery. The data were expressed as mean ± SEM (*n* = 5); P-bFGF group vs. FNI group: ****p < 0.0001, P-bFGF group vs. poloxamer group: ****p < 0.0001. **(B)** Injured nerves were stained with H&E and Masson’s stain, respectively; scale bar = 100 μm. **(C)** Confocal laser scanning micrograph of immunofluorescence staining with anti-MBP (red) and anti-S100 (green) antibodies; scale bar = 50 μm. The co-localization of MBP and S100 was shown by a plot profile analysis in Image J. **(D,E)** Quantitative calculation of the mean of the MBP and S100 fluorescence. The data were expressed as mean ± SEM (*n* = 4); ****p < 0.0001, ***p < 0.001 and **p < 0.01. A dashed line at 1.0 represented sham group.

To determine whether P-bFGF treatment promoted the recovery of facial nerve function, facial nerve function scoring was performed 0, 3, 7 and 14 days after surgery. On D0 after surgery, the score in each group was 5 points, suggesting that the model was successfully established. With time, the facial nerve function improved in all the groups, but the facial nerve function scoring revealed that the scores of the P-bFGF group and bFGF group were lower than those of the FNI group and poloxamer group. P-bFGF group was significantly lower than FNI group (****p < 0.0001) and poloxamer group (****p < 0.0001) and bFGF group was not significantly lower than FNI group and poloxamer group, which might be attributed to the long-lasting protection of the poloxamer hydrogel for bFGF bioactivity and slow drug release at the site of the facial lesion (Figure 2A). In summary, poloxamer alone had no therapeutic effect. This was also reflected in all the experiments that followed. We found that bFGF alone may not promote the recovery of the facial nerve function. P-bFGF, a mixture of bFGF and poloxamer, can significantly promoted the early functional recovery of facial nerve injury.

### P-bFGF Accelerated Facial Nerve Regeneration and Functional Protein Expression

Hematoxylin & Eosin and Masson’s staining were used to evaluate the histological recovery of injured nerves in each group. Both H&E and Masson’s staining demonstrated that the nerve fibers in the FNI group and the poloxamer group were sparse and atrophic. The abnormal morphologies in the bFGF group and P-bFGF group improved. The nerve fibers in the P-bFGF group regenerated significantly and more regularly compared to those of the bFGF group (Figure 2B). This suggested that P-bFGF can promote the recovery of histological morphology of injured nerve fibers.

The S100 maker of Schwann cells can regulate the metabolism, movement and proliferation of cells. The presence of MBP (myelin basic protein) indicates myelin formation. Double IF staining of S100 and MBP showed that the functional protein expression and myelin density of the P-bFGF group and bFGF group were significantly higher than those of the FNI group and poloxamer group, and P-bFGF was higher than bFGF (Figure 2C). The qualitative fluorescence co-localization expression analysis and respective quantitative analysis of S100 and MBP showed the similar trend (Figures 2D,E). The results indicated that P-bFGF upregulated the functional protein S100 of Schwann cells and boosted the remyelination of the SCs.

### P-bFGF Promoted Nerve Repair by Activating Autophagy and Inhibiting Apoptosis

**FIGURE 3:**
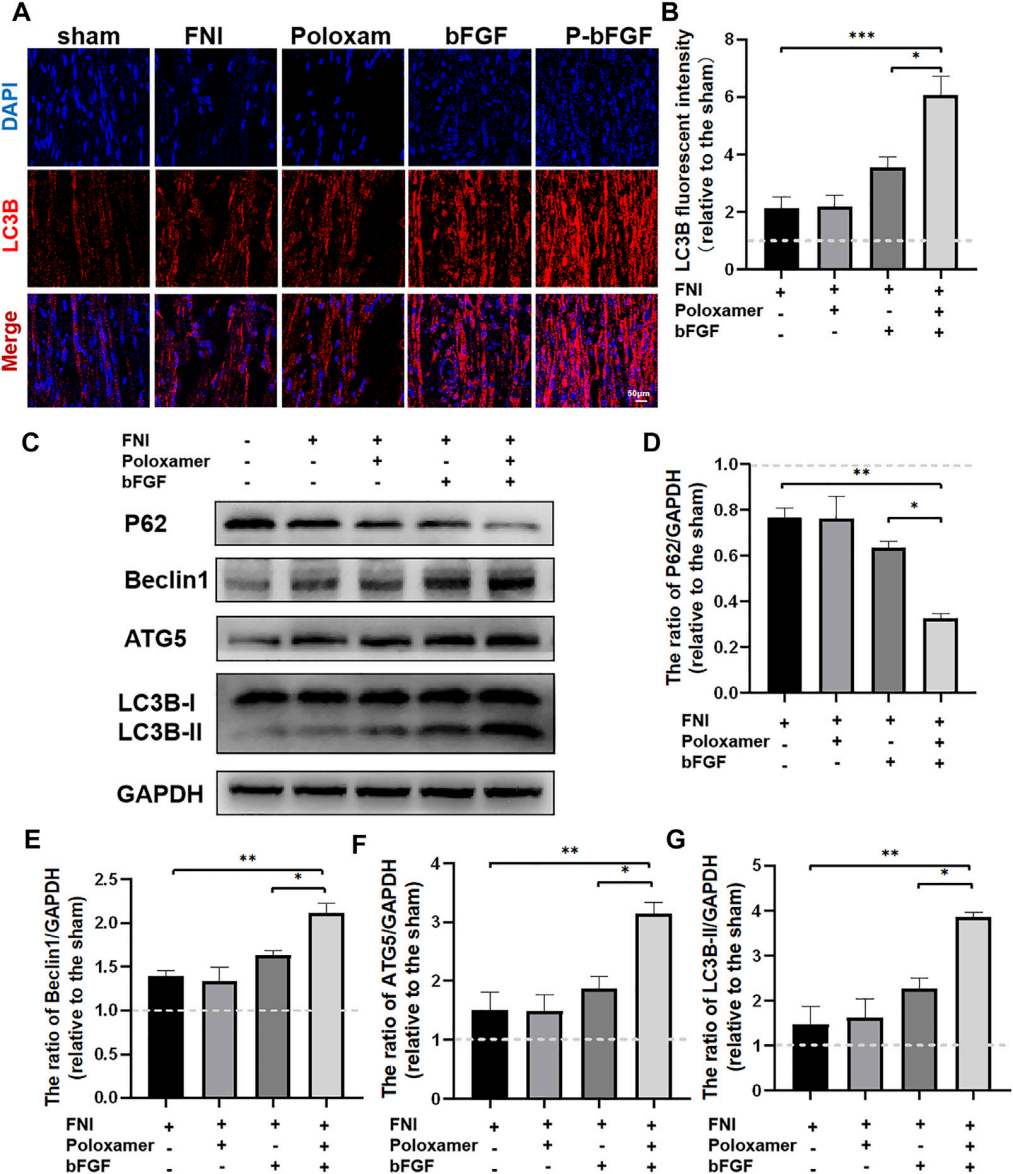
P-bFGF activated autophagy in order to promote facial nerve repair. **(A)** Representative immunofluorescence staining of LC3B of injured nerves 2 weeks after treatment; scale bar = 50 μm. **(B)** Quantitative calculation of the mean of LC3B fluorescence. The data were expressed as mean ± SEM (*n* = 4); FNI group vs. P-bFGF group: ***p < 0.001, bFGF group vs. P-bFGF group: *p < 0.05. **(C–G)** Representative western blot images of P62, Beclin1, ATG5, and LC3B-II expression in FNI 7 days after treatment and quantification of protein levels. The data were expressed as mean ± SEM (*n* = 3); FNI group vs. P-bFGF group: **p < 0.01, bFGF group vs. P-bFGF group: *p < 0.05. A dashed line at 1.0 represented sham group.

To determine whether P-bFGF activated autophagy for facial nerve repair, we performed tissue IF staining and WB to detect the expression of autophagy-related proteins (including LC3B, Beclin1, P62, and ATG5). The IF staining showed that FNI slightly increased the fluorescence intensity of LC3B, and the bFGF and P-bFGF treatment further enhanced the fluorescence intensity of LC3B, the fluorescence intensity of the P-bFGF group was significantly higher than that of the bFGF group (Figure 3A). The quantitative analysis of fluorescence intensity showed a similar trend (Figure 3B). The WB results showed that the expression trends of LC3B-II, Beclin1, and ATG5 were consistent with the fluorescence intensity of LC3B, while the expression trend of P62 was the opposite (Figures 3C–G). These results indicated that P-bFGF activated autophagy in FNI.

**FIGURE 4:**
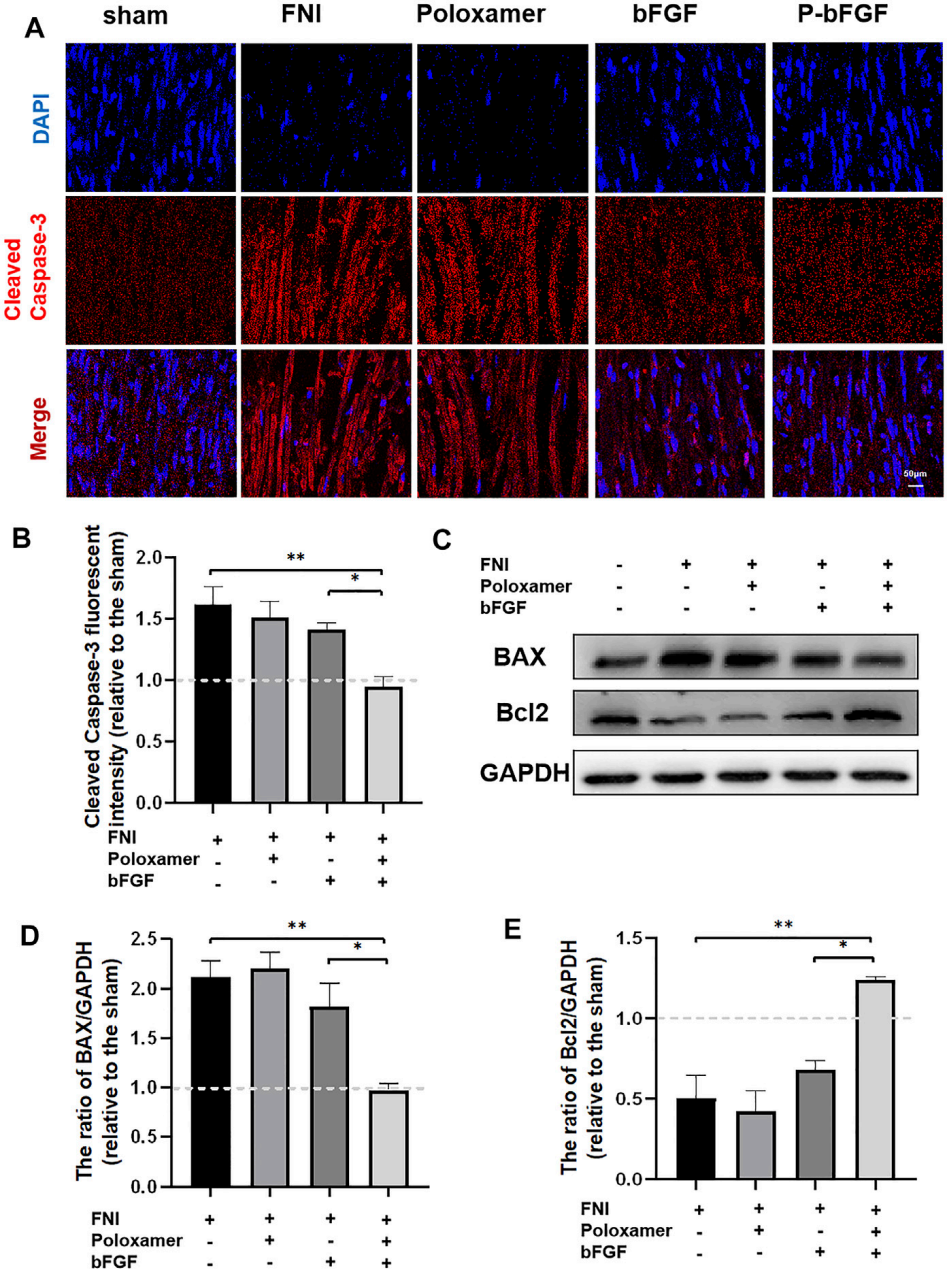
P-bFGF inhibited apoptosis in order to promote facial nerve repair. **(A)** Representative immunofluorescence staining of Cleaved Caspase-3 of the injured nerves 2 weeks after treatment; Scale bar = 50 μm. **(B)** Quantitative calculation of the mean of Cleaved Caspase-3 fluorescence. The data were expressed as mean ± SEM (*n* = 4); FNI group vs. P-bFGF group: **p < 0.01, bFGF group vs. P-bFGF group: *p < 0.05. **(C–E)** Representative western blot images of BAX and Bcl2 expression in FNI 7 days after treatment and quantification of protein levels. The data were expressed as mean ± SEM (*n* = 3); FNI group vs. P-bFGF group: **p < 0.01, bFGF group vs. P-bFGF group: *p < 0.05. A dashed line at 1.0 represented sham group.

To further determine whether P-bFGF treatment contributed to cell survival, we also measured the expression of apoptosis-related proteins by IF staining (Cleaved Caspase-3) and western blotting (BAX and Bcl2), and assessed the ability of P-bFGF to activate autophagy, which can help to reduce apoptosis after FNI. The pro-apoptosis protein Cleaved Caspase-3 and BAX were downregulated, while the anti-apoptosis protein Bcl2 was upregulated in the bFGF group and P-bFGF group, compared to their levels in the FNI group (Figures 4A,C). The quantitative analysis of fluorescence intensity and WB gray value showed a similar trend (Figures 4B,D,E). The above results confirmed that P-bFGF can promotes FNI repair by reducing apoptosis for the survival of nerve cells.

### P-bFGF Activated Autophagy and Maintained the Fluent Autophagic Flux *In Vitro* Through the PAK-1 Signaling Pathway

**FIGURE 5:**
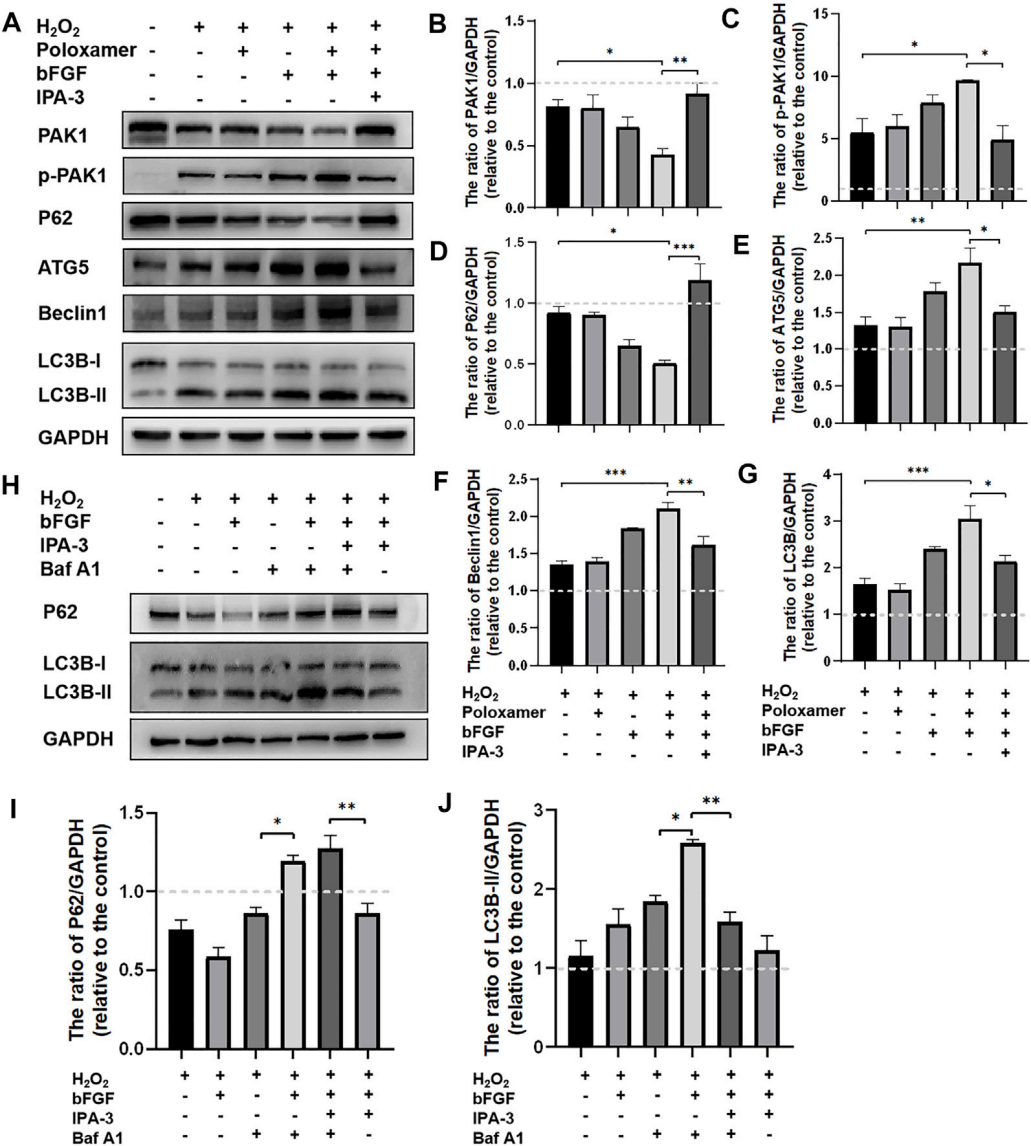
P-bFGF activated the PAK1 pathway to promote autophagy of SCs. **(A)** Representative western blot images of PAK1, p-PAK1, P62, ATG5, Beclin1 and LC3B expression. **(B)** Quantification of the levels of PAK1 protein; H_2_O_2_ group vs. P-bFGF group: *p < 0.05, P-bFGF group vs. IPA-3 group: **p < 0.01. **(C)** Quantification of the levels of p-PAK1 protein; H_2_O_2_ group vs. P-bFGF group: *p < 0.05, P-bFGF group vs. IPA-3 group: *p < 0.05. **(D)** Quantification of the levels of P62 protein; H_2_O_2_ group vs. P-bFGF group: *p < 0.05, P-bFGF group vs. IPA-3 group: ***p < 0.001. **(E)** Quantification of the levels of ATG5 protein; H_2_O_2_ group vs. P-bFGF group: **p < 0.01, P-bFGF group vs. IPA-3 group: *p < 0.05. **(F)** Quantification of the levels of the Beclin1 protein; H_2_O_2_ group vs. P-bFGF group: ***p < 0.001, P-bFGF group vs. IPA-3 group: **p < 0.01. **(G)** Quantification of the levels of the LC3B-II protein; H_2_O_2_ group vs. P-bFGF group: ***p < 0.001, P-bFGF group vs. IPA-3 group: *p < 0.05. **(H)** Representative western blot images of P62 and LC3B expression. **(I)** Quantification of the levels of the P62 protein; H_2_O_2_+Baf group vs. H_2_O_2_+bFGF + Baf group: *p < 0.05, H_2_O_2_+bFGF + Baf + IPA-3 group vs. H_2_O_2_+bFGF + IPA-3 group: **p < 0.01. **(J)** Quantification of the levels of the LC3B-II protein; H_2_O_2_+Baf group vs. H_2_O_2_+bFGF + Baf group: *p < 0.05, H_2_O_2_+bFGF + Baf group vs. H_2_O_2_+bFGF + Baf + IPA-3 group: **p < 0.01. The data above were expressed as mean ± SEM (*n* = 3). A dashed line at 1.0 represented control group.

To study the molecular mechanism underlying the autophagy activation of P-bFGF, we performed the above treatment on different groups of RSC96 cells. The expressions of pathway-related proteins (including PAK1 and p-PAK1) and autophagy-related proteins (including LC3B, Beclin1, P62, and ATG5) were detected. Through the WB assay, we found that the phosphorylation level of PAK1 increased in the H_2_O_2_ group and the poloxamer group. The phosphorylation level of PAK1 further increased in the bFGF group and P-bFGF group. IPA-3 partially reversed the effect in the P-bFGF group. The expression of PAK1 was opposite to that of p-PAK1 (Figures 5A–C). This showed that bFGF activated the PAK1 pathway, but IPA-3 inhibited the activation of bFGF in the PAK1 pathway. The expressions of ATG5, Beclin1 and LC3B-II in the P-bFGF group significantly increased compared with the H_2_O_2_ group (**p < 0.01, ***p < 0.001 and ***p < 0.001, respectively), while the use of IPA-3 significantly reversed the treatment of P-bFGF (*p < 0.05, **p < 0.01 and *p < 0.05, respectively). The expression of P62 showed an opposite trend to the expression of ATG5, Beclin1 and LC3B-II (Figures 5A,D–G). To confirm the continuous autolysosomal function in the cells treated by bFGF and determine how IPA-3 affected autolysosomal flux. We used Baf A1, an inhibitor of late autophagy, to treat RSC96 cells after the administration of bFGF. Baf A1 further raised the expression of LC3B-II and P62, while IPA-3 reversed the expression of LC3B-II and further increased P62 (Figures 5H–J). This indicated that the autophagic flux was activated by bFGF and blocked by IPA-3, which suggested that P-bFGF activated autophagy through the PAK1 pathway.

### P-bFGF Inhibited Apoptosis *In Vitro* Through the PAK-1 Signaling Pathway

**FIGURE 6:**
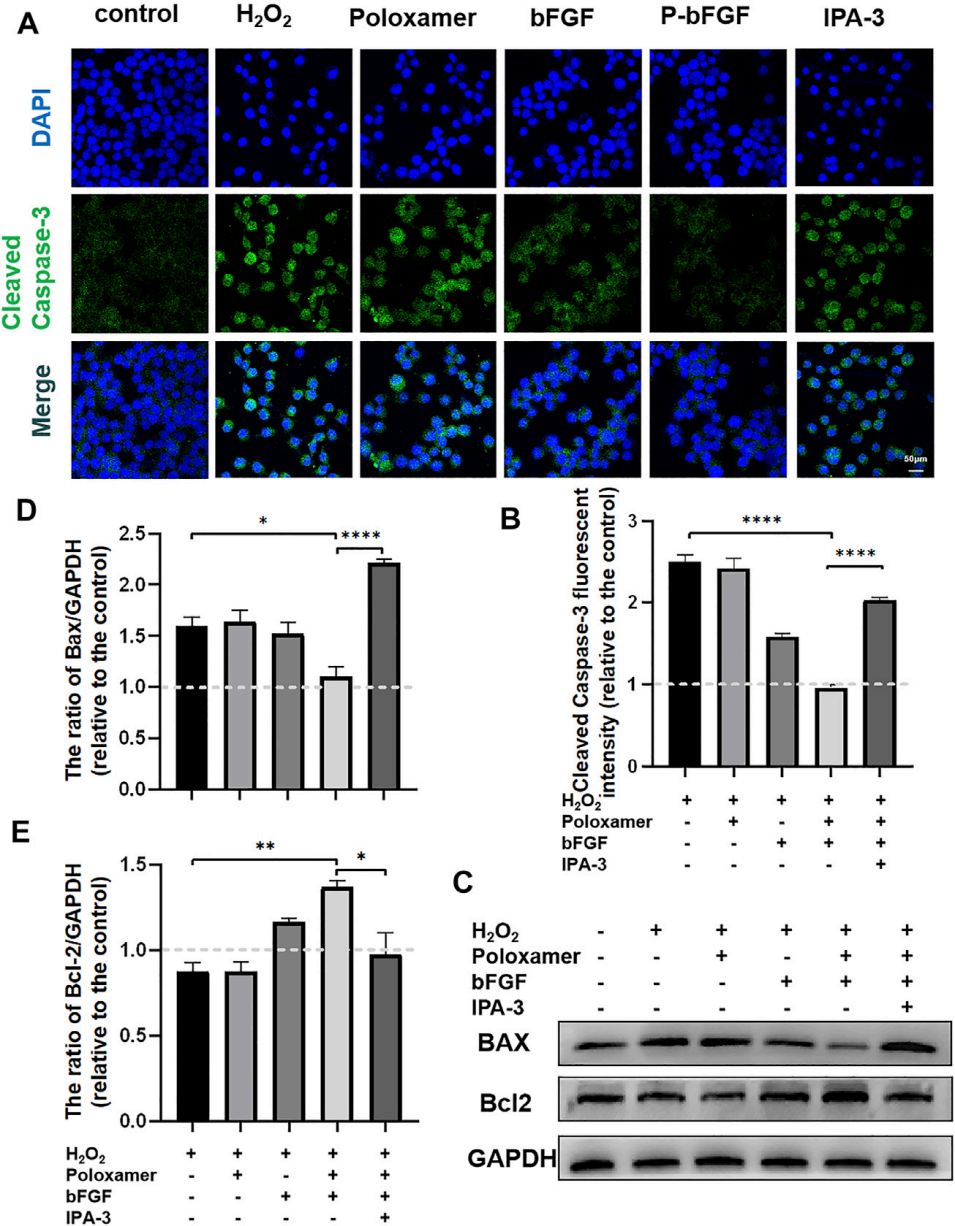
P-bFGF activated PAK1 and inhibited the apoptosis of SCs. **(A)** Representative immunofluorescence staining of Cleaved Caspase-3 of SCs after treatment; scale bar = 50 μm. **(B)** Quantitative calculation of the mean of Cleaved Caspase-3 fluorescence. The data were expressed as mean ± SEM (*n* = 4); H_2_O_2_ group vs. P-bFGF group: ****p < 0.0001, P-bFGF group vs. IPA-3 group: ****p < 0.0001. **(C)** Representative western blot images of the BAX and Bcl2 expression in SCs after treatment. **(D)** Quantification of the levels of the BAX protein; H_2_O_2_ group vs. P-bFGF group: *p < 0.05, P-bFGF group vs. IPA-3 group: ****p < 0.0001. **(E)** Quantification of the levels of the Bcl2 protein; H_2_O_2_ group vs. P-bFGF group: **p < 0.01, P-bFGF group vs. IPA-3 group: *p < 0.05. The data above were expressed as mean ± SEM (*n* = 3). A dashed line at 1.0 represented control group.

To further confirm the molecular mechanism underlying the reduction of P-bFGF in H_2_O_2_-induced apoptosis of SCs, cell IF staining was performed to detect the expression of Cleaved Caspase-3, and WB was performed to detect the expressions of BAX and Bcl2. The fluorescence intensity of the pro-apoptosis protein Cleaved Caspase-3 in the P-bFGF group was significantly lower than that in the H_2_O_2_ group (****p < 0.0001), and IPA-3 treatment reversed the effect of the P-bFGF treatment (****p < 0.0001, Figures 6A,B). The grayscale value of the pro-apoptosis protein BAX was significantly lower than that in the H_2_O_2_ group (*p < 0.05), and IPA-3 treatment reversed the effect of the P-bFGF treatment (****p < 0.0001, Figures 6C,D). Moreover, the grayscale value trend of the anti-apoptosis protein Bcl2 was opposite to that of BAX. The value of the P-bFGF group was significantly higher than that of the H_2_O_2_ group (**p < 0.01), and IPA-3 treatment reversed the effect of the P-bFGF treatment (*p < 0.05, Figures 6C,E). These results indicated that P-bFGF protected SCs from being apoptotic via the PAK1 pathway.

## Discussion

**FIGURE 7:**
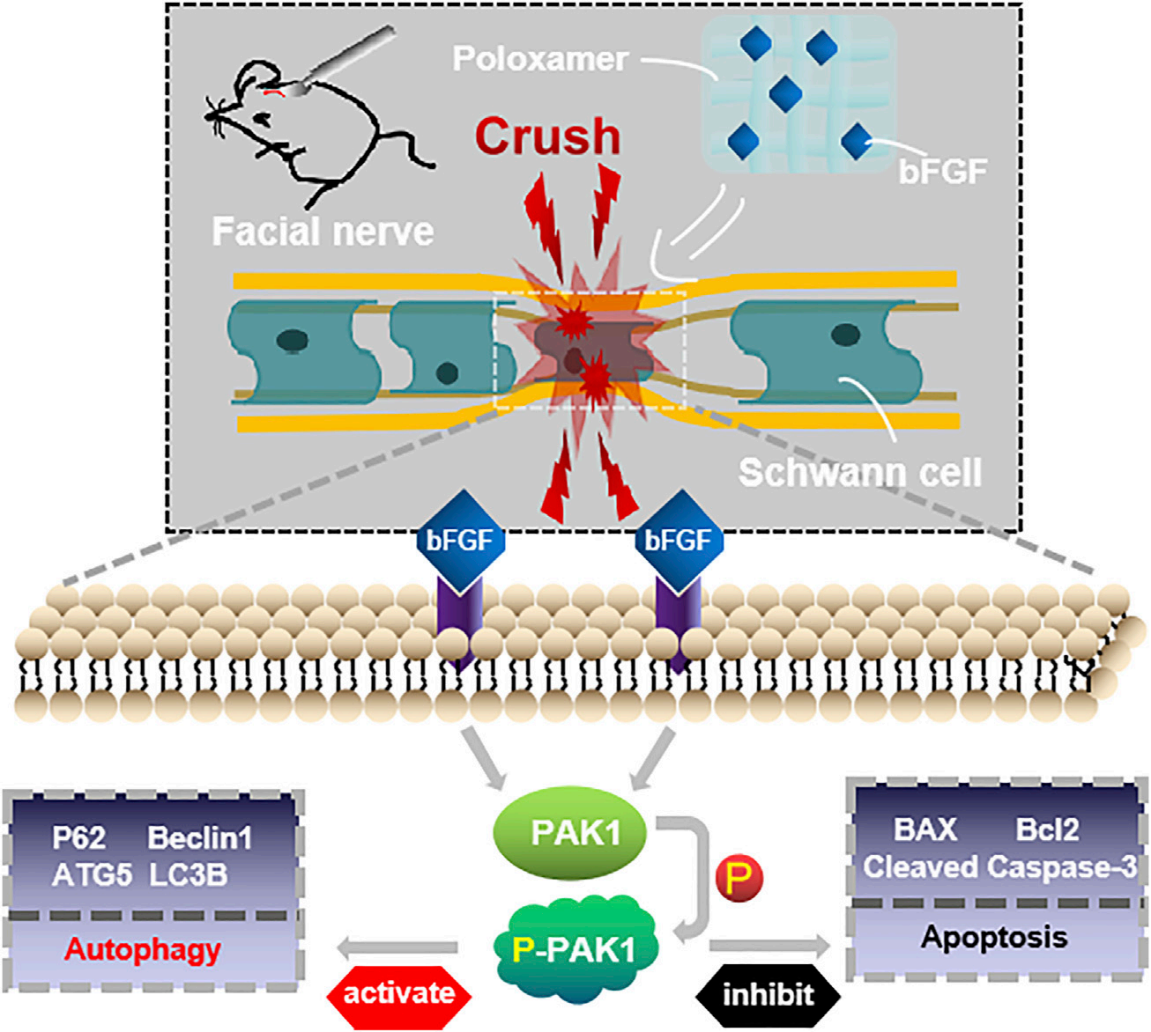
Conceptualization. The treatment of injured facial nerve with thermosensitive poloxamer loaded with bFGF. The bFGF was released steadily and continuously at the original site of injury, and bFGF treated the injured facial nerve by activating the PAK1 signaling pathway to promote autophagy and inhibit apoptosis.

The restoration of the function of the damaged facial nerves to a near-normal is challenging. Patients often suffer from partial or permanent disability in sensory and motor nerve function. Exogenous bFGF can promote FNI repair (Li et al., 2018; Jiang et al., 2021), but maintaining the release of bFGF at the appropriate location and time is a major obstacle to effective therapy. We used poloxamer thermosensitive hydrogel with excellent biocompatibility as the carrier. Using the rat FNI model, we found that P-bFGF promoted facial nerve repair by 1) maintaining the survival of nerve cells and inhibiting their apoptosis, 2) activating the autophagy of SCs and promoting the removal of myelin debris, and 3) facilitating axon growth and remyelination. These were consistent with previous studies on other peripheral nerve injuries (Wang et al., 2003; Pollak et al., 2014; Jiang et al., 2021). Through *in vitro* experiments, we found that the molecular mechanism underlying the positive effect of P-bFGF involved the activation of the PAK1 signaling pathway. Our research offers new insight into the role of bFGF in promoting the repair of damaged facial nerves by activating autophagy and inhibiting apoptosis (Figure 7).

Poloxamer hydrogel is an ideal drug delivery tool that is compatible with most tissues, thus, it has a wide range of applications in neuroprotection and regeneration. Poloxamer is in a liquid form at 4°C, making it easy to load bFGF. At body temperature, it transforms into a gel and provides an appropriate mechanical strength (Figure 1A). We found that bFGF could be loaded in large quantities in a three-dimensional network (Figure 1B) and was evenly diffused in these structures (Figure 1C). Therefore, bFGF can be uniformly and steadily released into the injured area over time (Figures 1D,E). We investigated the effects of bFGF with three injections on D0, D3, and D7, but for P-bFGF, only one injection was administered on D0, which showed that P-bFGF had considerable advantages in neuroprotection, regeneration, and functional recovery, and was superior to bFGF (Figures 2A,B). After FNI, the denervated SCs dedifferentiated, migrated, proliferated and transformed into repaired SCs. The repaired SCs removed degenerated myelin debris and secreted neurotrophic factors in order to create a favorable microenvironment for the regeneration of nerve cells (Jessen et al., 2015). Therefore, the regeneration and functional recovery of peripheral nerves are crucial to promote the proliferation and remyelination of SCs in the distal end of the lesion and inhibit their apoptosis (Jessen and Mirsky, 2016). S100 is a functional protein of SCs, whose level in the cell indicates the strength of proliferation. MBP indicates myelination and can represent the degree of remyelination. The results of immunofluorescence double-staining experiments also showed that after the application of bFGF, especially when it was loaded into poloxamer hydrogel, the proliferation and remyelination ability of SCs were enhanced (Figure 2C). Autophagy activation plays an active role in the repair of PNI, and the bFGF-mediated removal of myelin debris is driven by the activation of autophagy (Jiang et al., 2021). In our study, after FNI, the autophagy markers (P62, Beclin1, ATG5, and LC3B) changed in the direction of autophagy activation, which was caused by the obstacle of the fusion of the autophagosome with lysosome (Jiang et al., 2021). We discovered that bFGF could enhance autophagy, and the effect of P-bFGF treatment was better than that of bFGF treatment (Figures 3A–F). BAX, Bcl2 and Cleaved Caspase-3 were involved in the regulation of apoptosis. bFGF can also upregulate the anti-apoptotic protein Bcl2 and downregulate the pro-apoptotic proteins BAX and Cleaved Caspase-3. Similarly, the effect of P-bFGF treatment was better than that of bFGF treatment (Figures 4A–E).

Previously, PAK1 was shown to play a unique role in brain ontogeny and reconstruction of the neural cytoskeleton. It was also involved in various neurodevelopmental processes, such as neuron migration, neurite growth, neuron polarity, neuron differentiation and axon development (Zhang et al., 2020). PAK1 controls the correct direction, morphology and radial migration of neurons in the developing cerebral cortex (Zhong et al., 2003). In the growth cone of neurons, PAK1 can interact with NCAM (neural cell adhesion molecule), a member of the Ig superfamily, to induce the activation of the PAK1-LIMK1-cofilin pathway and facilitate cytoskeleton remodeling and Filopodia movement, to aid the growth and the direction of development of axons (Li et al., 2013). From *in vitro* cell experiments, we found that the expression of PAK1 decreased, while the expression of p-PAK1 increased after SCs injury. After bFGF and P-bFGF treatment, the expression of PAK1 further decreased, while the expression of p-PAK1 increased. The therapeutic effect of P-bFGF was better than that of bFGF, and the use of the PAK1 inhibitor IPA-3 partially reversed the effect of P-bFGF. We also found that autophagy-related proteins (P62, Beclin1, ATG5, and LC3B) were expressed in the direction of autophagy activation. The protein expression trend coincided with that of *in vivo* experiments, and IPA-3 could partially reverse the role of P-bFGF in autophagy activation (Figures 5A–G). We found that Baf A1 further raised the expression of LC3B-II and P62, while IPA-3 reversed the expression of LC3B-II and further increased P62. This indicated that bFGF could maintain fluent autophagic flux to enhance the level of autophagy through the PAK1 pathway (Figures 5H–J). The level of apoptosis also significantly decreased after P-bFGF treatment. Similar to the *in vivo* experiments, IPA-3 could partially reverse the anti-apoptotic effect of P-bFGF (Figures 6A–E). The above results suggested that the P-bFGF treatment of injured SCs was probably mediated by the activation of the PAK1 pathway, which increased autophagy and the anti-apoptotic effect. PAK1 can regulate the cytoskeleton and internode length to facilitate oligodendrocyte differentiation and myelination (Brown et al., 2021). bFGF induces autophagy activation, maintains the fluent autophagic flux in SCs to degrade myelin sheath fragments, and repairs peripheral nerves in the early stage of injury (Jiang et al., 2021). Moreover, autophagy activation can remove excess cytoplasm in SCs and promote myelin compaction, which plays a key role in the maturation and structural plasticity of SCs (Jang et al., 2015). A study on Alzheimer’s disease found that the activation of the PAK1 signaling pathway can promote the autophagy of microglia, while its inhibitor IPA-3 can lower autophagy (Qi et al., 2021). The activation of PAK1 is closely related to the remodeling of the actin cytoskeleton (Davidson et al., 2021). In 1992, it was established that starved cells treated with actin depolymerization agents did not produce autophagosomes (Aplin et al., 1992). Subsequent studies also showed that actin can co-localize with important autophagy markers (Reggiori et al., 2005; Aguilera et al., 2012). The knockout of the ATG7 gene in mice not only inhibited the formation of autophagosomes but also induced serious defects in actin assembly (Zhuo et al., 2013). Hence, we speculated that bFGF activated the PAK1 signaling pathway to regulate autophagy by remodeling the actin cytoskeleton in FNI treatment. In future experiments, we aim to study how the actin cytoskeleton remodeling is involved in activating autophagy.

Our results indicated that P-bFGF could effectively promote cell proliferation, myelination and functional recovery and also reduce the apoptosis of nerve cells after FNI. The protective effect of P-bFGF on nerves is probably facilitated by the activation of the PAK1 pathway in SCs. The activation of the PAK1 pathway increases autophagy to degrade myelin fragments, promotes myelin formation, and inhibits the apoptosis of SCs. Therefore, our study was intended to find a new therapeutic method and a targeted treatment site for FNI and provide a new strategy for the treatment and mechanism of PNI in the future.

## Data Availability

The original contributions presented in the study are included in the article/Supplementary Material, further inquiries can be directed to the corresponding authors.
