# Supplementary figures and images for: Delivery of Basic Fibroblast Growth Factor Through an In Situ Forming Smart Hydrogel Activates Autophagy in Schwann Cells and Improves Facial Nerves Generation via the PAK-1 Signaling Pathway (part 1 of 2)
Source: Front Pharmacol. 2022 Apr 1;13:778680. doi: 10.3389/fphar.2022.778680 (PMC9011134; doi:10.3389/fphar.2022.778680)

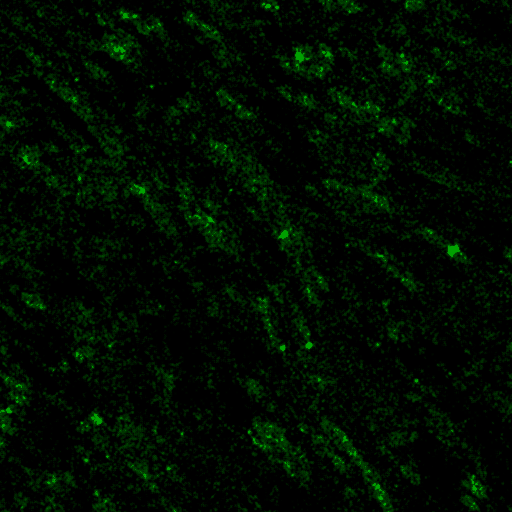

Supplement: Supplementary file 1 [file DataSheet3.ZIP › figure 2C FNI (2).tiff]

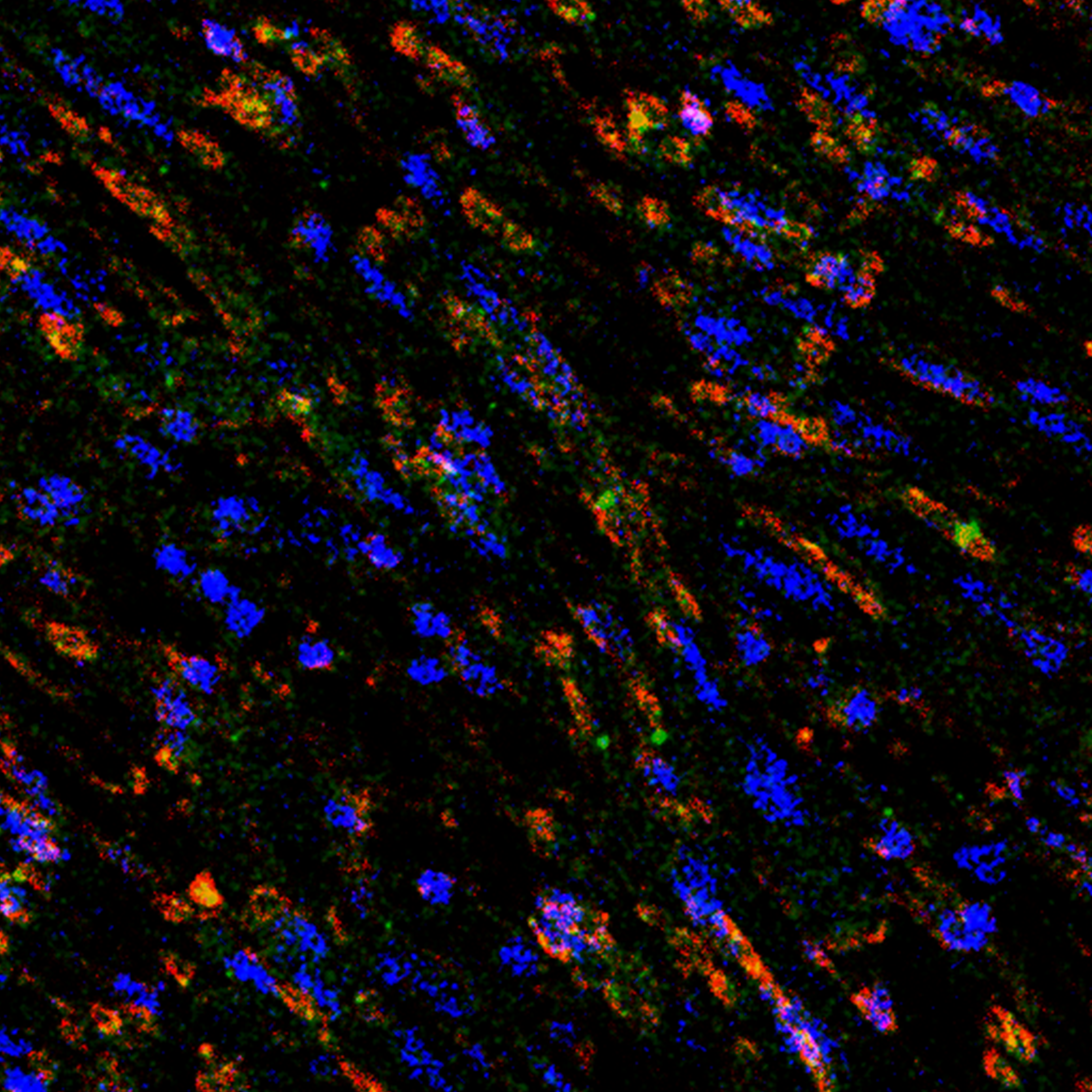

Supplement: Supplementary file 1 [file DataSheet3.ZIP › figure 2C FNI (3).tiff]

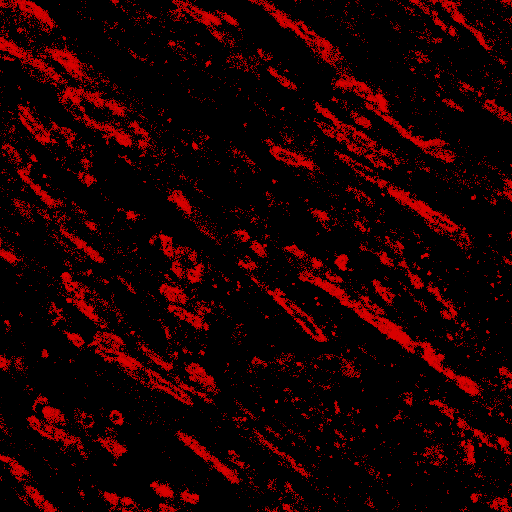

Supplement: Supplementary file 1 [file DataSheet3.ZIP › figure 2C P-bFGF (1).tiff]

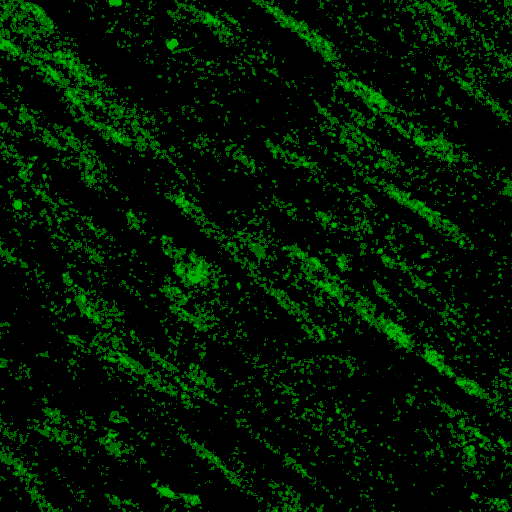

Supplement: Supplementary file 1 [file DataSheet3.ZIP › figure 2C P-bFGF (2).tiff]

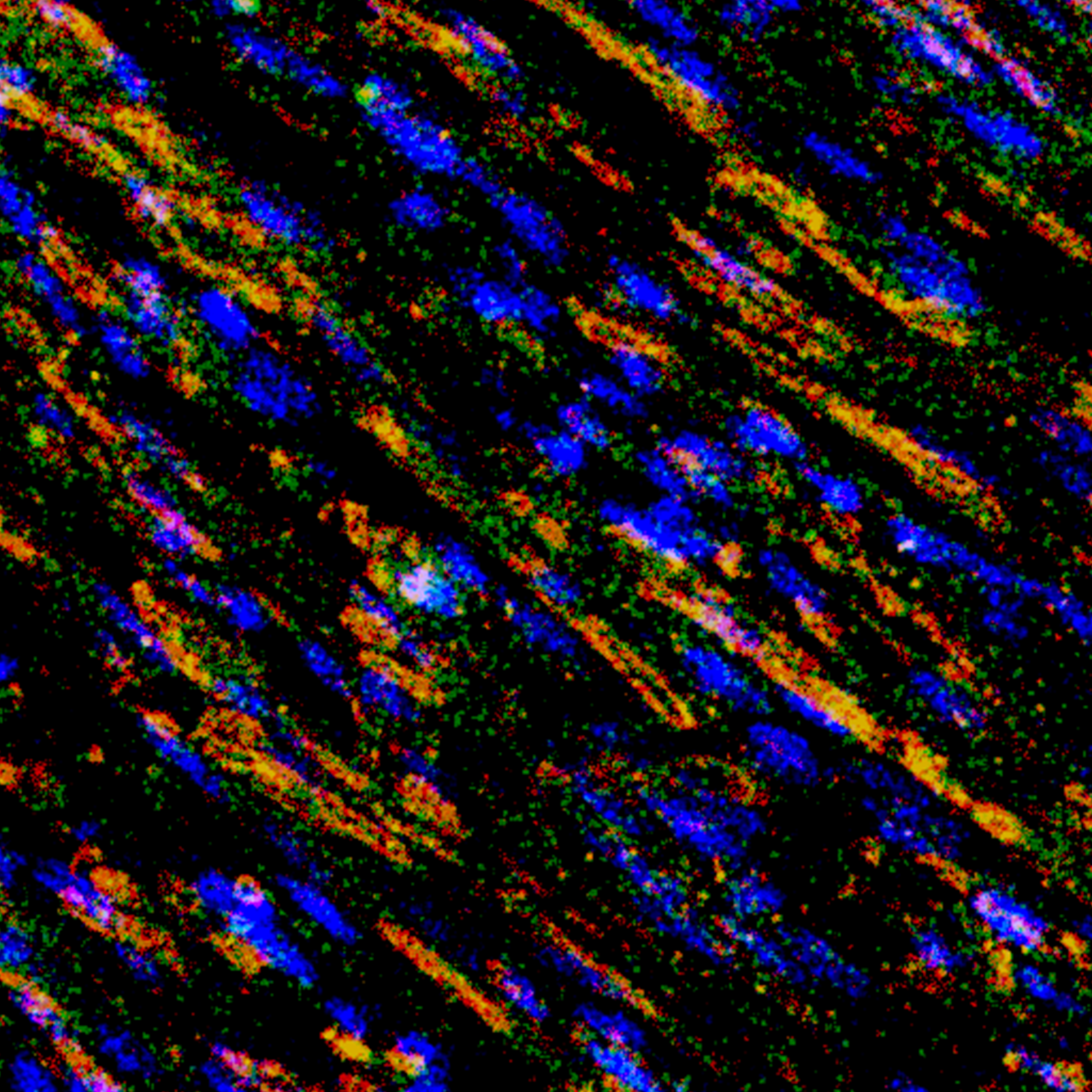

Supplement: Supplementary file 1 [file DataSheet3.ZIP › figure 2C P-bFGF (3).tiff]

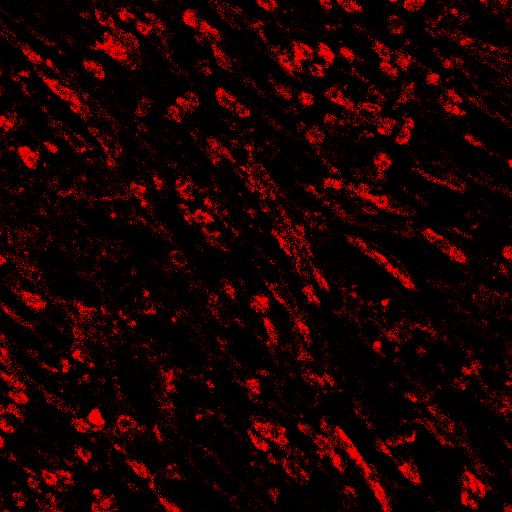

Supplement: Supplementary file 1 [file DataSheet3.ZIP › figure 2C FNI (1).tiff]

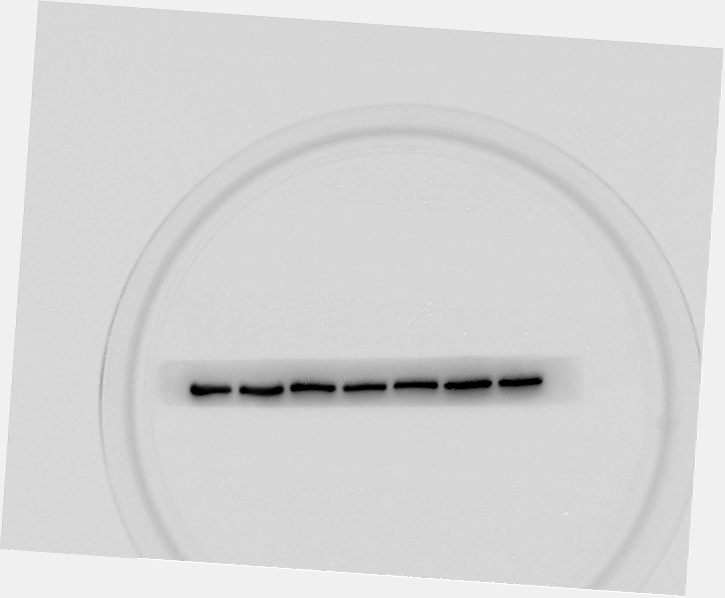

Supplement: Supplementary file 2 [file DataSheet8.ZIP › Figure5/H-figure 5 GAPDH.tif]

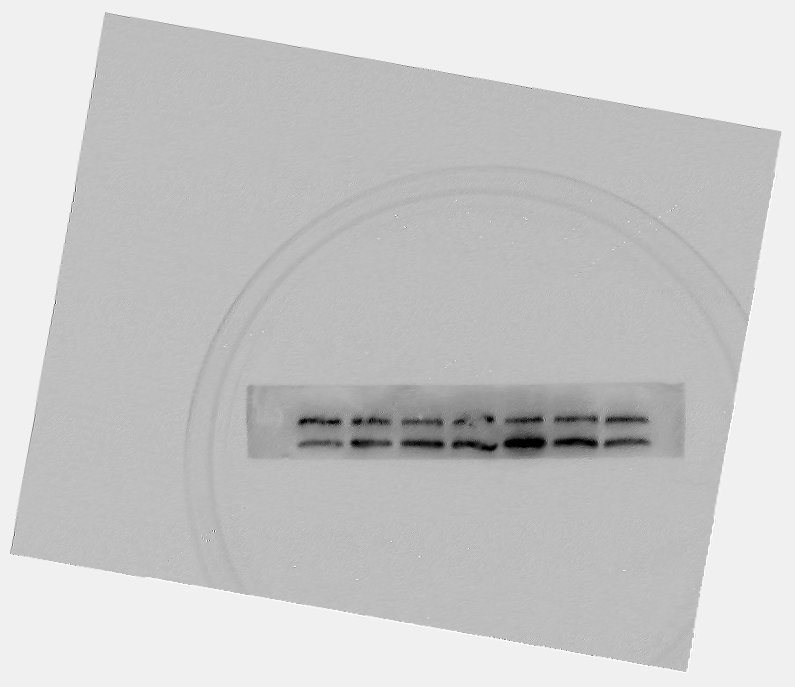

Supplement: Supplementary file 2 [file DataSheet8.ZIP › Figure5/H-figure 5 LC3.tif]

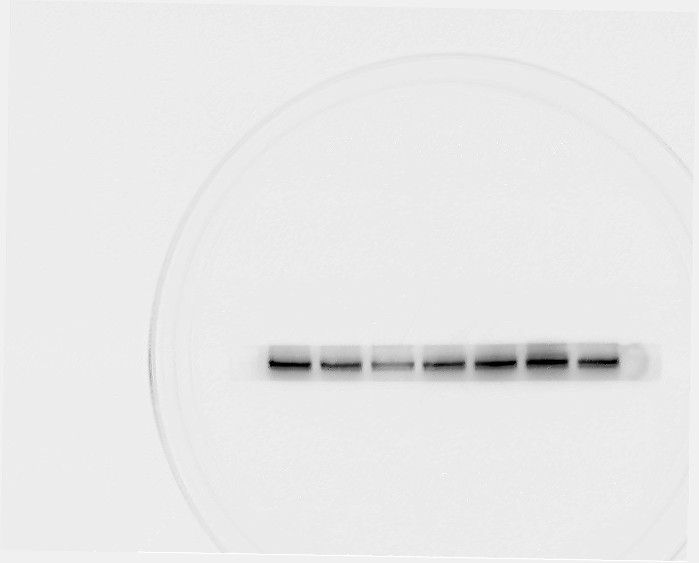

Supplement: Supplementary file 2 [file DataSheet8.ZIP › Figure5/H-figure 5 p62.tif]

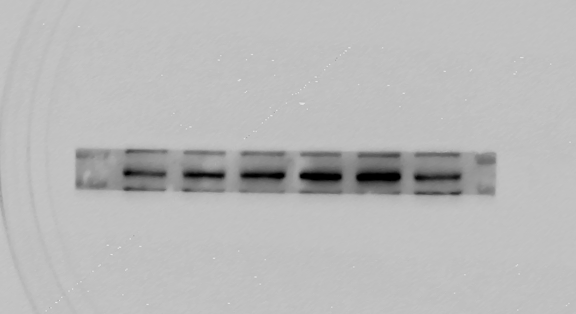

Supplement: Supplementary file 2 [file DataSheet8.ZIP › Figure5/figure 5 ATG5.tif]

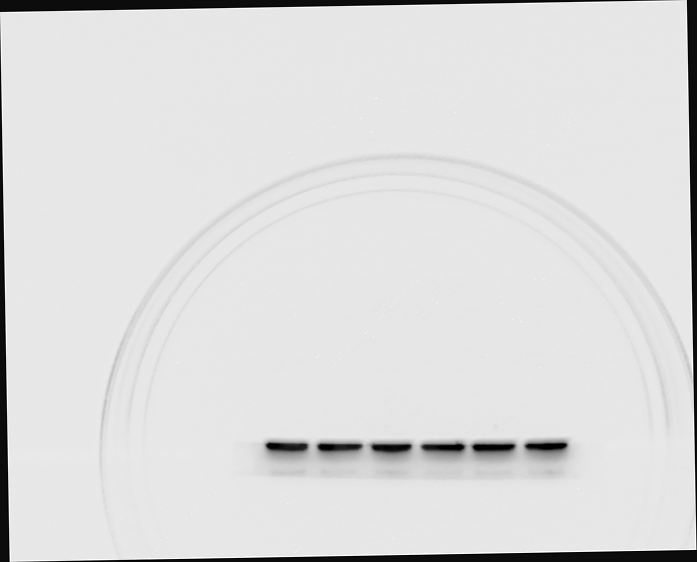

Supplement: Supplementary file 2 [file DataSheet8.ZIP › Figure5/figure 5 GADPH.tif]

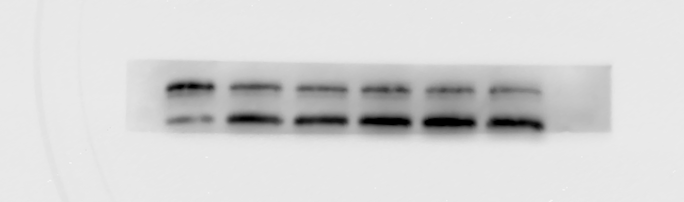

Supplement: Supplementary file 2 [file DataSheet8.ZIP › Figure5/figure 5 LC3 .tif]

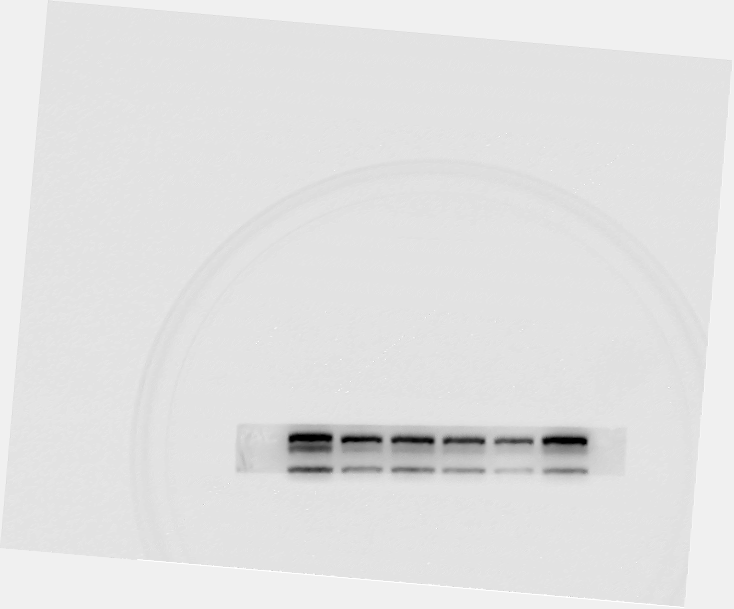

Supplement: Supplementary file 2 [file DataSheet8.ZIP › Figure5/figure 5 PAK1.tif]

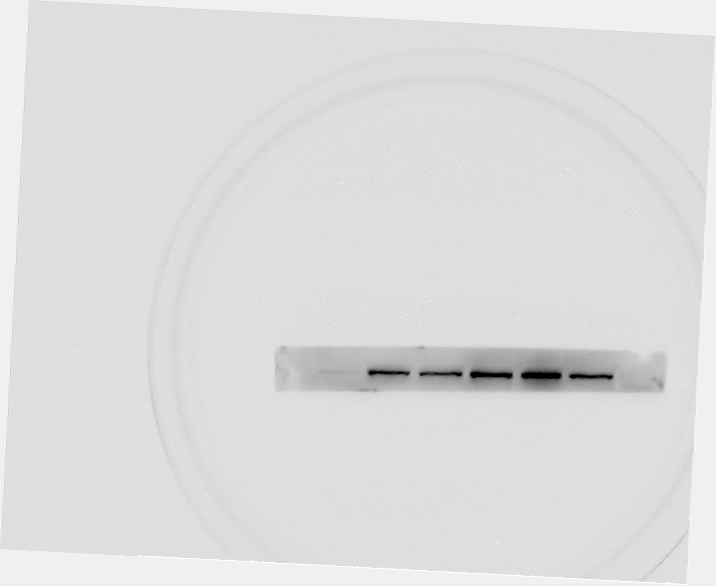

Supplement: Supplementary file 2 [file DataSheet8.ZIP › Figure5/figure 5 PPAK1.tif]

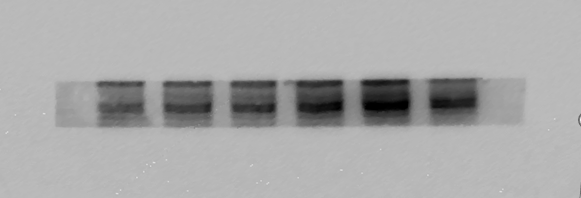

Supplement: Supplementary file 2 [file DataSheet8.ZIP › Figure5/figure 5 beclin1 .tif]

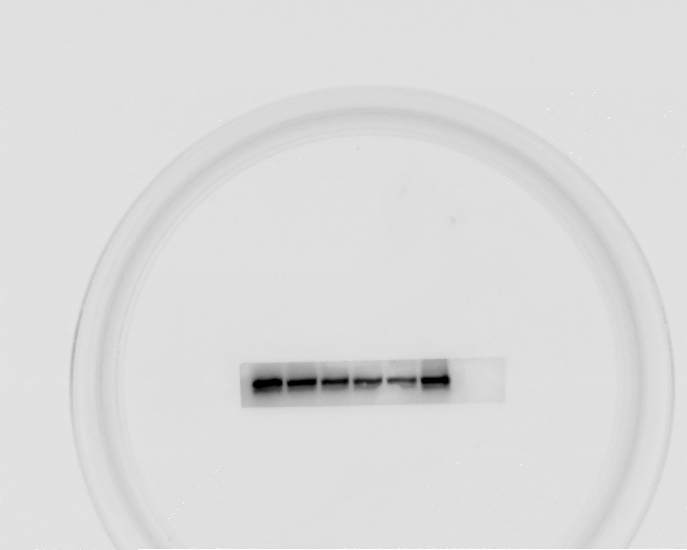

Supplement: Supplementary file 2 [file DataSheet8.ZIP › Figure5/figure 5 p62.tif]

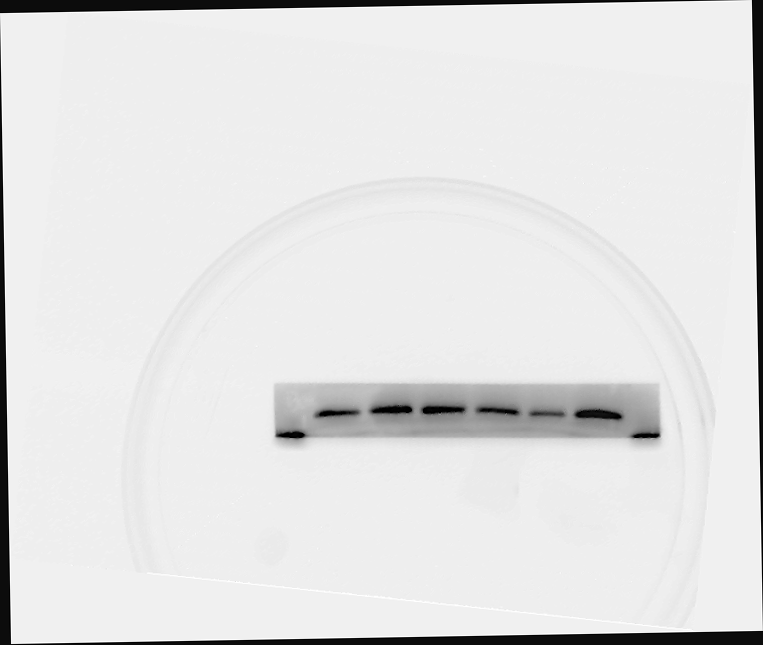

Supplement: Supplementary file 3 [file DataSheet9.ZIP › Figure6/figure 6 BAX.tif]

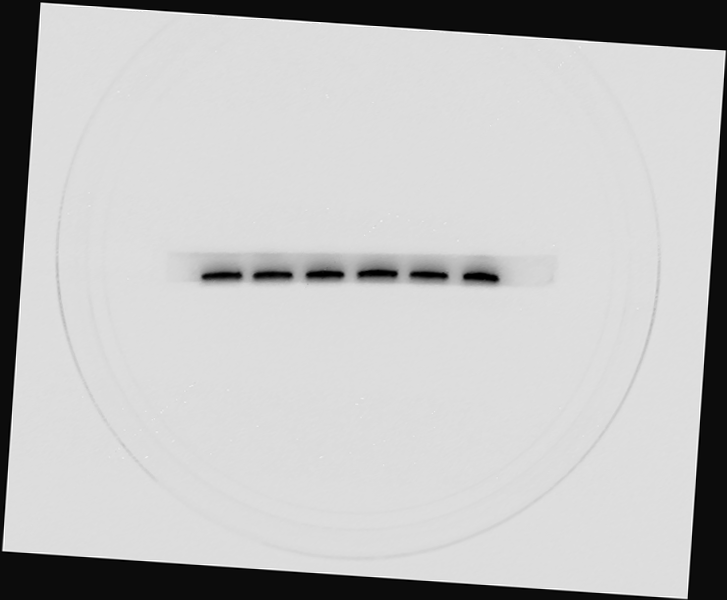

Supplement: Supplementary file 3 [file DataSheet9.ZIP › Figure6/figure 6 GADPH.tif]

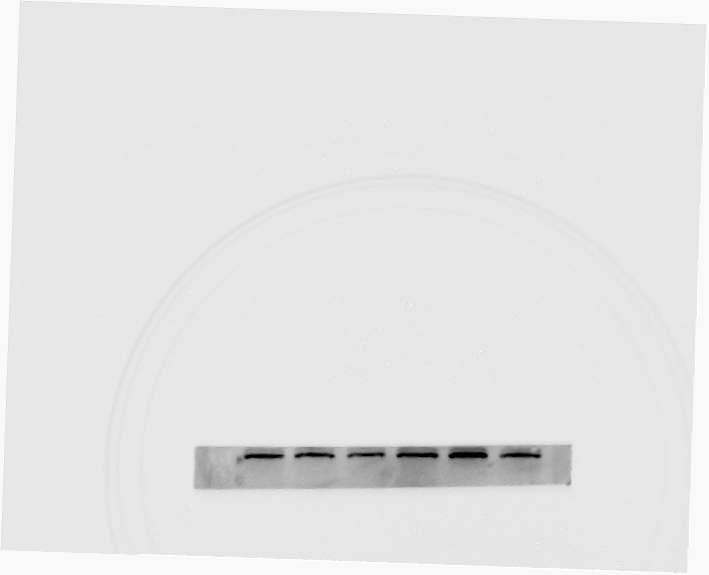

Supplement: Supplementary file 3 [file DataSheet9.ZIP › Figure6/figure 6 bcl2.tif]

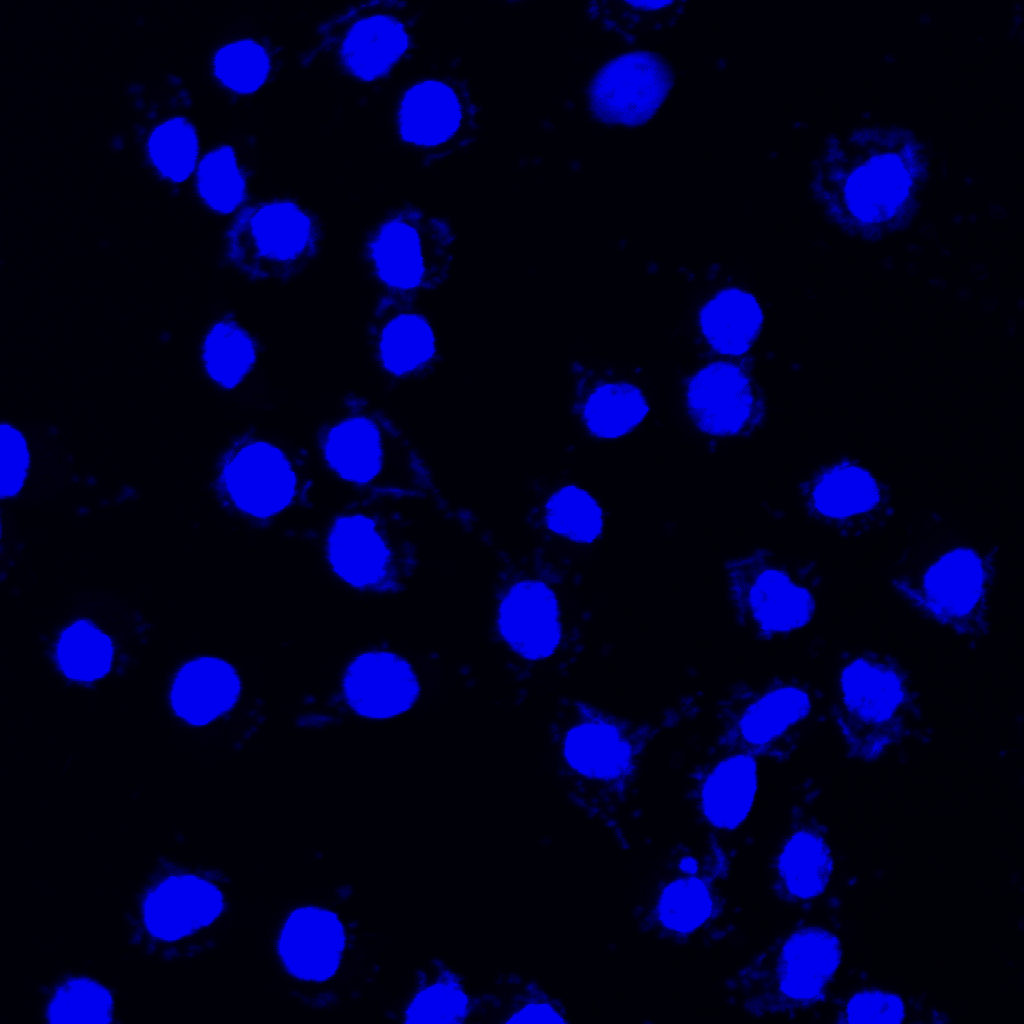

Supplement: Supplementary file 3 [file DataSheet9.ZIP › Figure6/figure 6A H2O2 (1).tif]

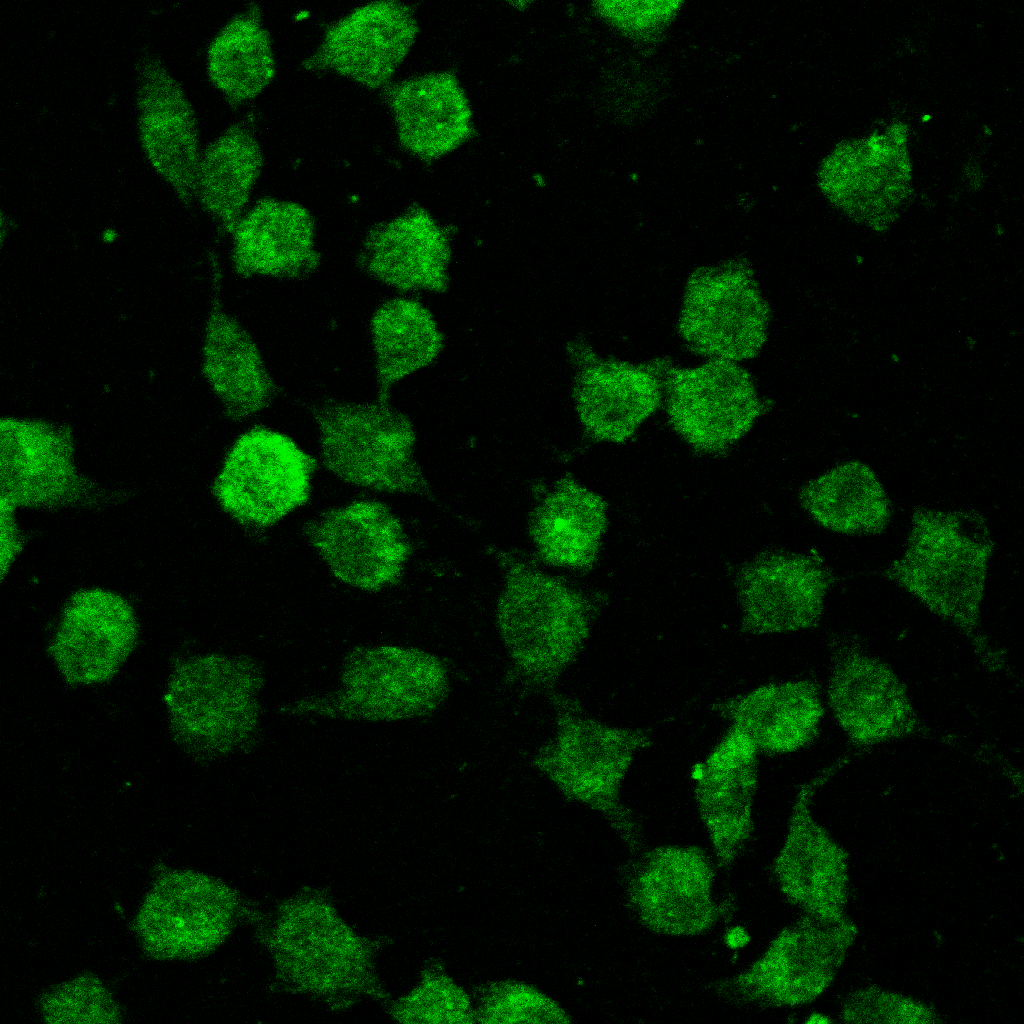

Supplement: Supplementary file 3 [file DataSheet9.ZIP › Figure6/figure 6A H2O2 (2).tif]

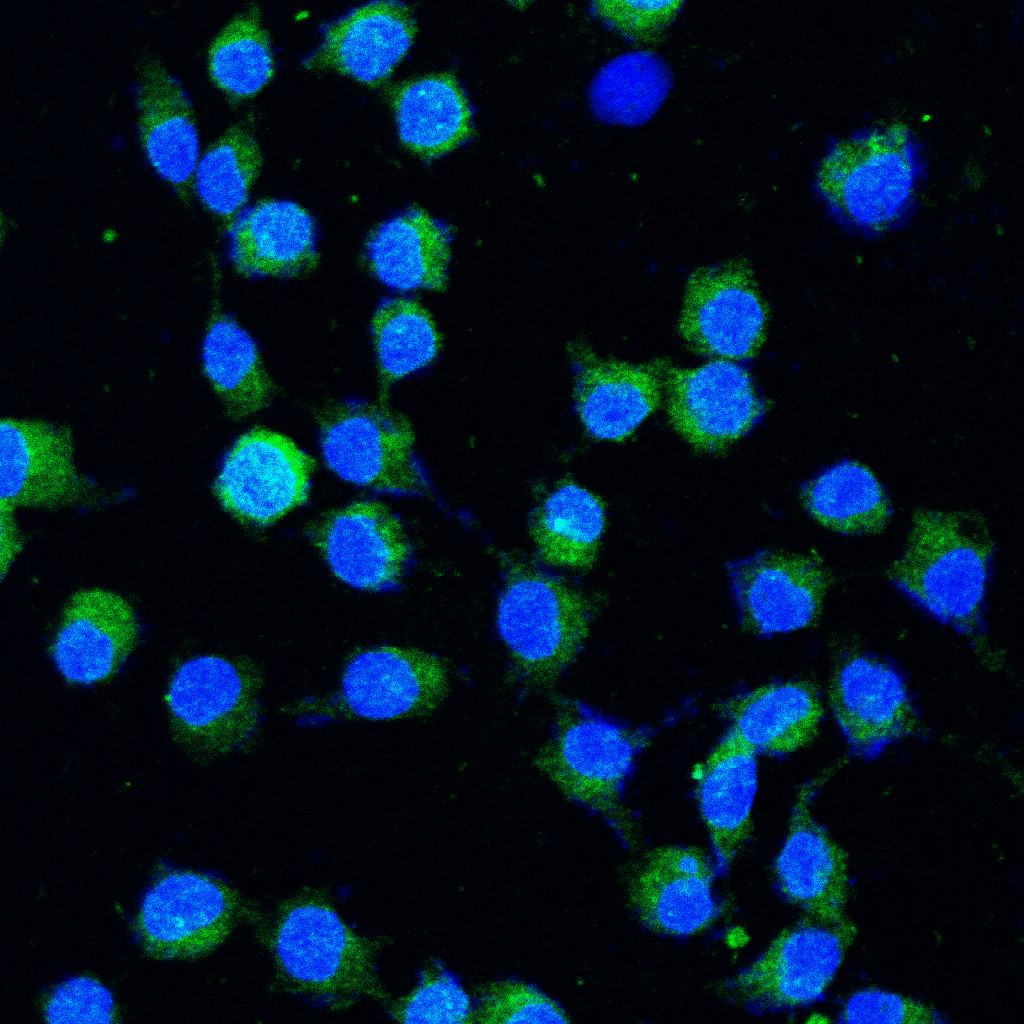

Supplement: Supplementary file 3 [file DataSheet9.ZIP › Figure6/figure 6A H2O2 (3).tif]

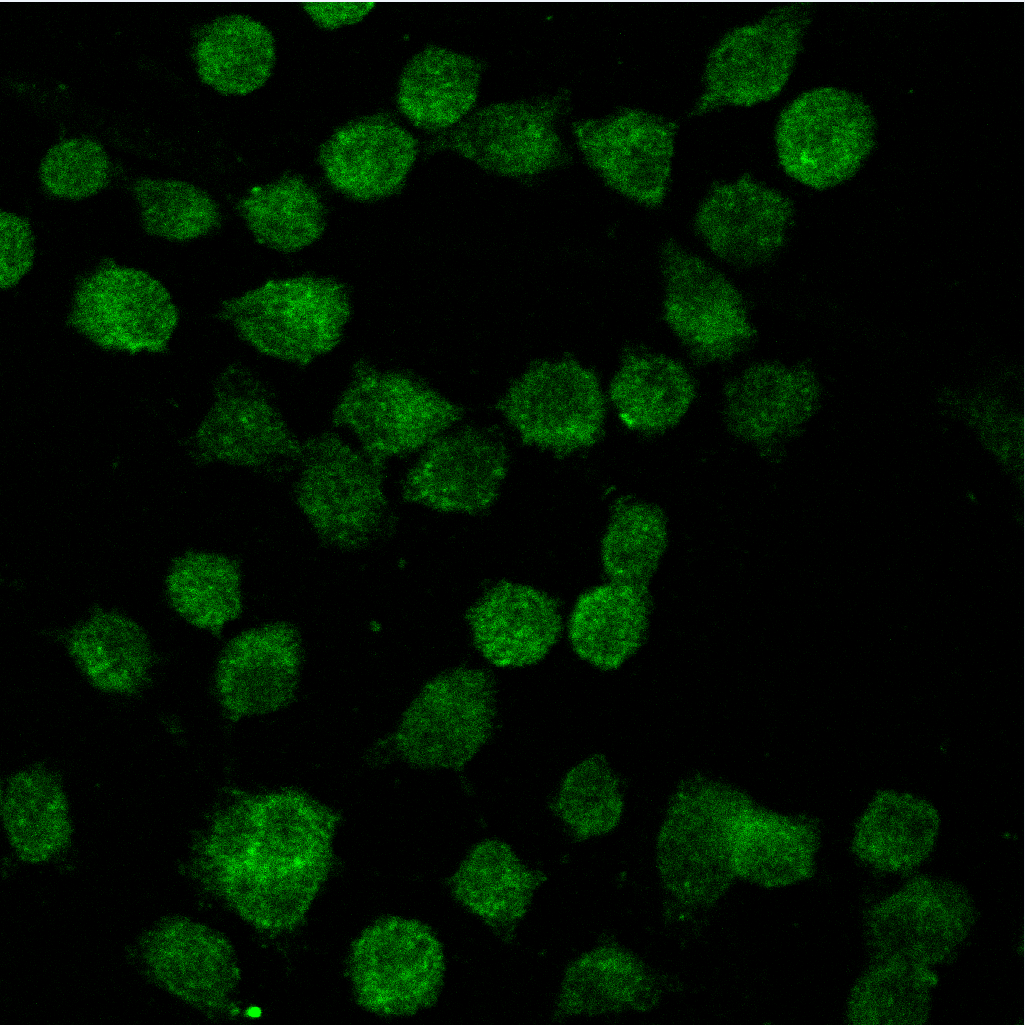

Supplement: Supplementary file 3 [file DataSheet9.ZIP › Figure6/figure 6A IPA-3 (1).png]

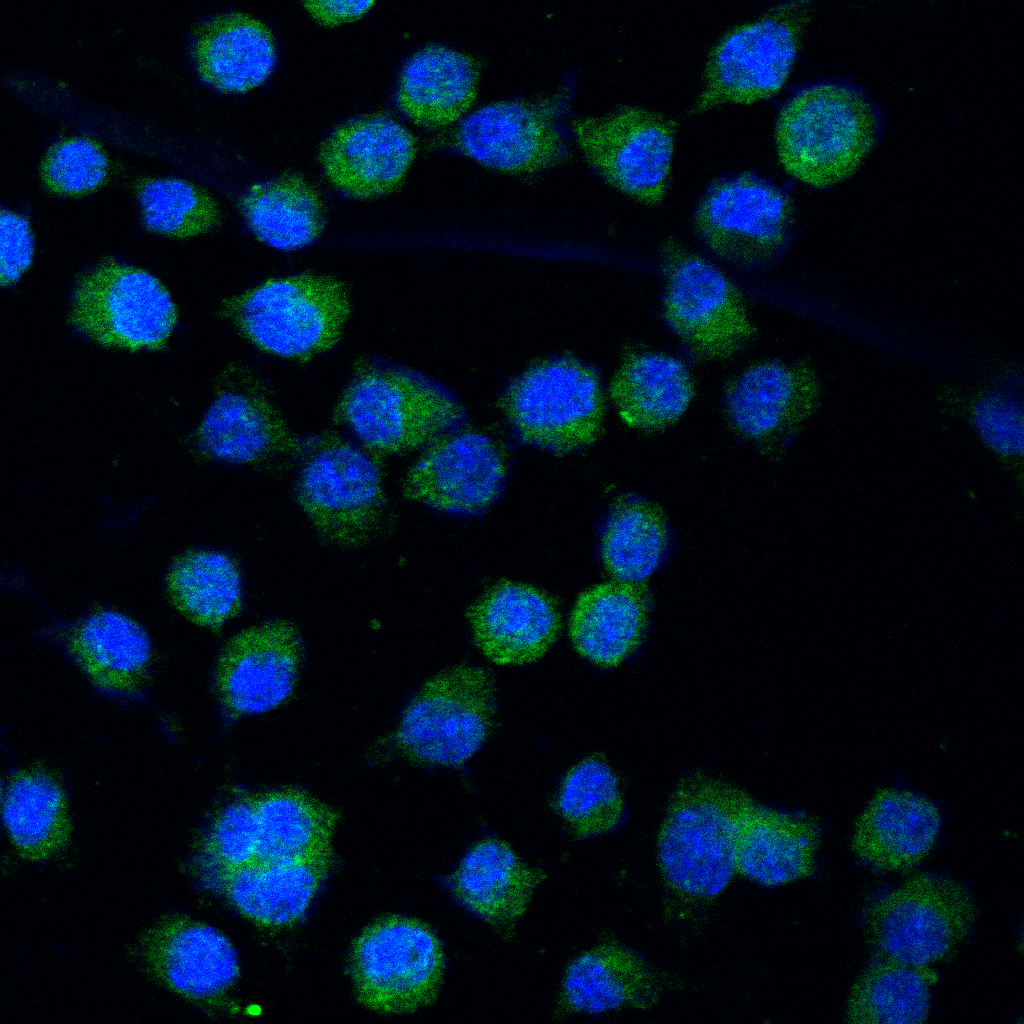

Supplement: Supplementary file 3 [file DataSheet9.ZIP › Figure6/figure 6A IPA-3 (1).tif]

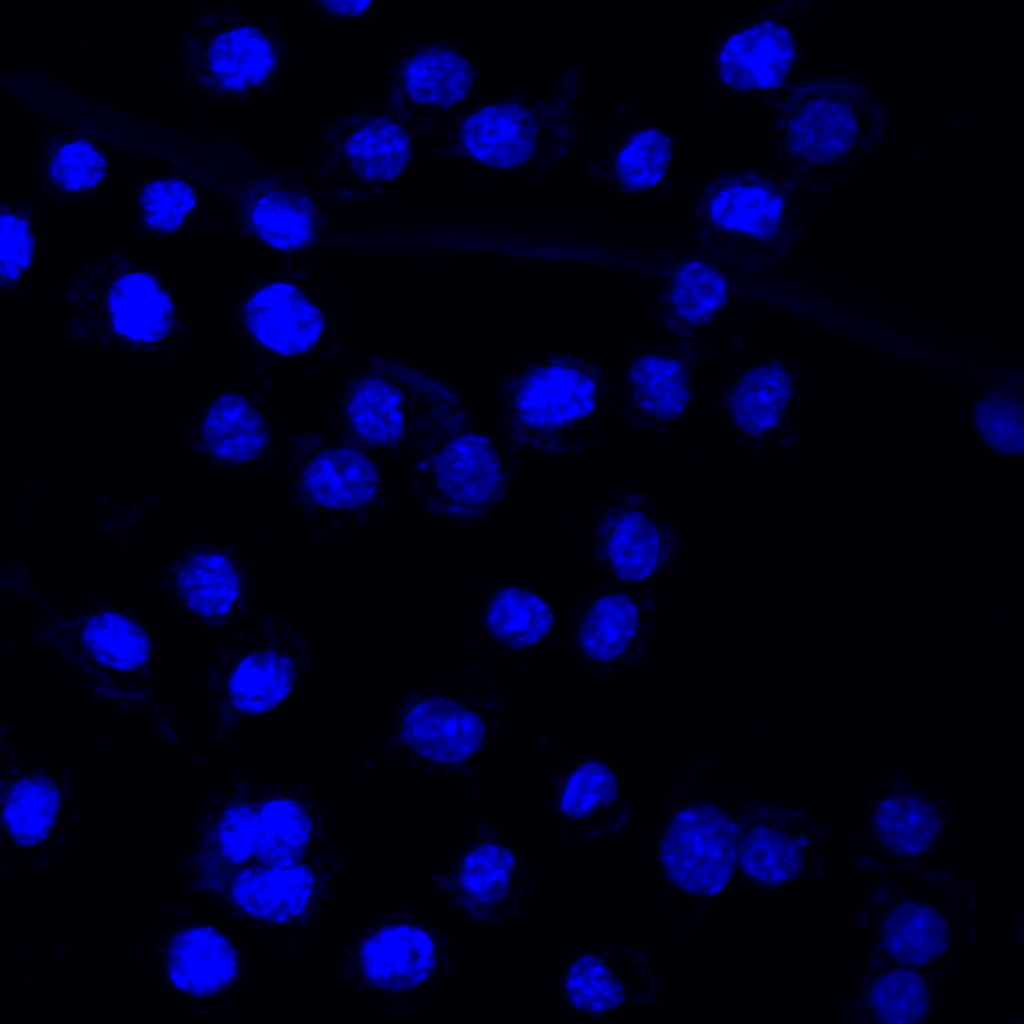

Supplement: Supplementary file 3 [file DataSheet9.ZIP › Figure6/figure 6A IPA-3 (2).tif]

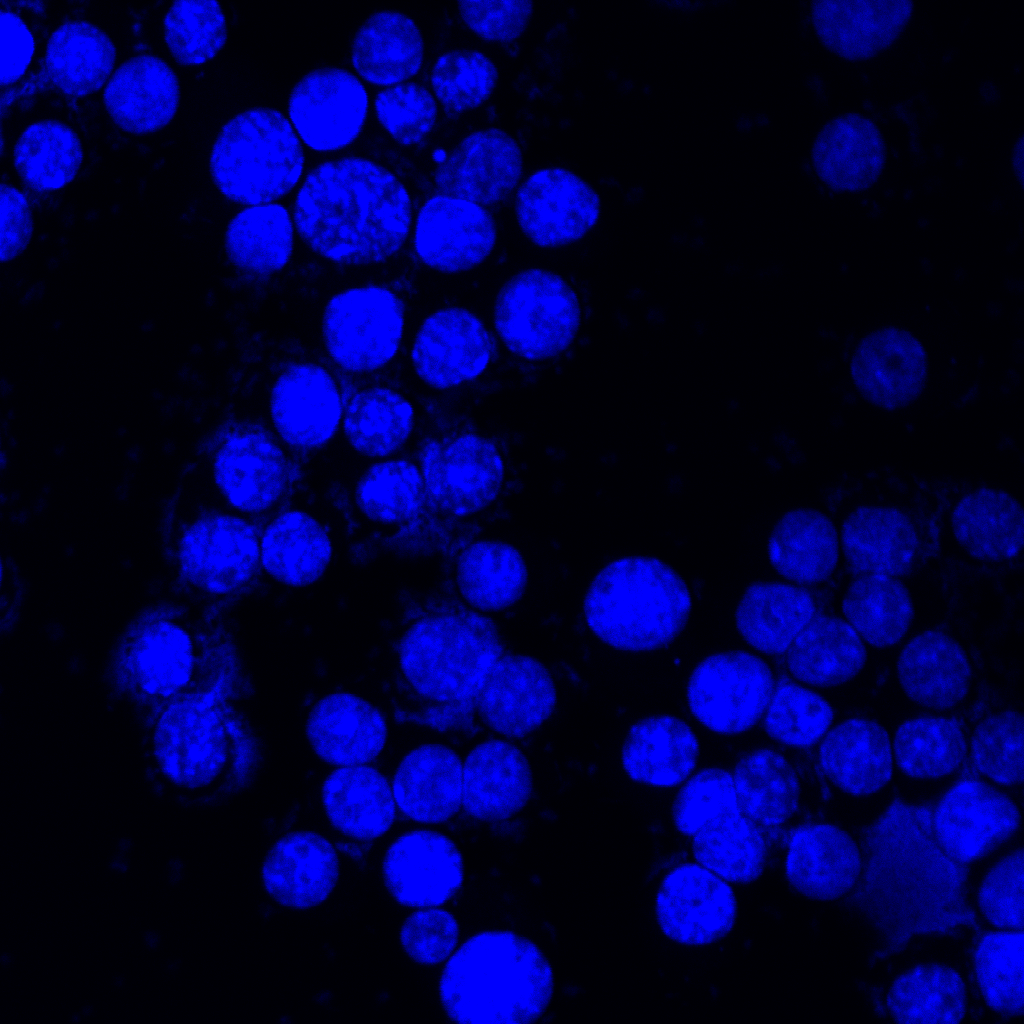

Supplement: Supplementary file 3 [file DataSheet9.ZIP › Figure6/figure 6A P-bFGF (1).tif]

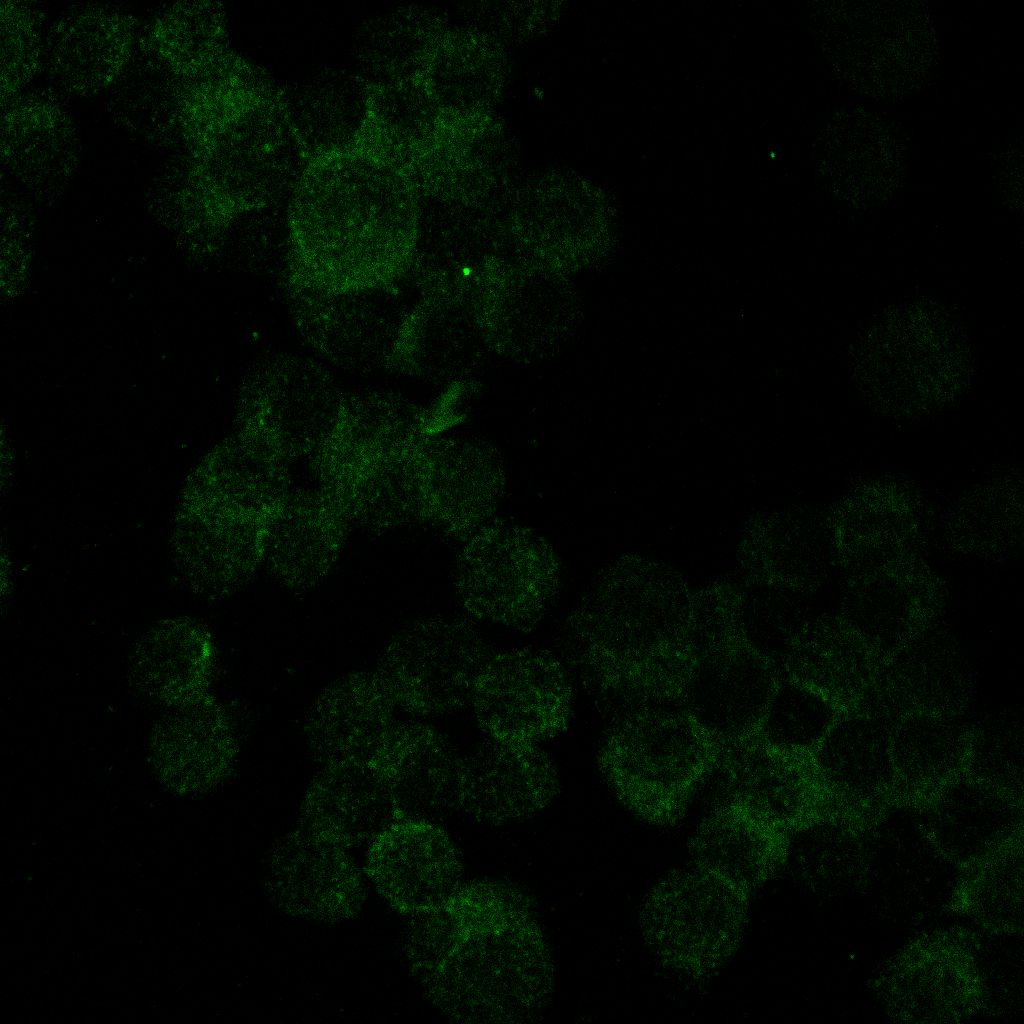

Supplement: Supplementary file 3 [file DataSheet9.ZIP › Figure6/figure 6A P-bFGF (2).tif]

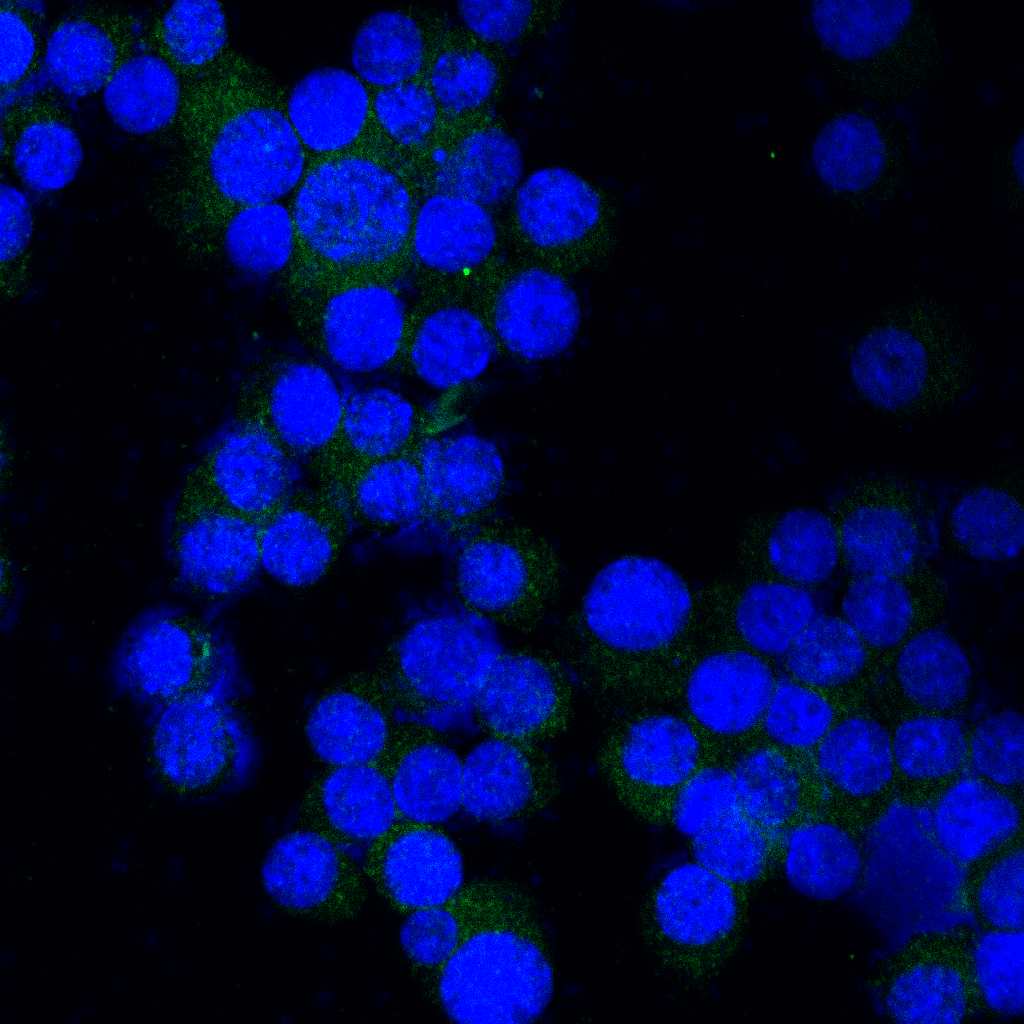

Supplement: Supplementary file 3 [file DataSheet9.ZIP › Figure6/figure 6A P-bFGF (3).tif]

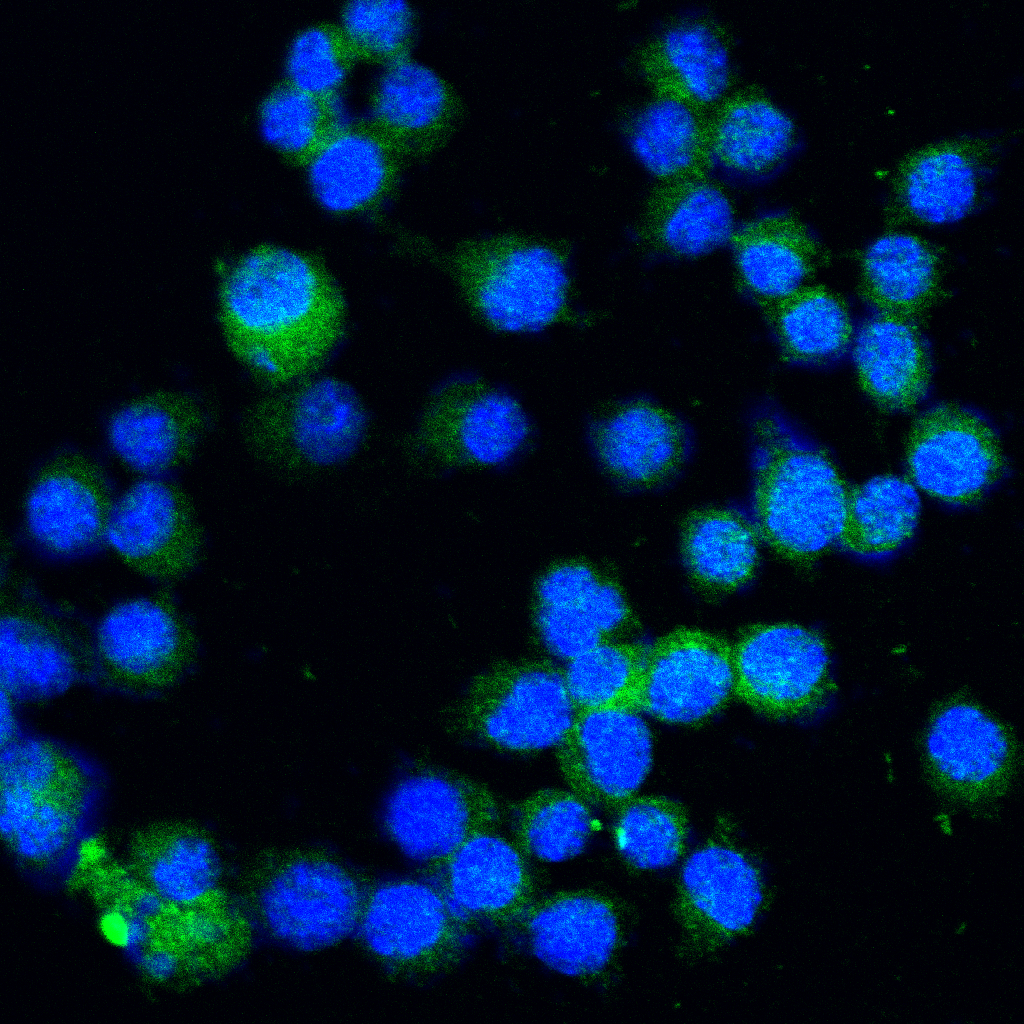

Supplement: Supplementary file 3 [file DataSheet9.ZIP › Figure6/figure 6A Poloxamer (1).tif]

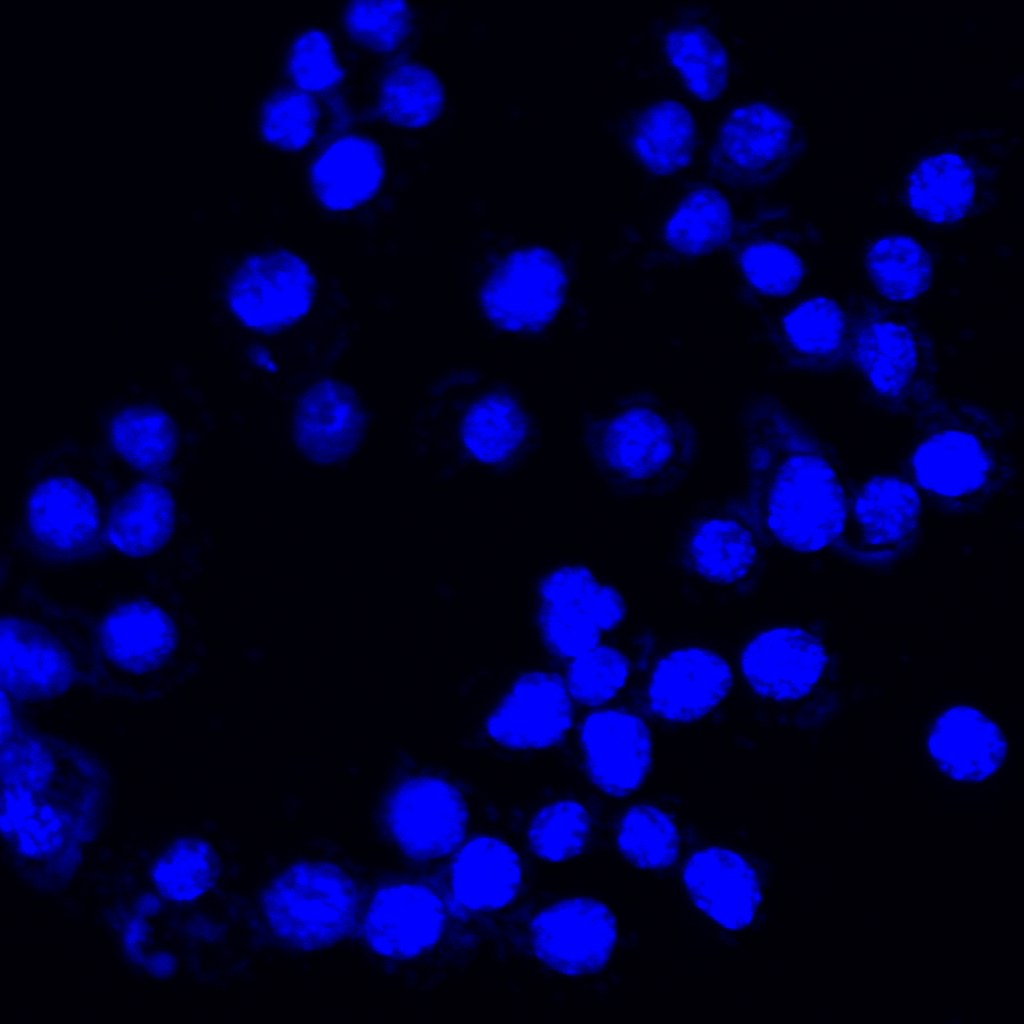

Supplement: Supplementary file 3 [file DataSheet9.ZIP › Figure6/figure 6A Poloxamer (2).tif]

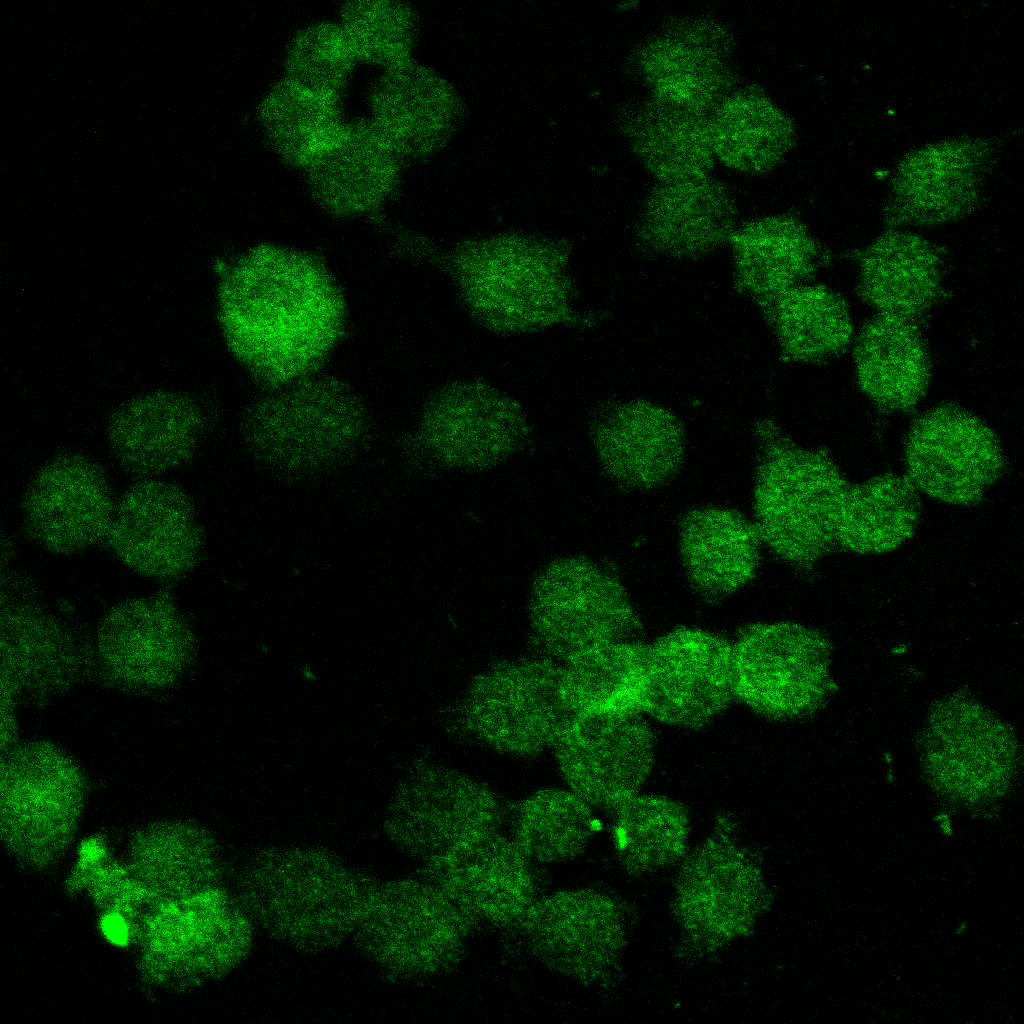

Supplement: Supplementary file 3 [file DataSheet9.ZIP › Figure6/figure 6A Poloxamer (3).tif]

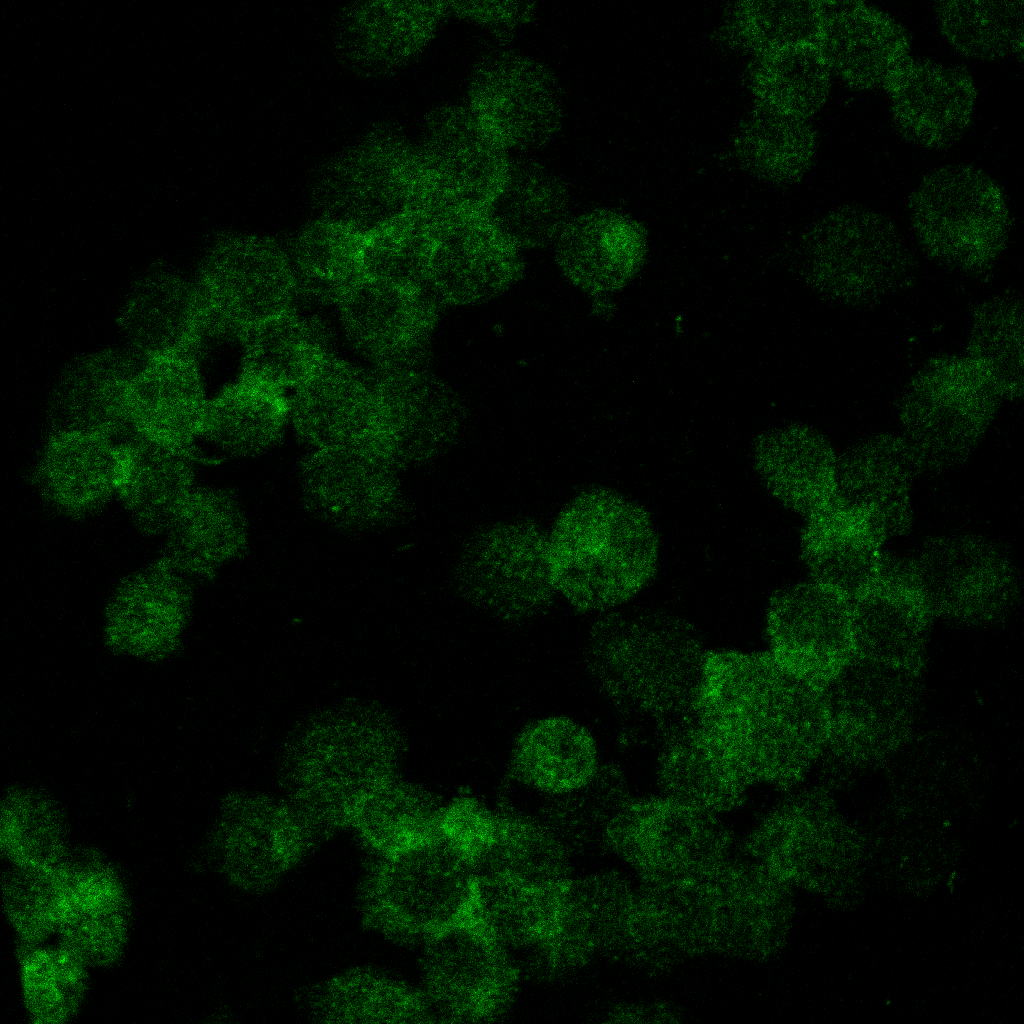

Supplement: Supplementary file 3 [file DataSheet9.ZIP › Figure6/figure 6A bFGF (1).tif]

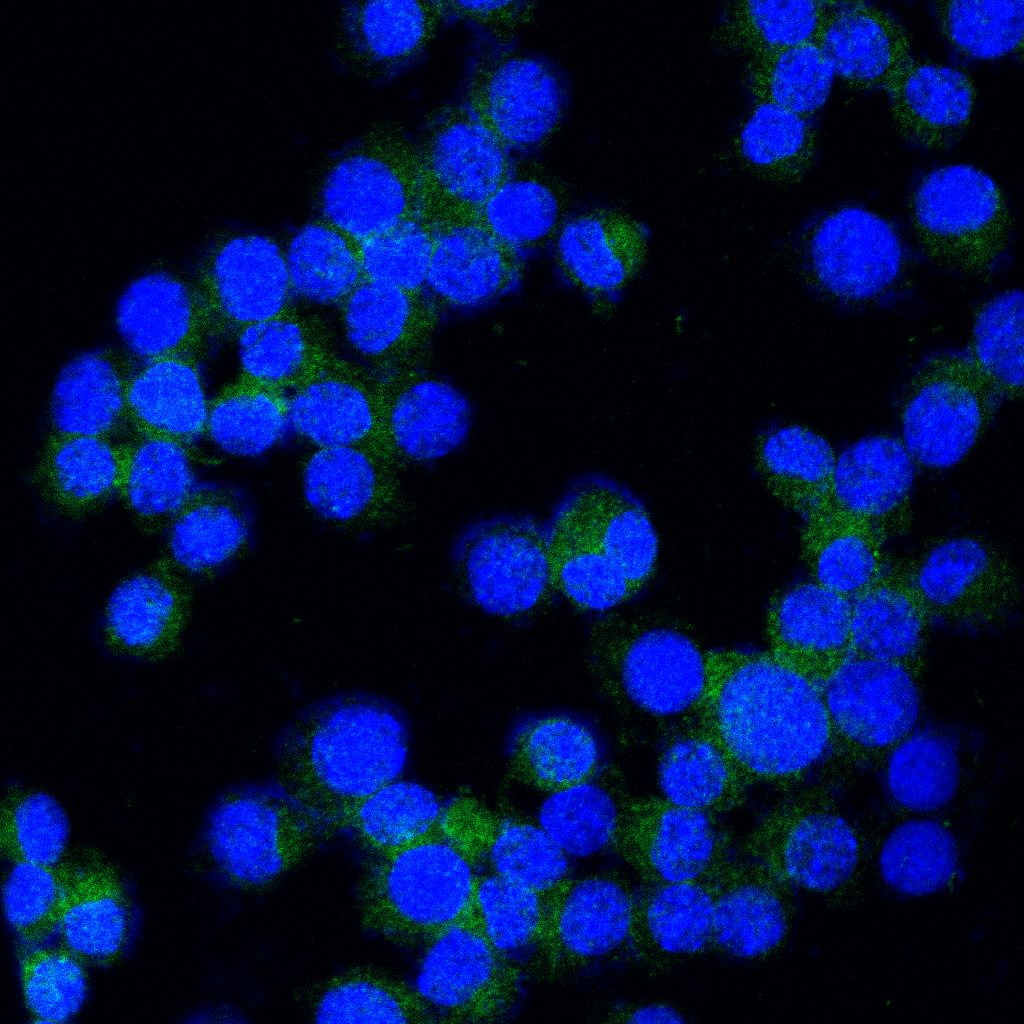

Supplement: Supplementary file 3 [file DataSheet9.ZIP › Figure6/figure 6A bFGF (2).tif]

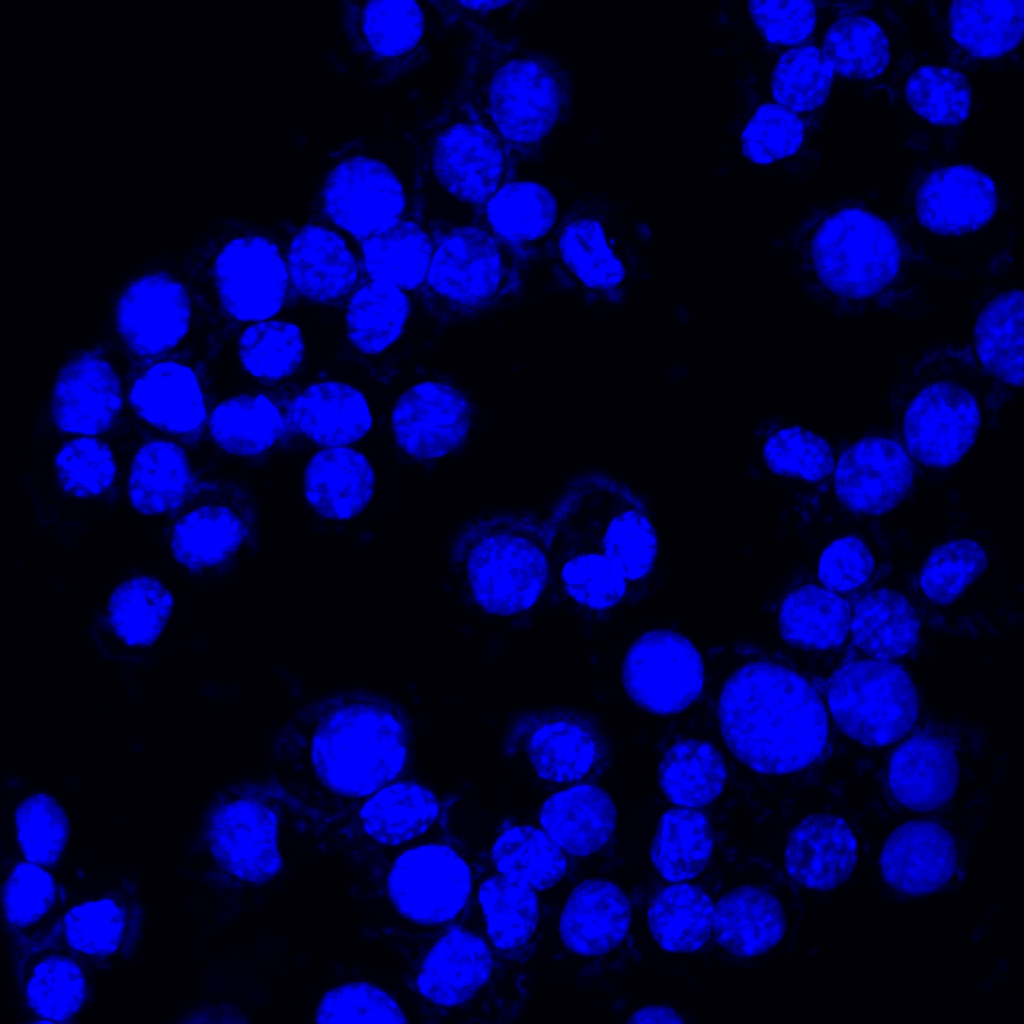

Supplement: Supplementary file 3 [file DataSheet9.ZIP › Figure6/figure 6A bFGF (3).tif]

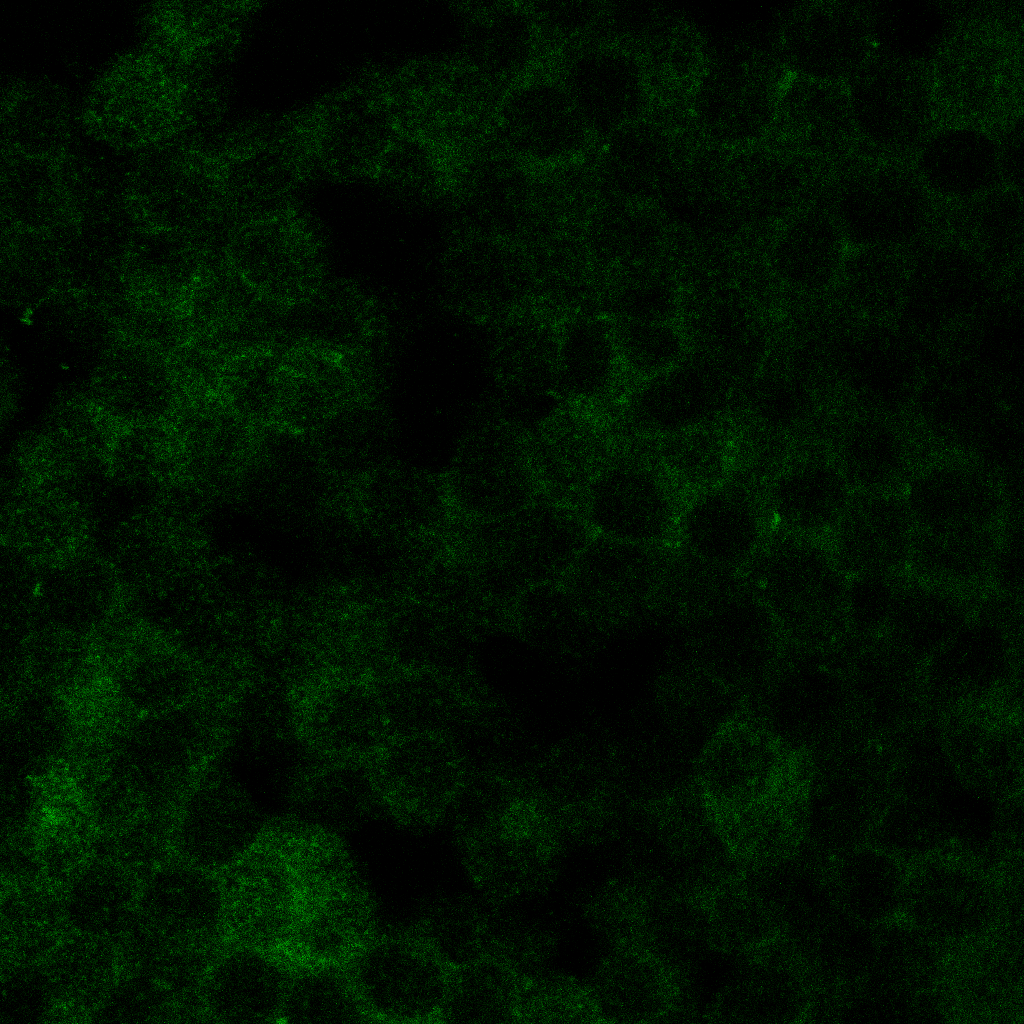

Supplement: Supplementary file 3 [file DataSheet9.ZIP › Figure6/figure 6A control (1).tif]

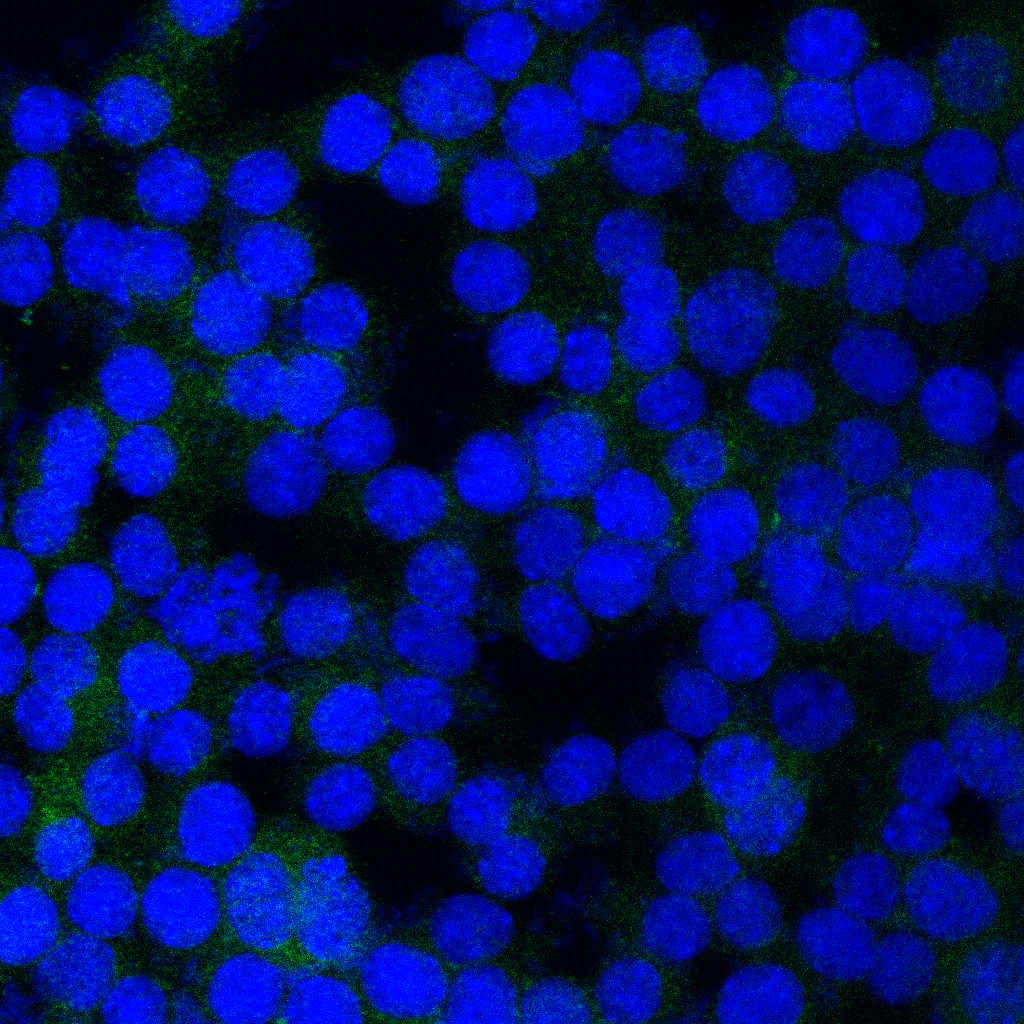

Supplement: Supplementary file 3 [file DataSheet9.ZIP › Figure6/figure 6A control (2).tif]

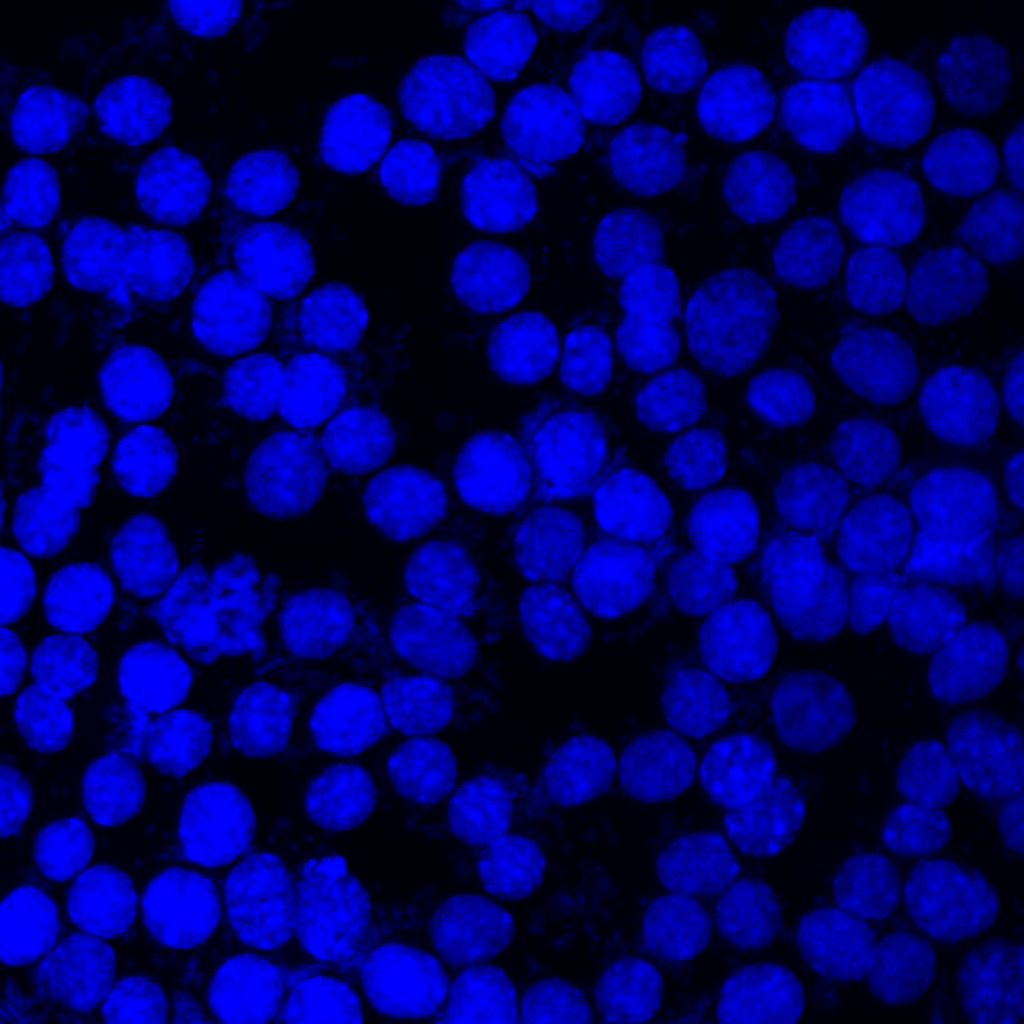

Supplement: Supplementary file 3 [file DataSheet9.ZIP › Figure6/figure 6A control (3).tif]

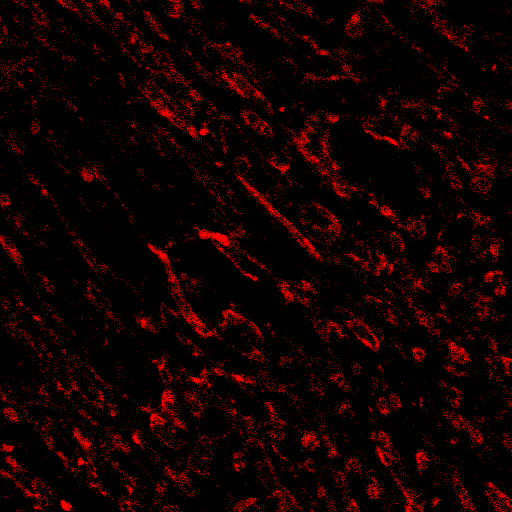

Supplement: Supplementary file 4 [file DataSheet4.ZIP › figure 2C Poloxamer (1).tiff]

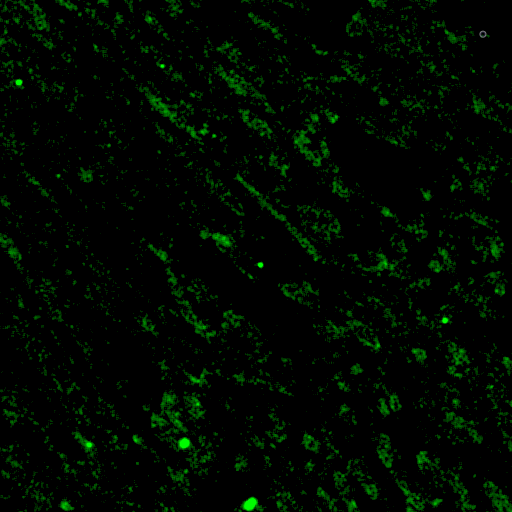

Supplement: Supplementary file 4 [file DataSheet4.ZIP › figure 2C Poloxamer (2).tiff]

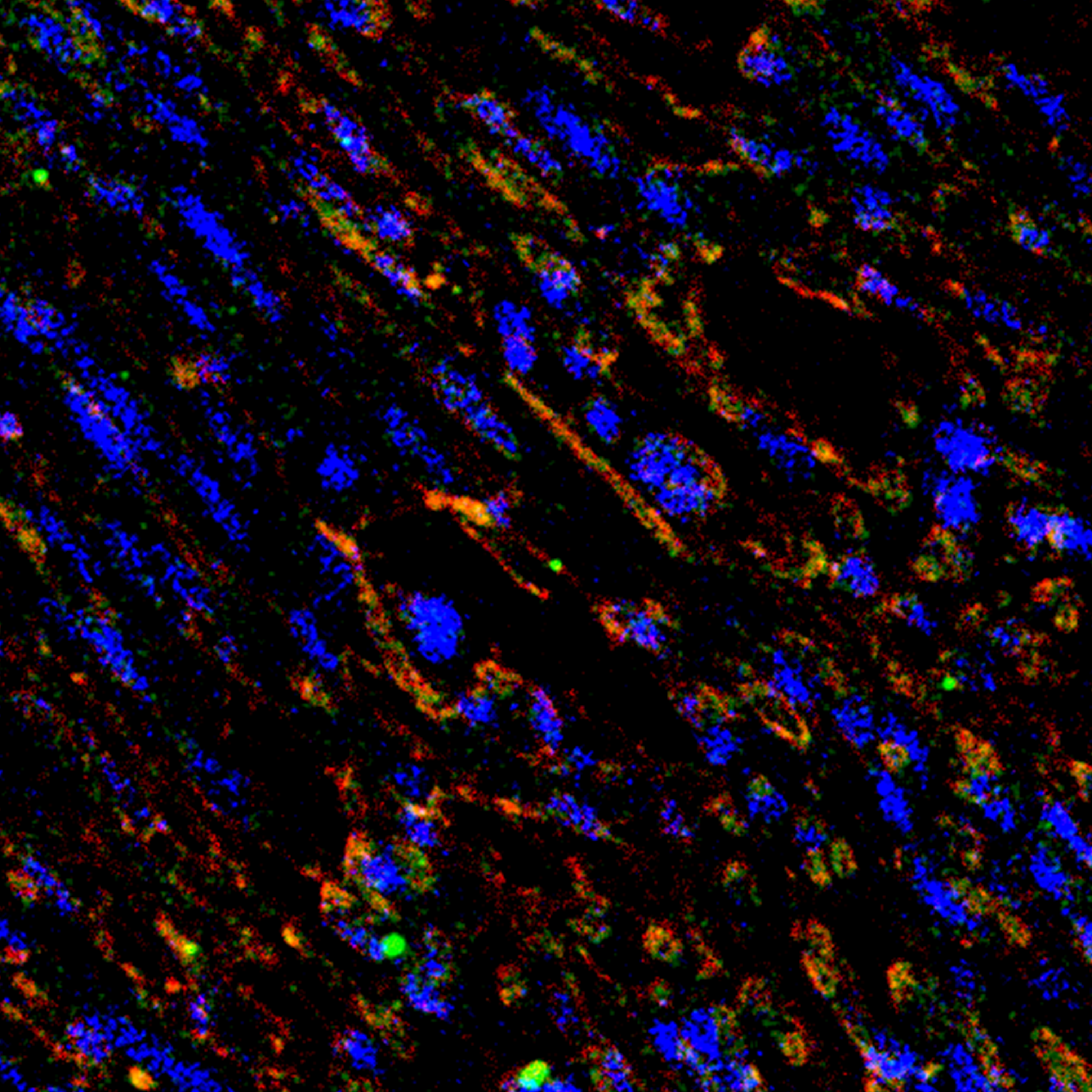

Supplement: Supplementary file 4 [file DataSheet4.ZIP › figure 2C Poloxamer (3).tiff]

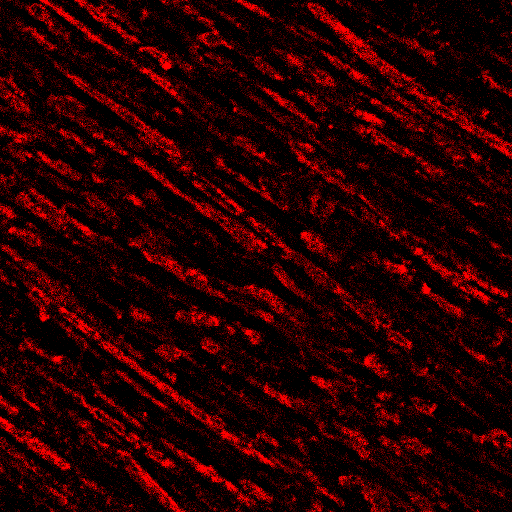

Supplement: Supplementary file 4 [file DataSheet4.ZIP › figure 2C sham (1).tiff]

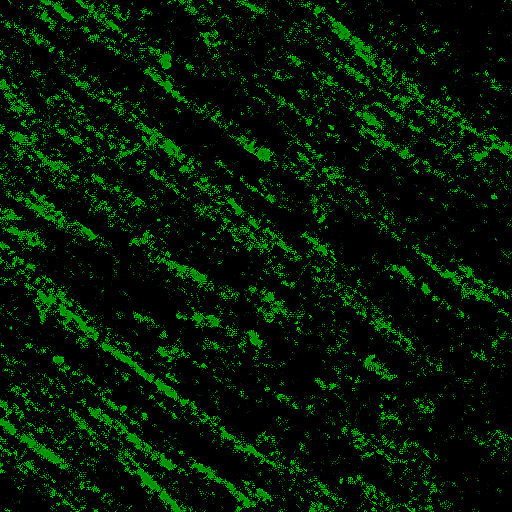

Supplement: Supplementary file 4 [file DataSheet4.ZIP › figure 2C sham (2).tiff]

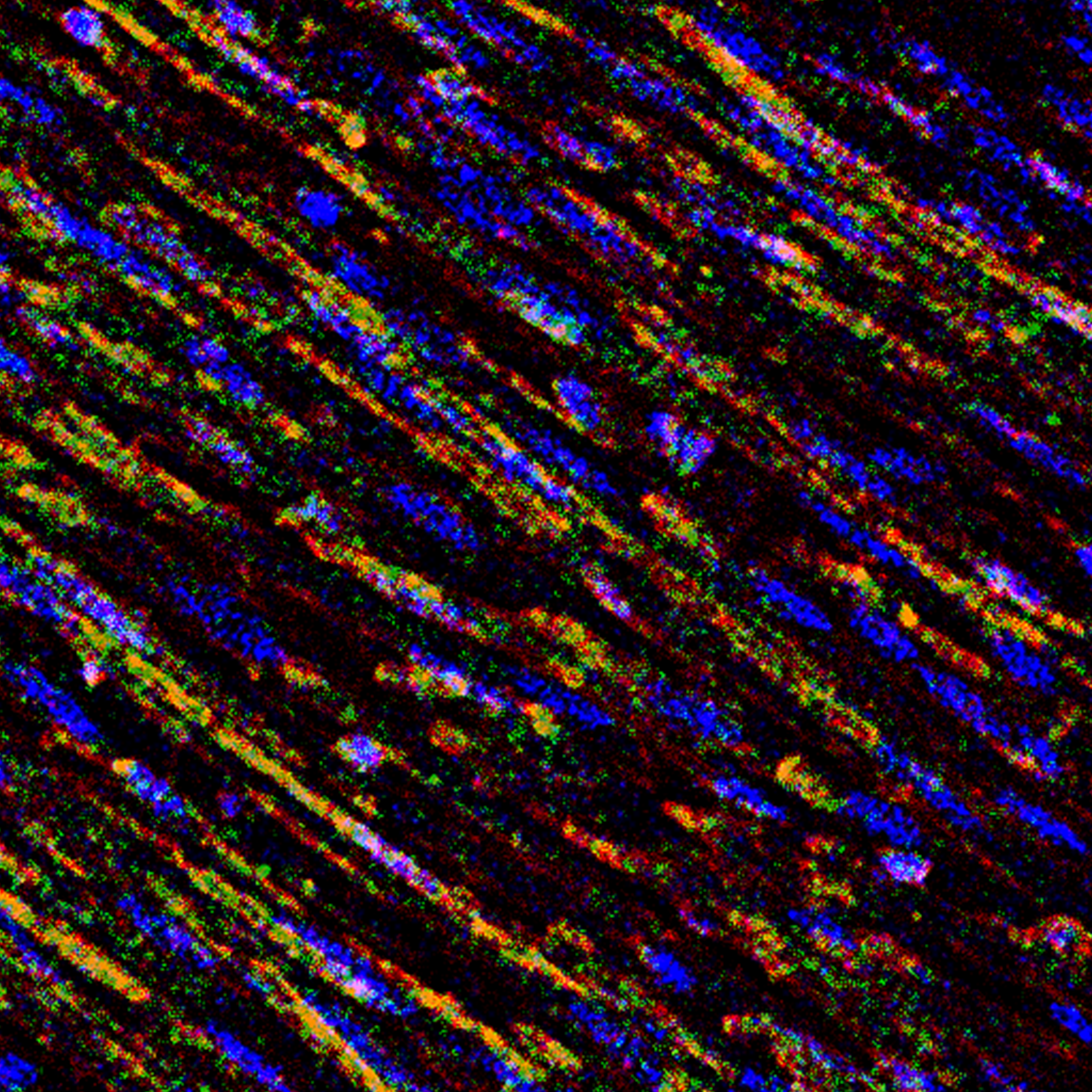

Supplement: Supplementary file 4 [file DataSheet4.ZIP › figure 2C sham (3).tiff]

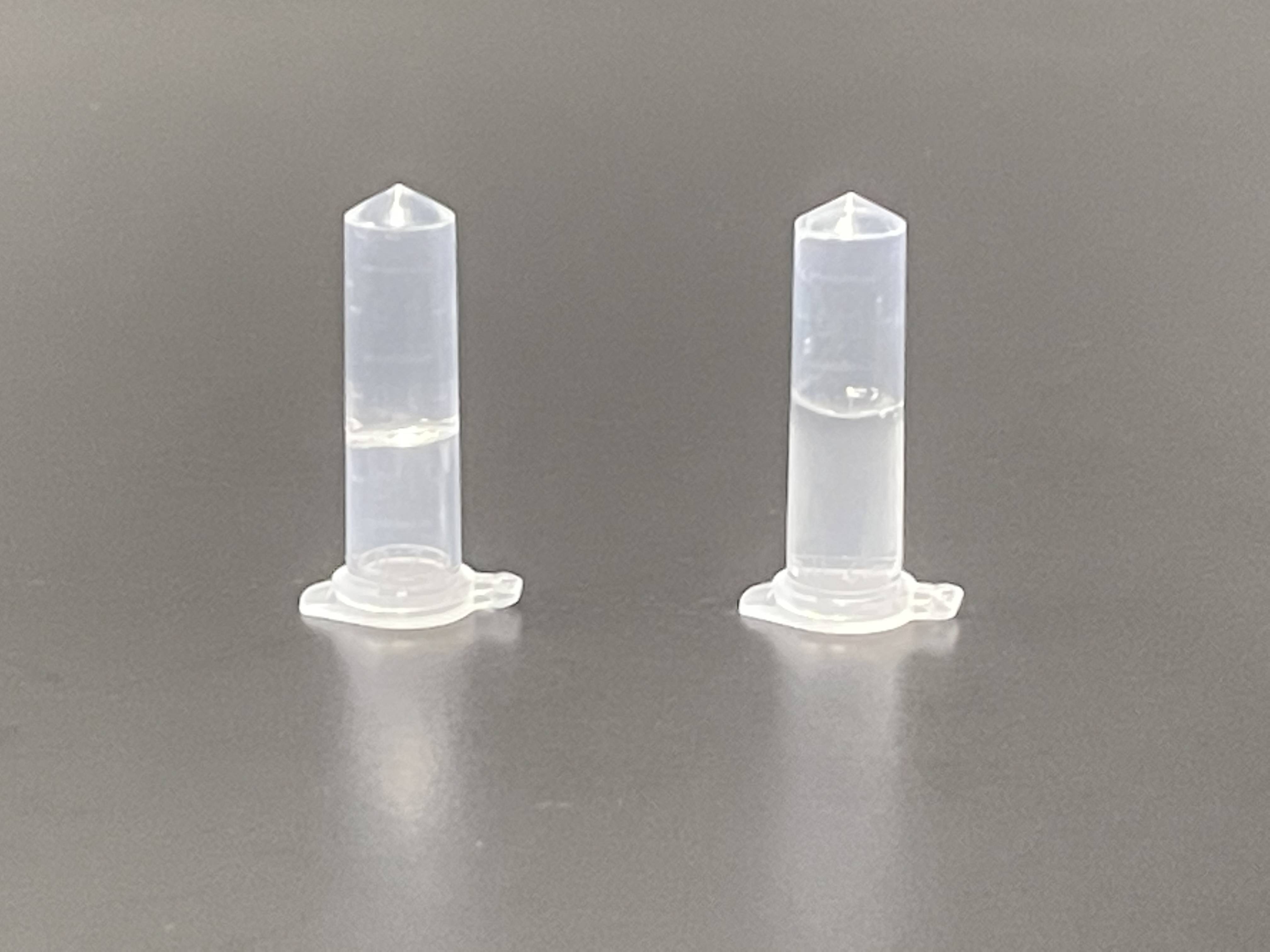

Supplement: Supplementary file 5 [file DataSheet1.ZIP › figure1/Figure 1A.jpg]

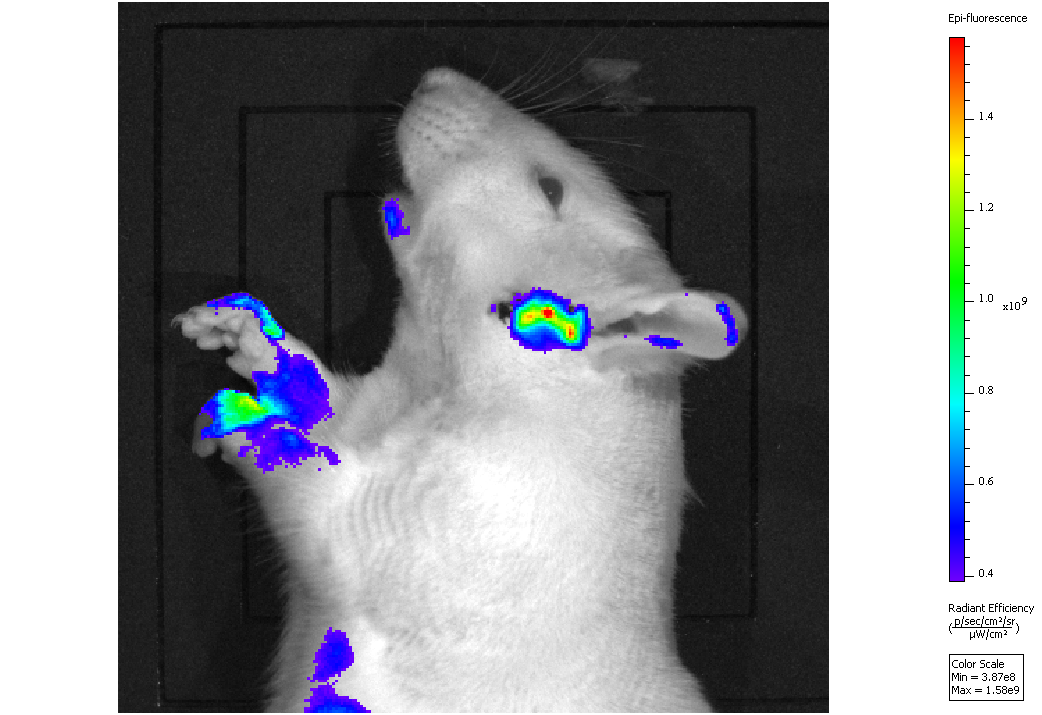

Supplement: Supplementary file 5 [file DataSheet1.ZIP › figure1/PbFGF-0h.tif]

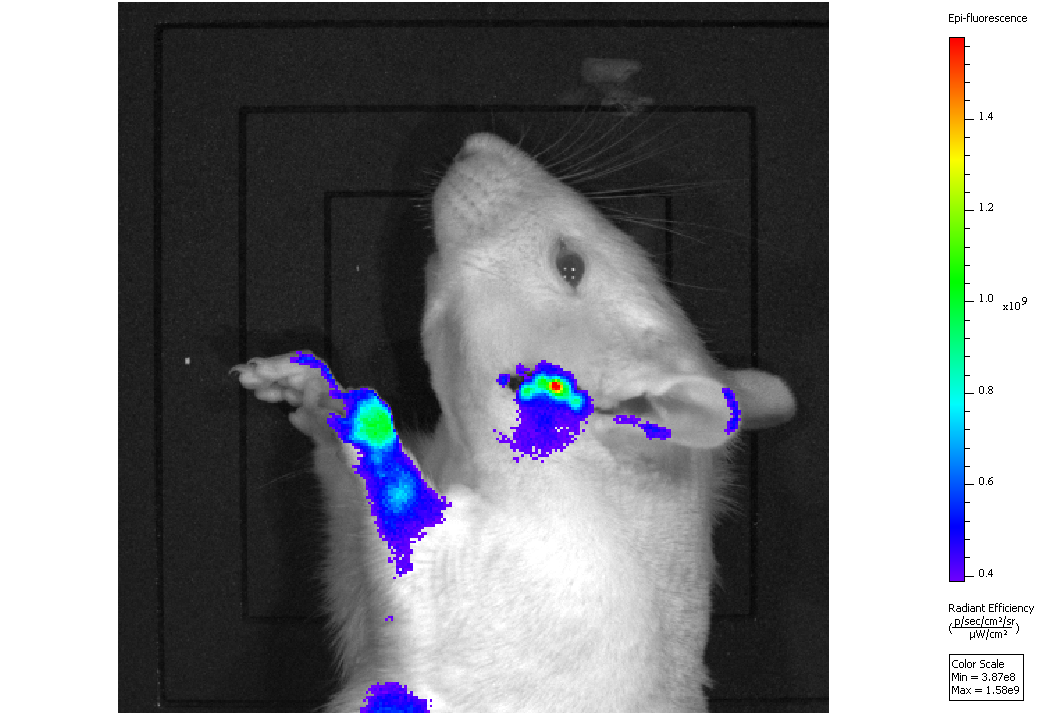

Supplement: Supplementary file 5 [file DataSheet1.ZIP › figure1/PbFGF-24h.tif]

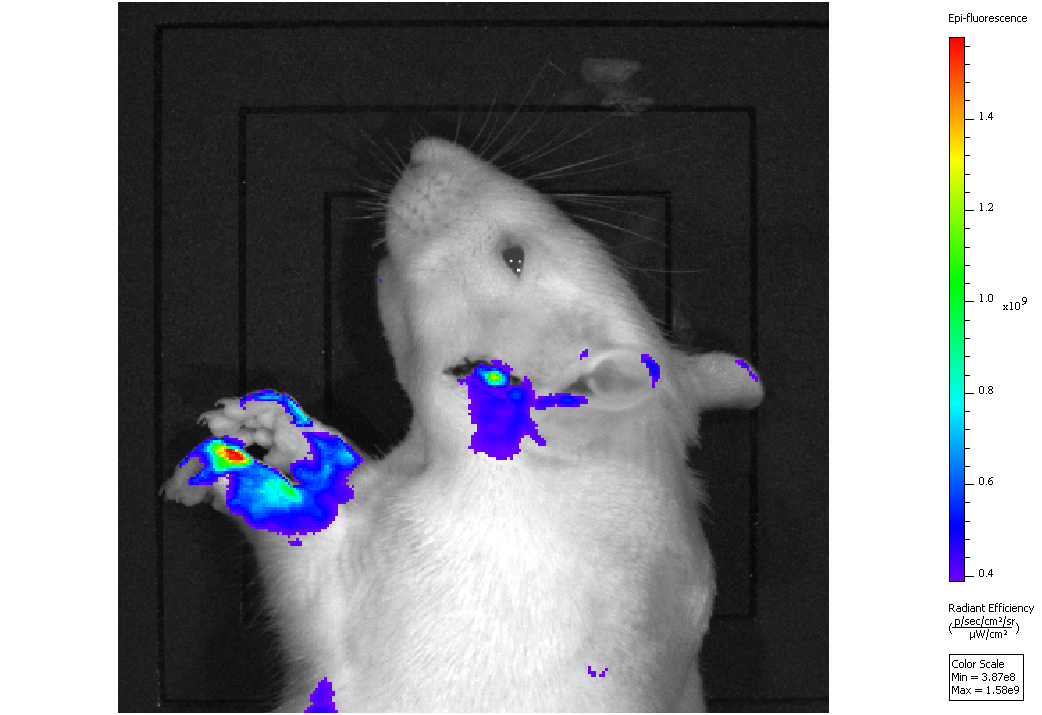

Supplement: Supplementary file 5 [file DataSheet1.ZIP › figure1/PbFGF-3d.tif]

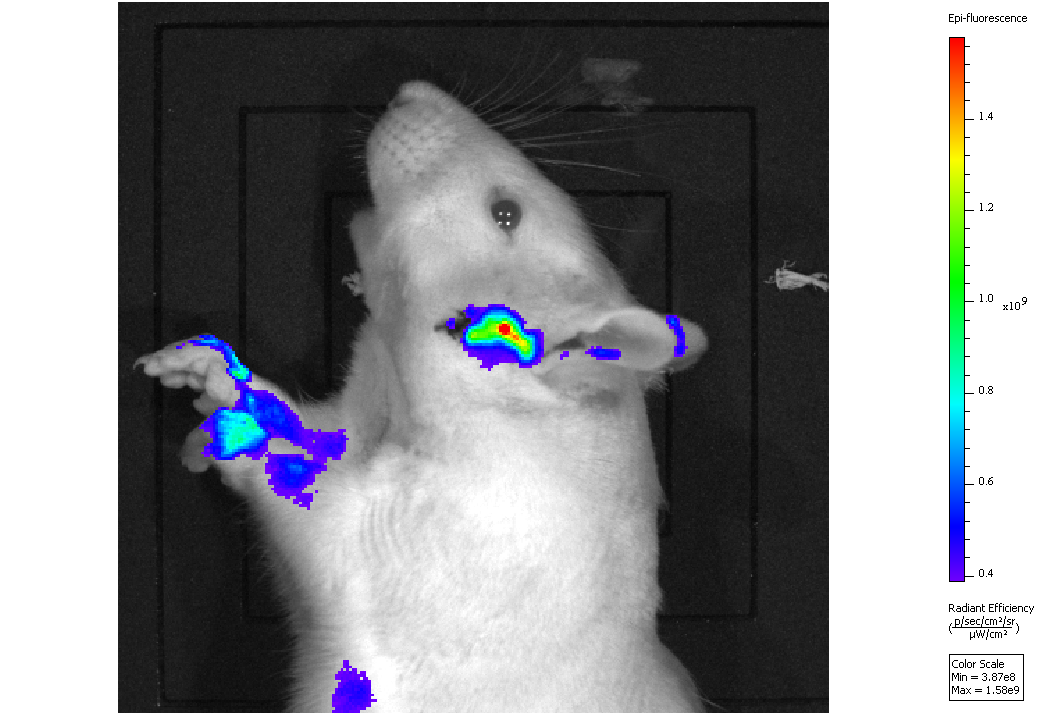

Supplement: Supplementary file 5 [file DataSheet1.ZIP › figure1/PbFGF-6h.tif]

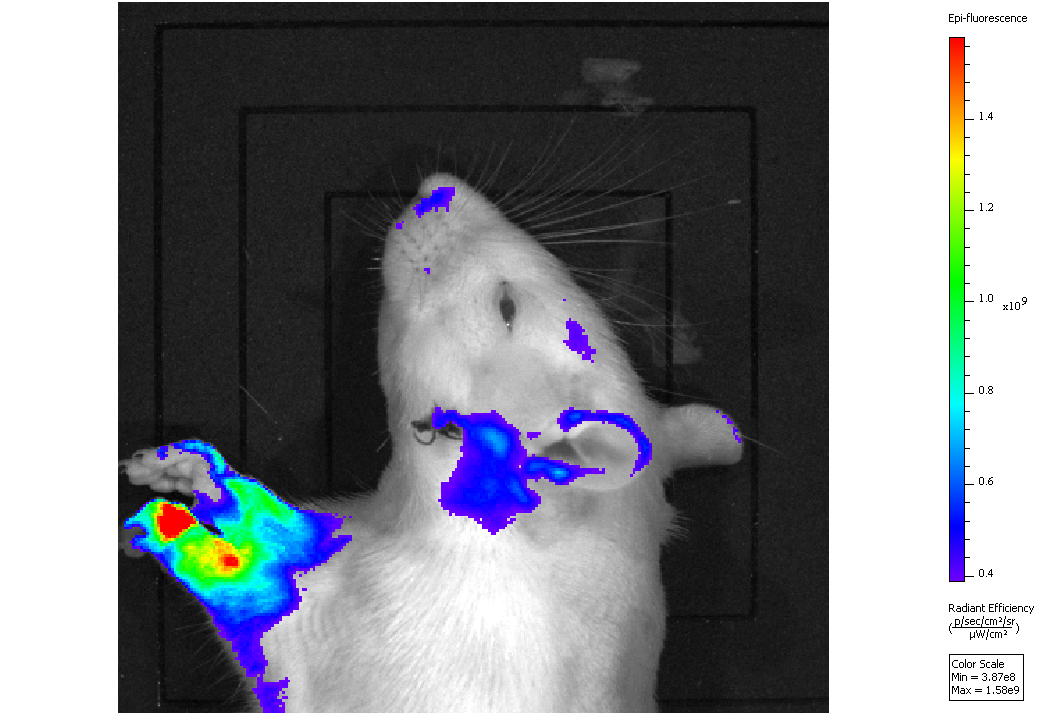

Supplement: Supplementary file 5 [file DataSheet1.ZIP › figure1/PbFGF-7d.tif]

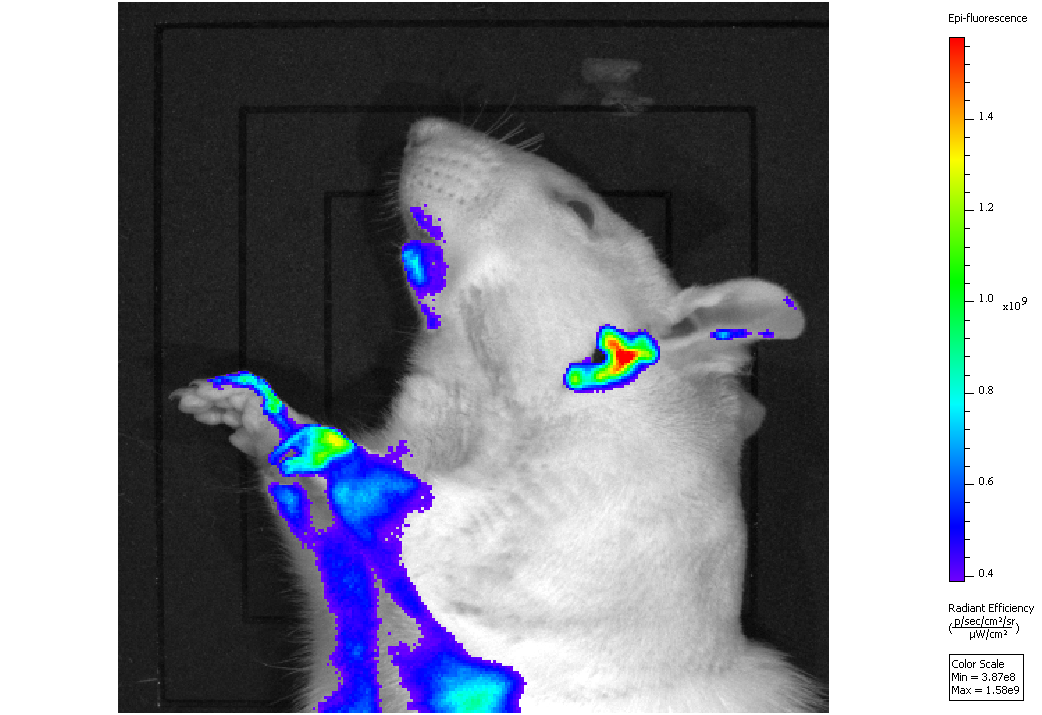

Supplement: Supplementary file 5 [file DataSheet1.ZIP › figure1/bFGF-0h.tif]

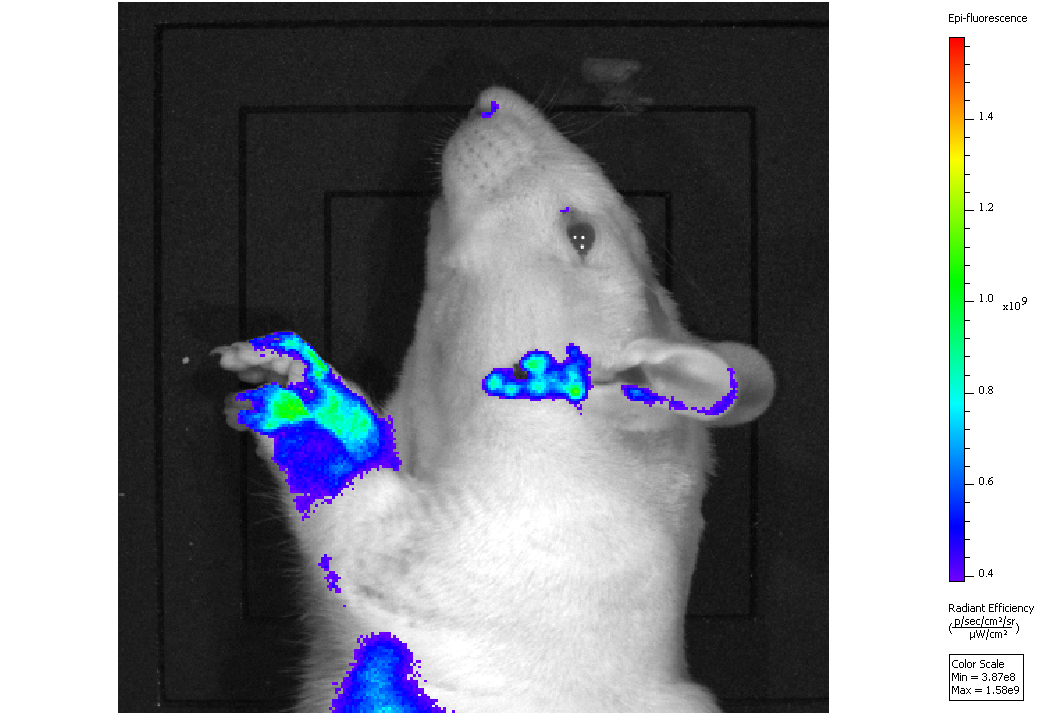

Supplement: Supplementary file 5 [file DataSheet1.ZIP › figure1/bFGF-1d.tif]

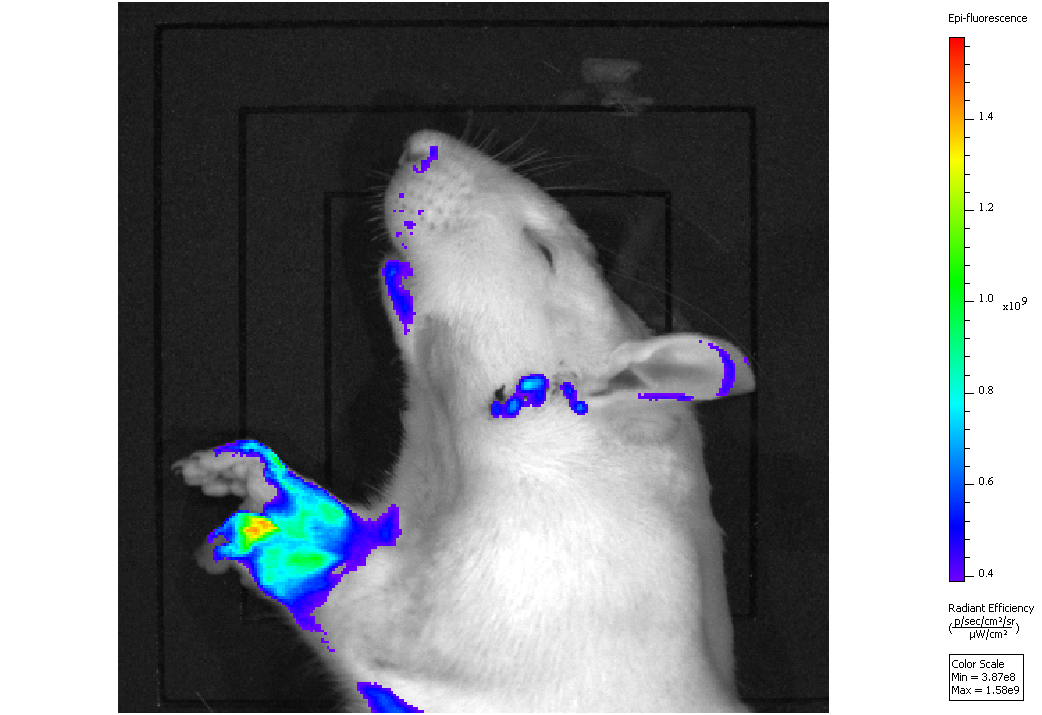

Supplement: Supplementary file 5 [file DataSheet1.ZIP › figure1/bfgf-3d.tif]

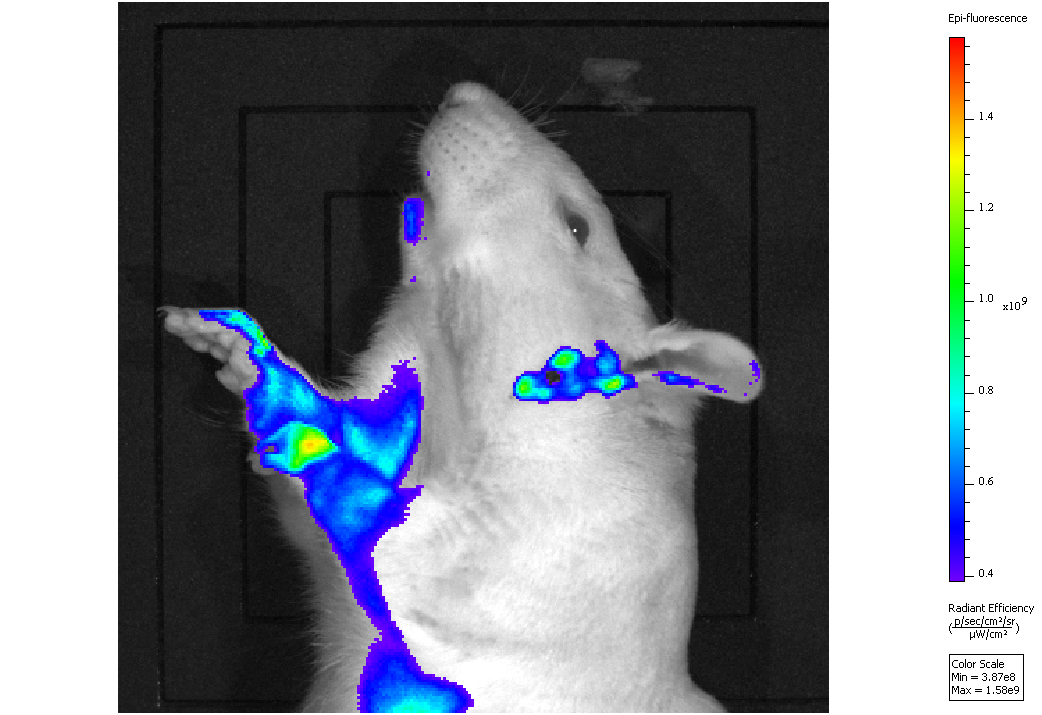

Supplement: Supplementary file 5 [file DataSheet1.ZIP › figure1/bfgf-6h.tif]

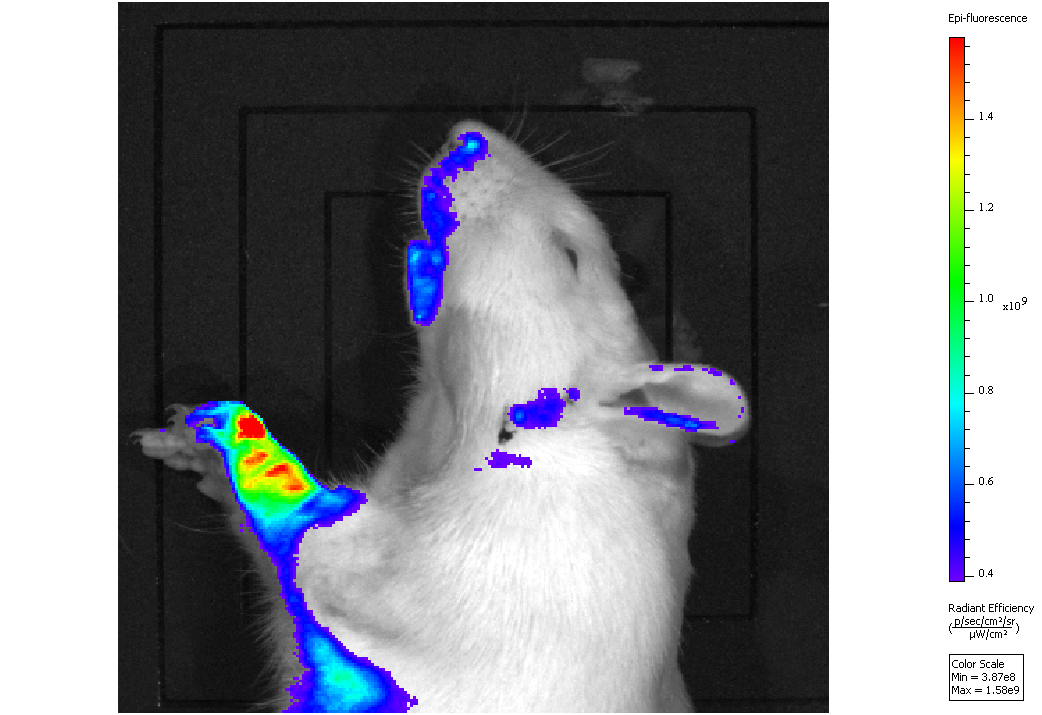

Supplement: Supplementary file 5 [file DataSheet1.ZIP › figure1/bfgf-7d.tif]

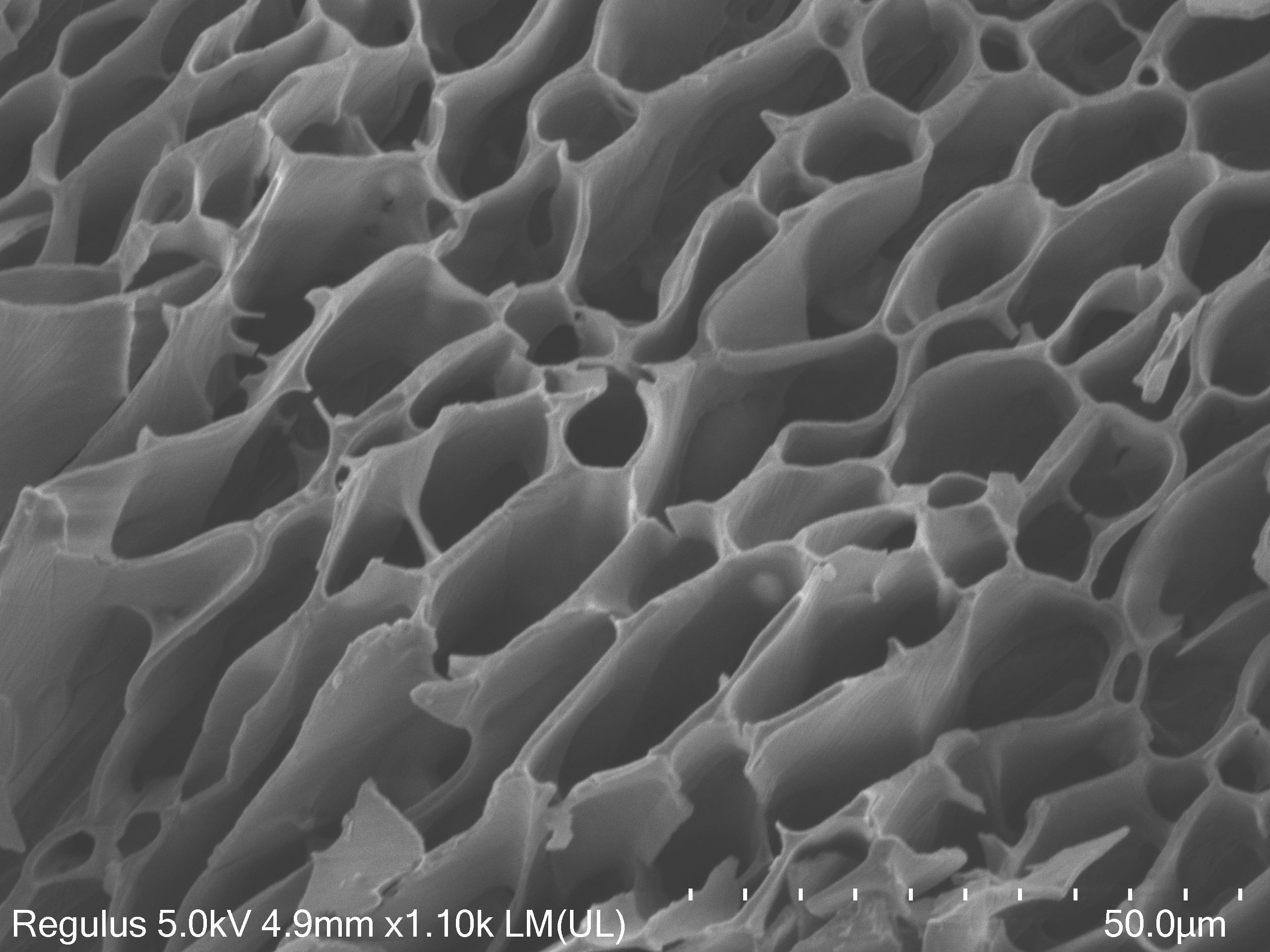

Supplement: Supplementary file 5 [file DataSheet1.ZIP › figure1/figure 1B (1).tif]

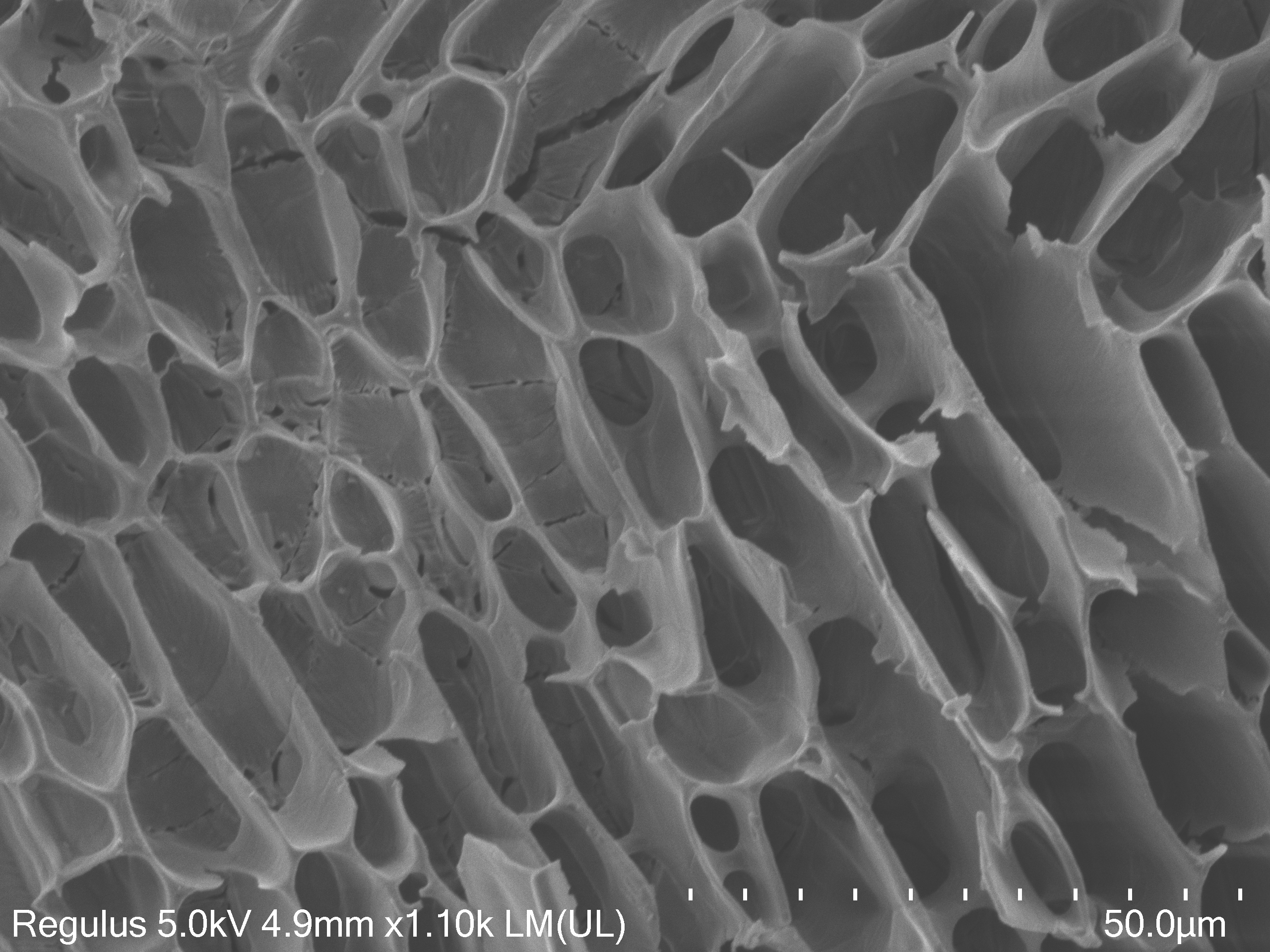

Supplement: Supplementary file 5 [file DataSheet1.ZIP › figure1/figure 1B (2).tif]

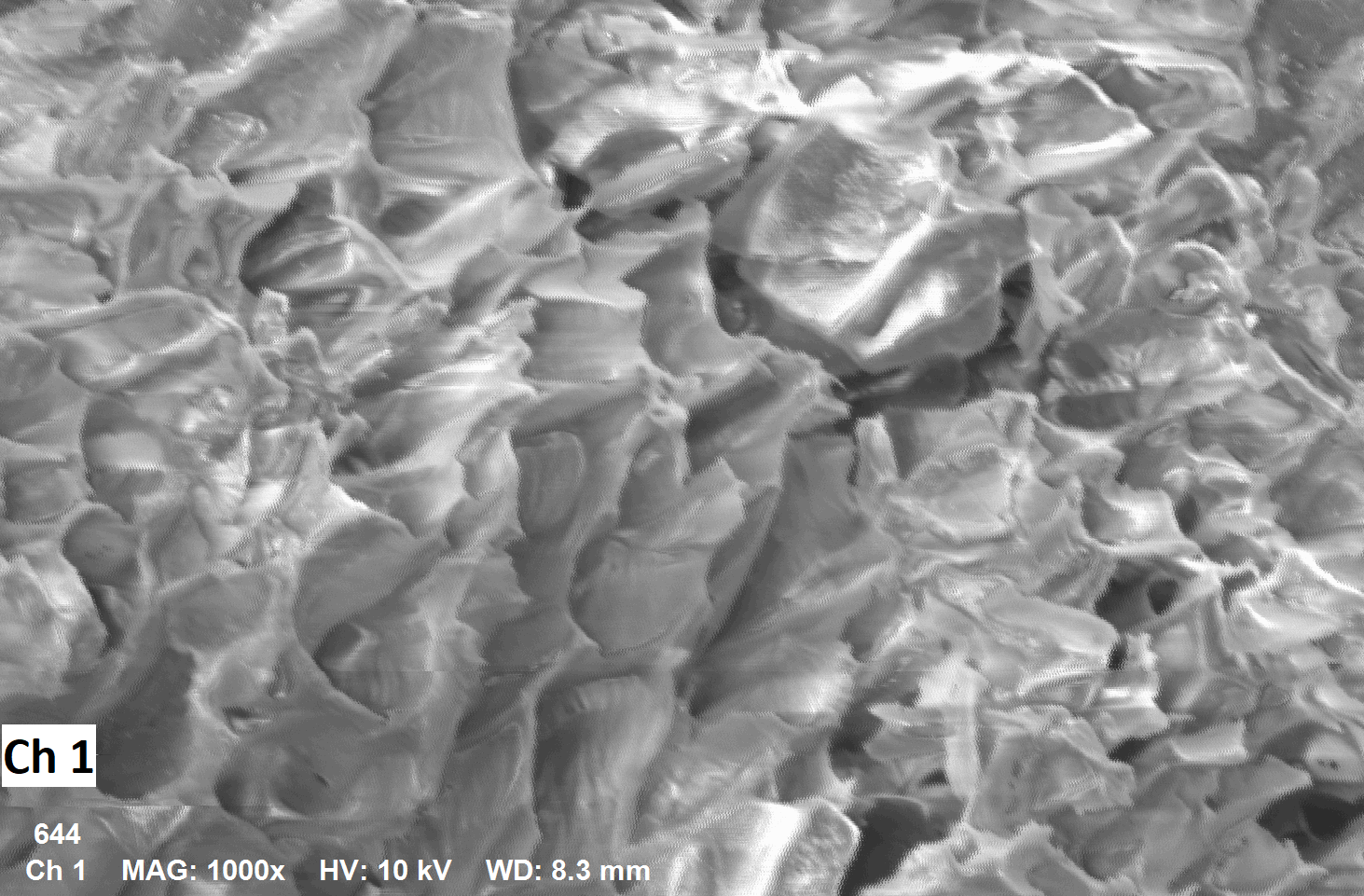

Supplement: Supplementary file 5 [file DataSheet1.ZIP › figure1/figure1C (1).bmp]

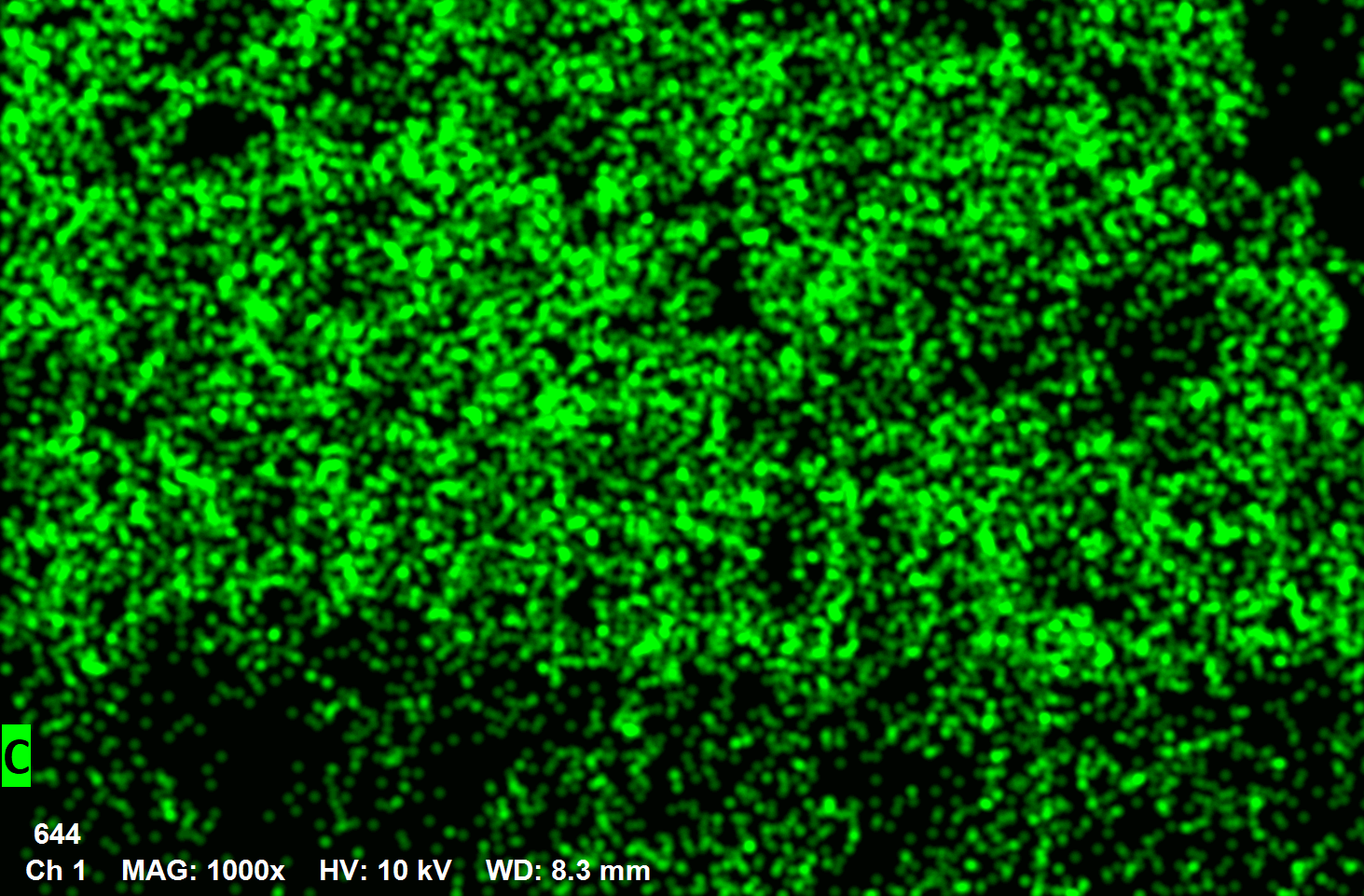

Supplement: Supplementary file 5 [file DataSheet1.ZIP › figure1/figure1C (2).bmp]

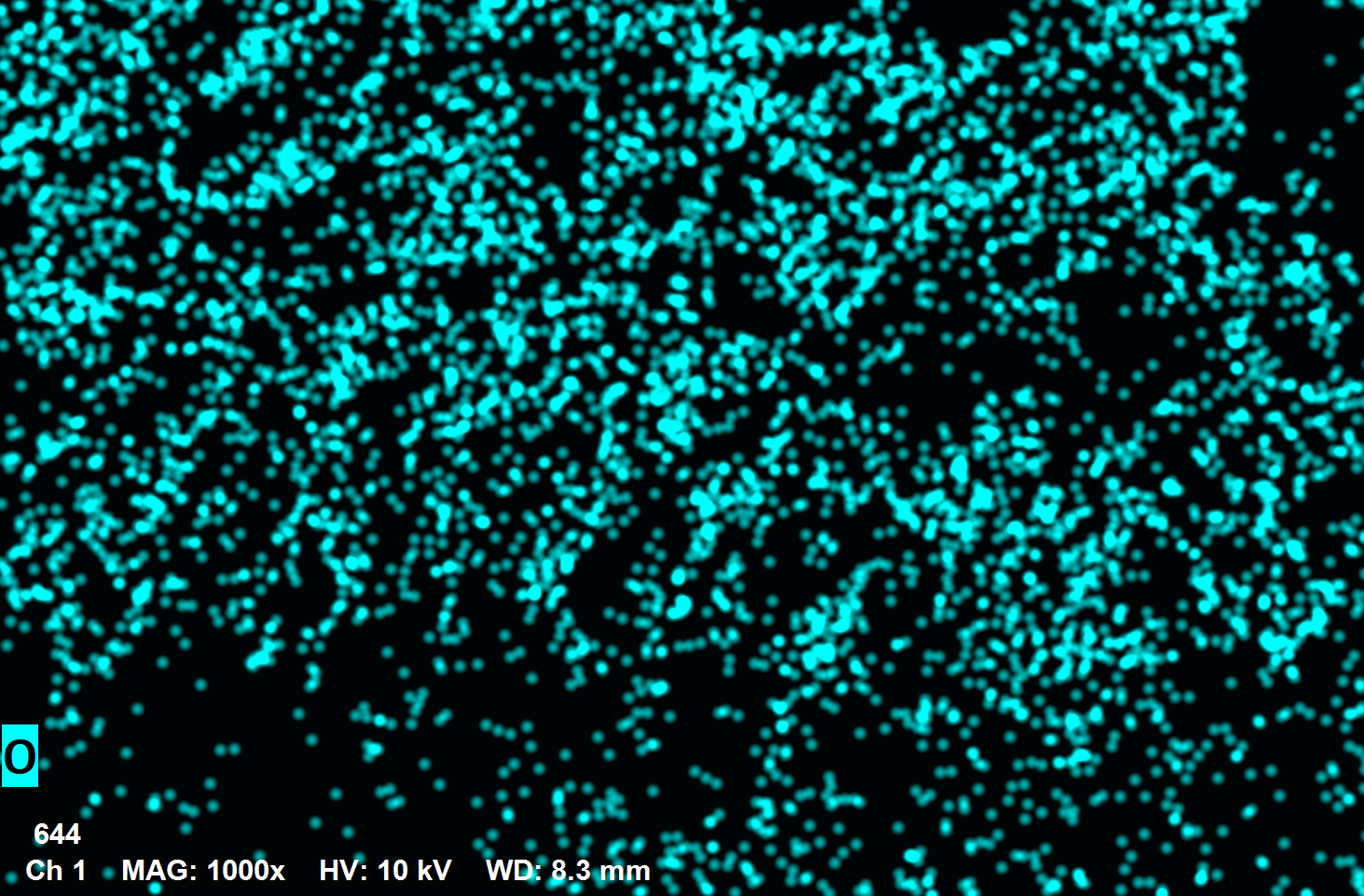

Supplement: Supplementary file 5 [file DataSheet1.ZIP › figure1/figure1C (3).bmp]

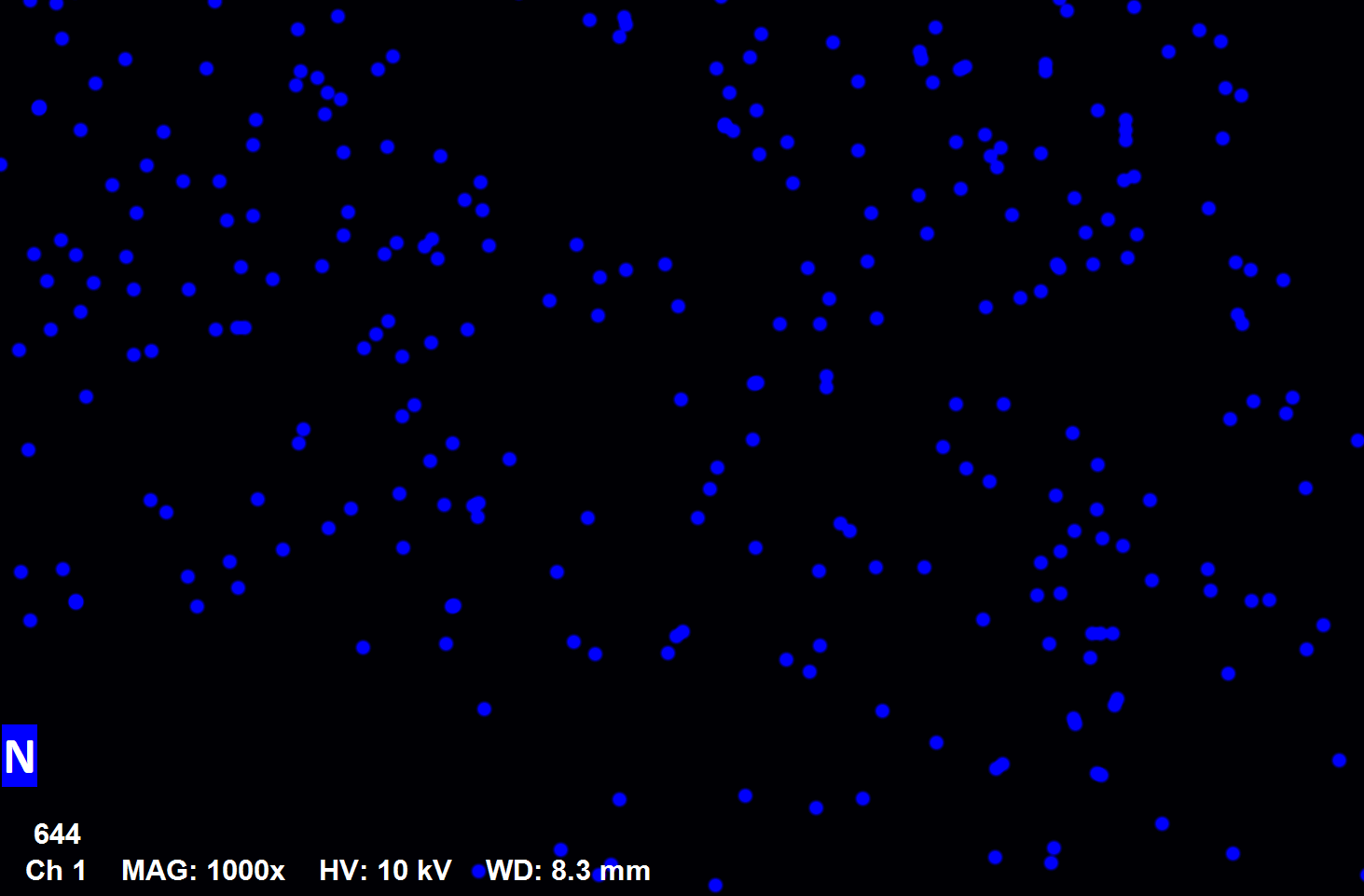

Supplement: Supplementary file 5 [file DataSheet1.ZIP › figure1/figure1C (4).bmp]

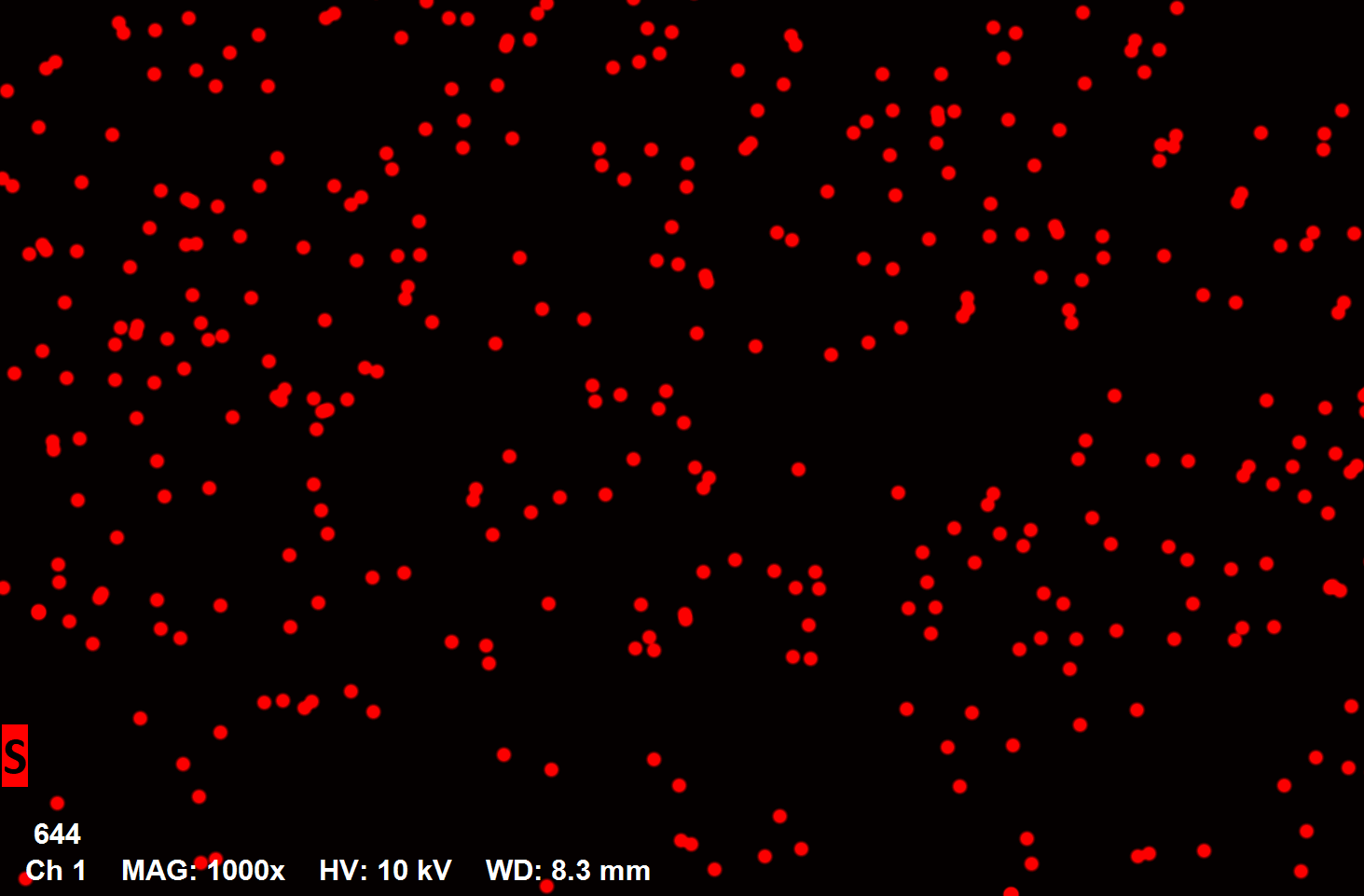

Supplement: Supplementary file 5 [file DataSheet1.ZIP › figure1/figure1C (5).bmp]

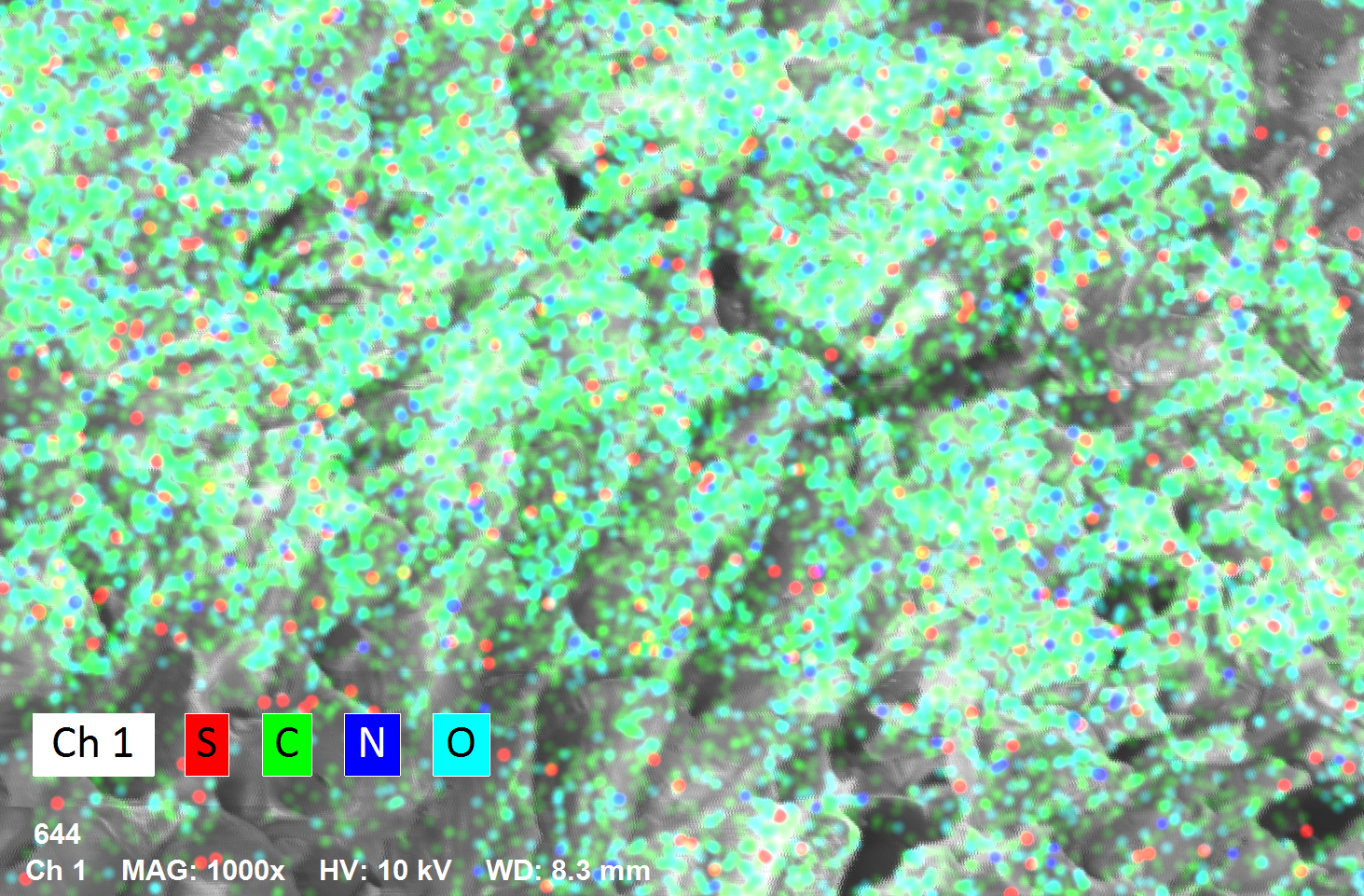

Supplement: Supplementary file 5 [file DataSheet1.ZIP › figure1/figure1C (6).bmp]

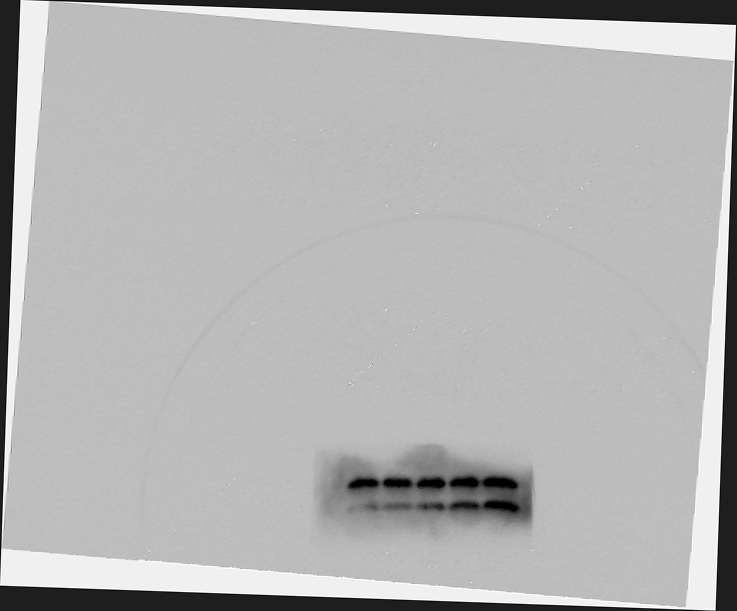

Supplement: Supplementary file 6 [file DataSheet6.ZIP › Figure3/LC3.tif]

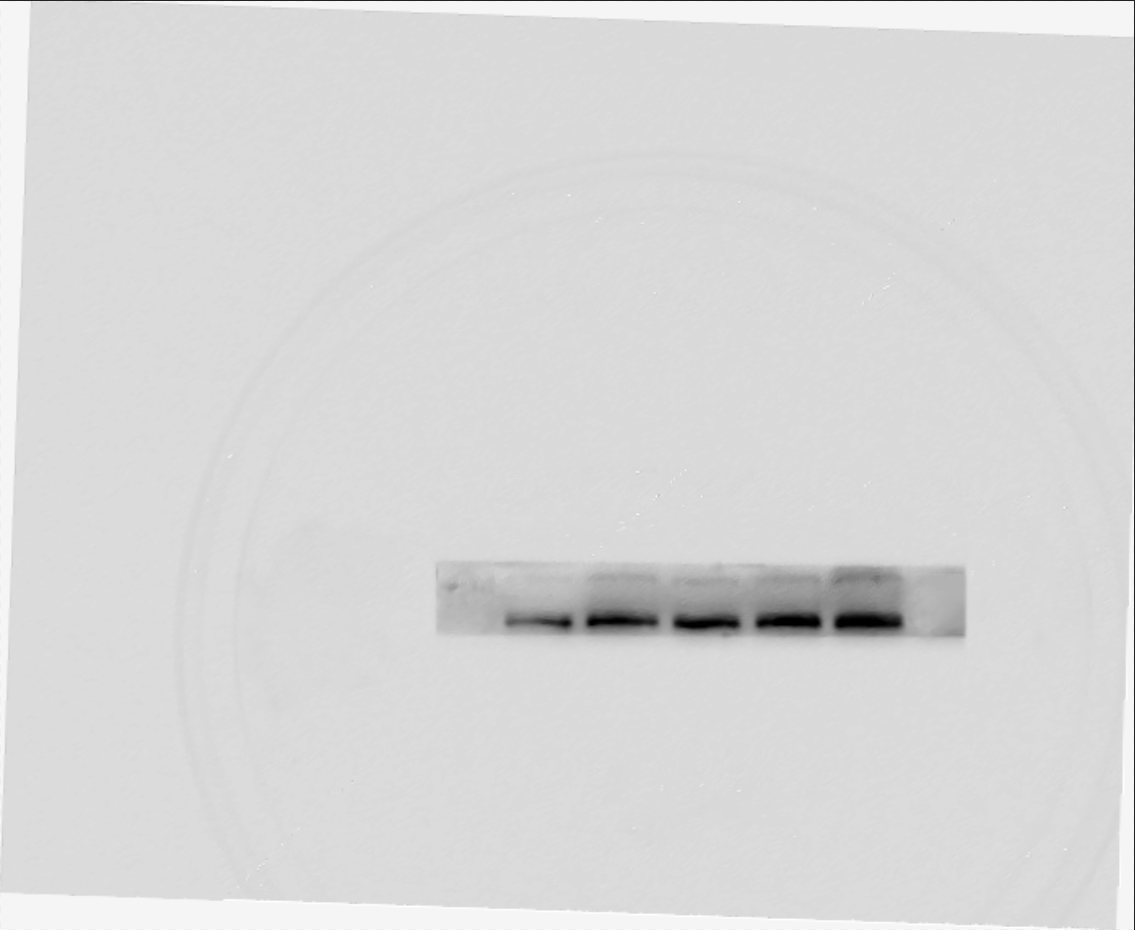

Supplement: Supplementary file 6 [file DataSheet6.ZIP › Figure3/figure 3 ATG5.png]

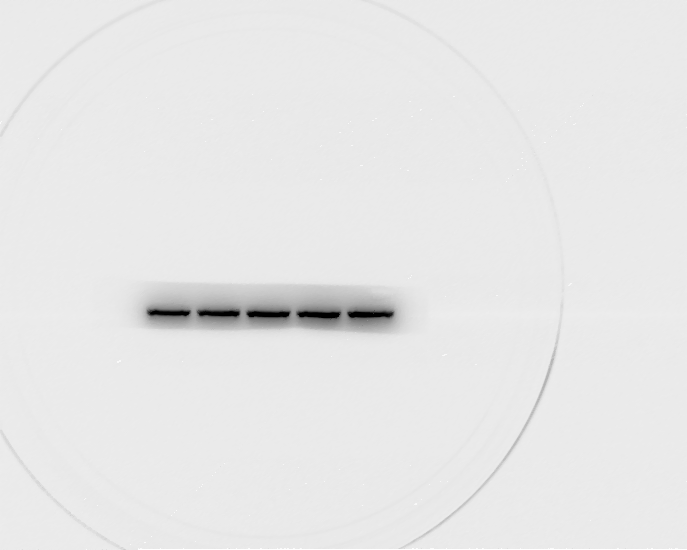

Supplement: Supplementary file 6 [file DataSheet6.ZIP › Figure3/figure 3 GAPDH.tif]

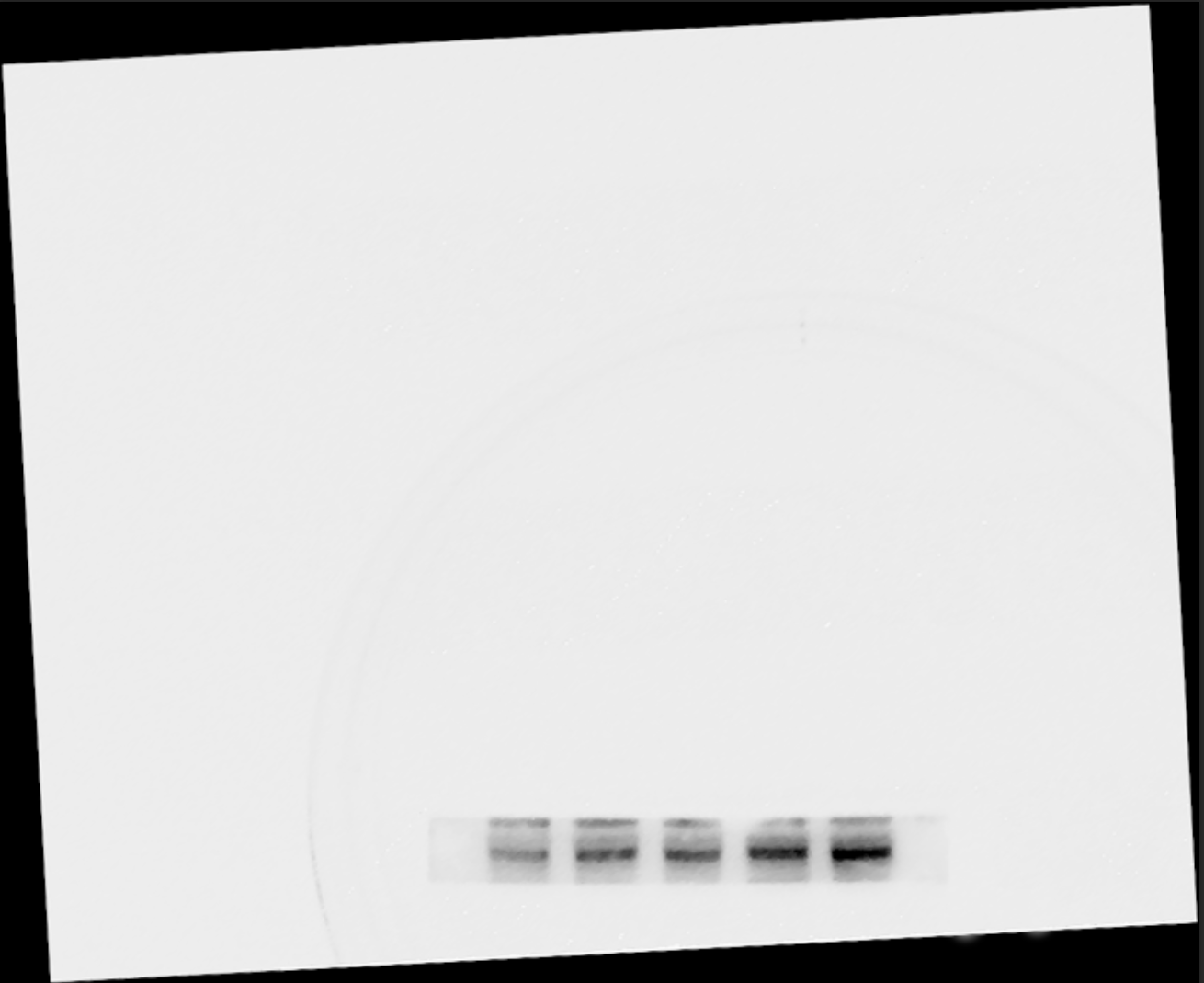

Supplement: Supplementary file 6 [file DataSheet6.ZIP › Figure3/figure 3 beclin1 .png]

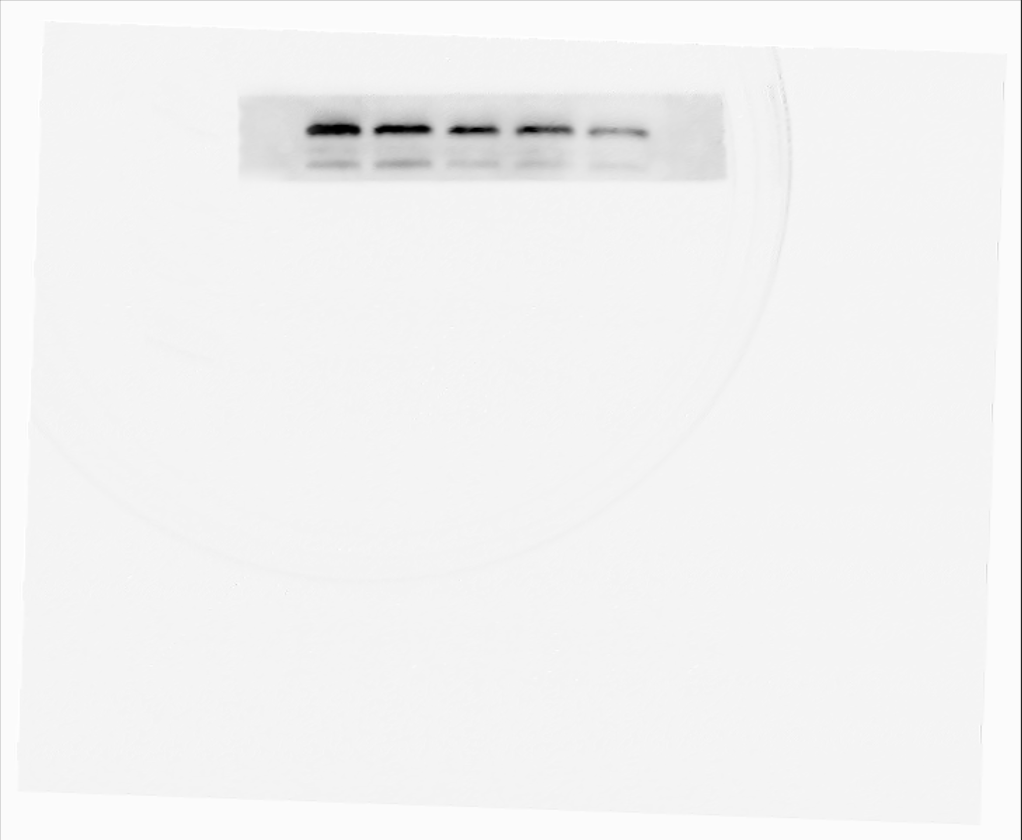

Supplement: Supplementary file 6 [file DataSheet6.ZIP › Figure3/figure 3 p62.png]

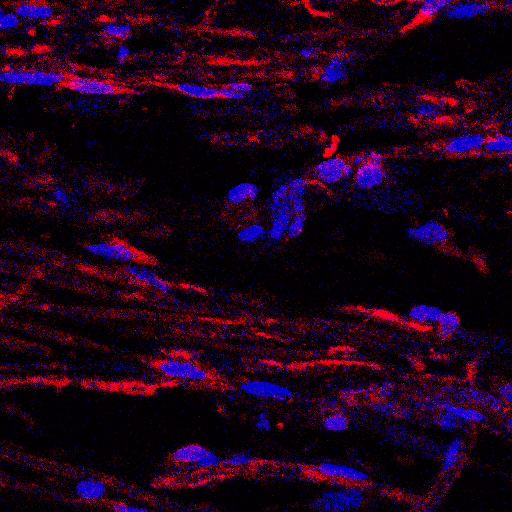

Supplement: Supplementary file 6 [file DataSheet6.ZIP › Figure3/figure 3A FNI (1).tiff]

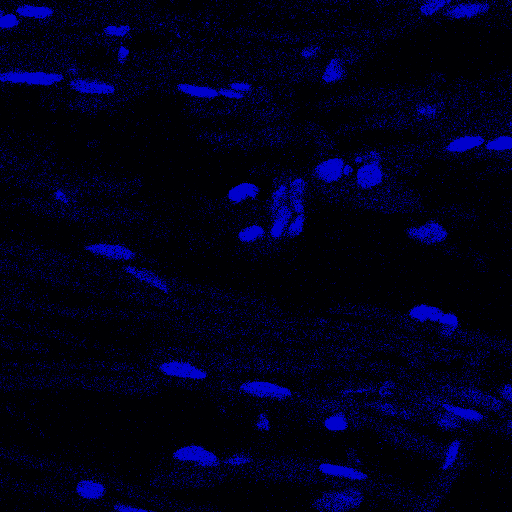

Supplement: Supplementary file 6 [file DataSheet6.ZIP › Figure3/figure 3A FNI (2).tiff]

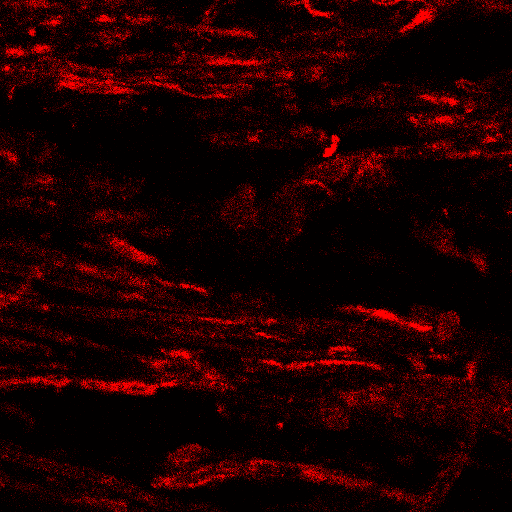

Supplement: Supplementary file 6 [file DataSheet6.ZIP › Figure3/figure 3A FNI (3).tiff]

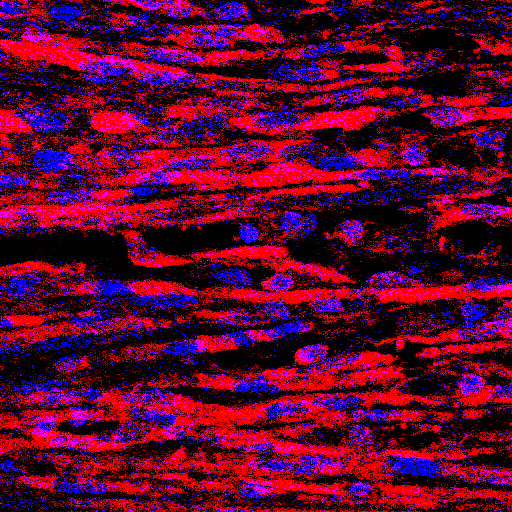

Supplement: Supplementary file 6 [file DataSheet6.ZIP › Figure3/figure 3A P-bFGF (1).tiff]

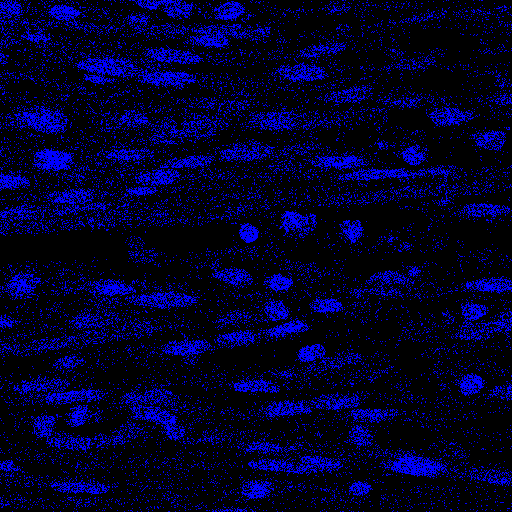

Supplement: Supplementary file 6 [file DataSheet6.ZIP › Figure3/figure 3A P-bFGF (2).tiff]

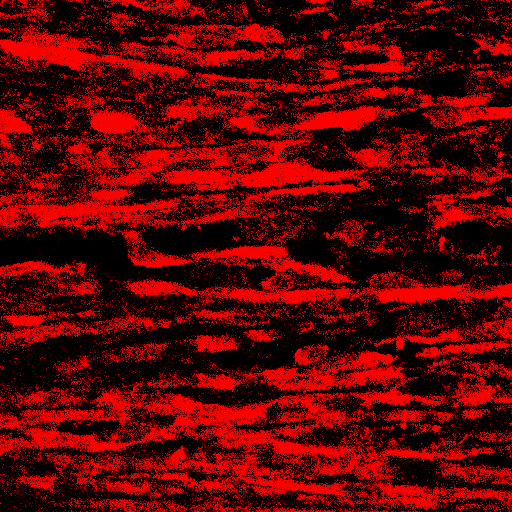

Supplement: Supplementary file 6 [file DataSheet6.ZIP › Figure3/figure 3A P-bFGF (3).tiff]

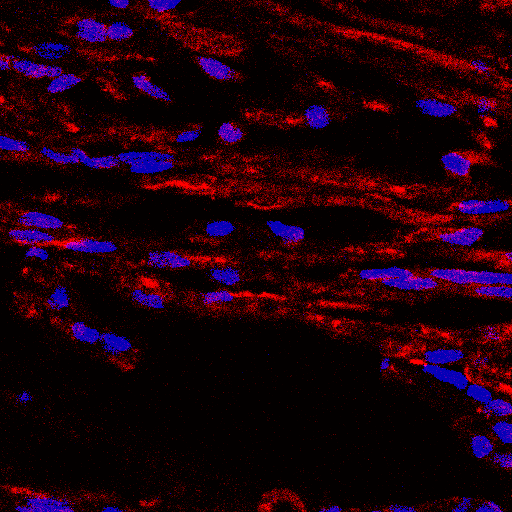

Supplement: Supplementary file 6 [file DataSheet6.ZIP › Figure3/figure 3A Poloxamer (1).tiff]

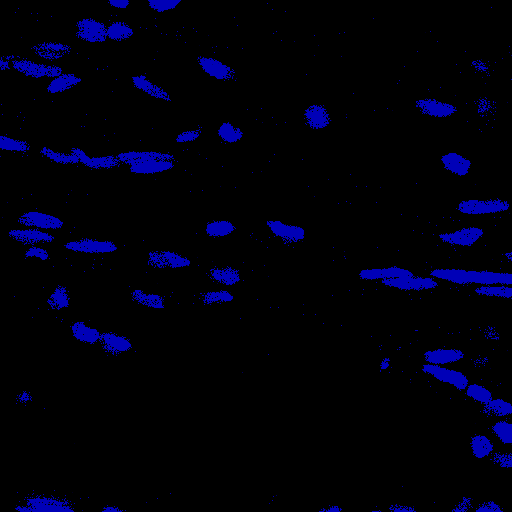

Supplement: Supplementary file 6 [file DataSheet6.ZIP › Figure3/figure 3A Poloxamer (2).tiff]

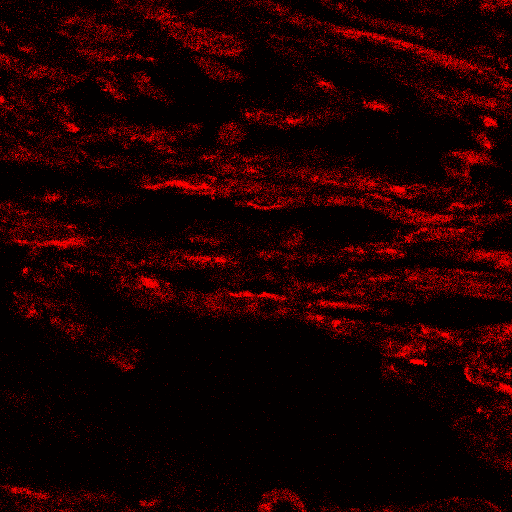

Supplement: Supplementary file 6 [file DataSheet6.ZIP › Figure3/figure 3A Poloxamer (3).tiff]

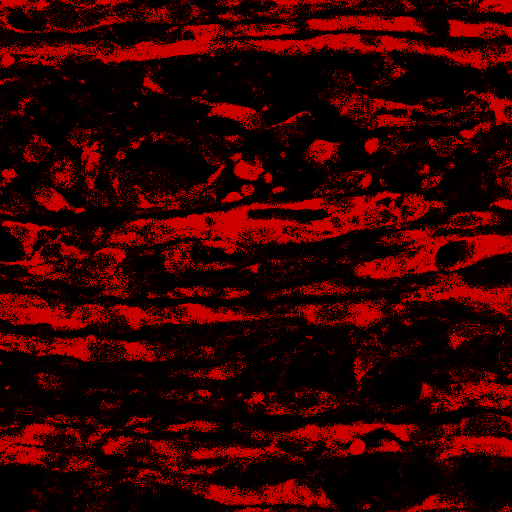

Supplement: Supplementary file 6 [file DataSheet6.ZIP › Figure3/figure 3A bFGF (1).tiff]

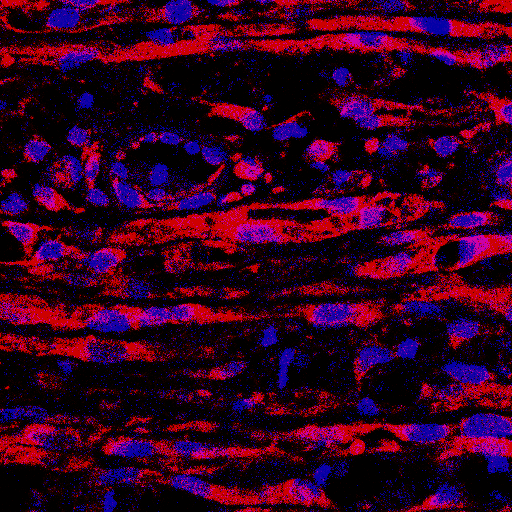

Supplement: Supplementary file 6 [file DataSheet6.ZIP › Figure3/figure 3A bFGF (2).tiff]

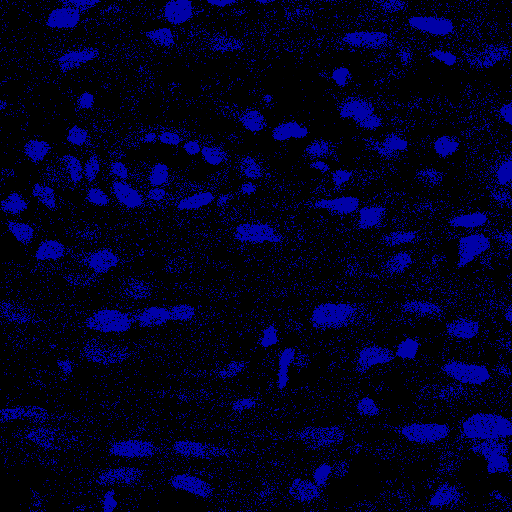

Supplement: Supplementary file 6 [file DataSheet6.ZIP › Figure3/figure 3A bFGF (3).tiff]

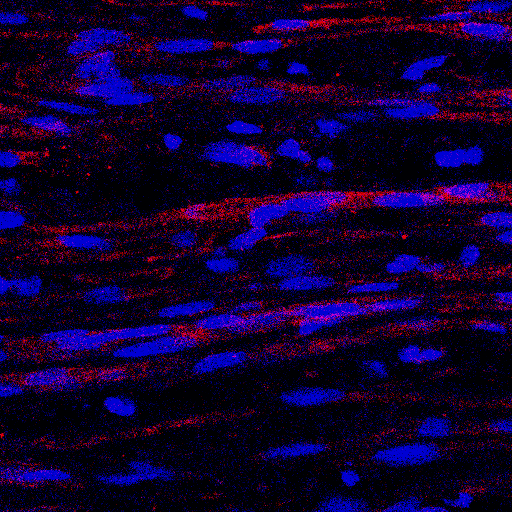

Supplement: Supplementary file 6 [file DataSheet6.ZIP › Figure3/figure 3A sham (1).tiff]

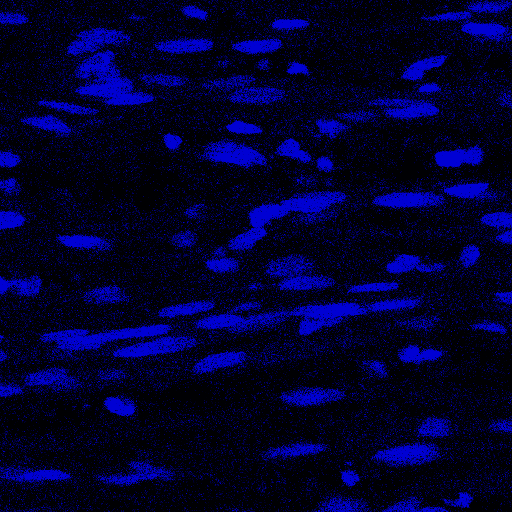

Supplement: Supplementary file 6 [file DataSheet6.ZIP › Figure3/figure 3A sham (2).tiff]

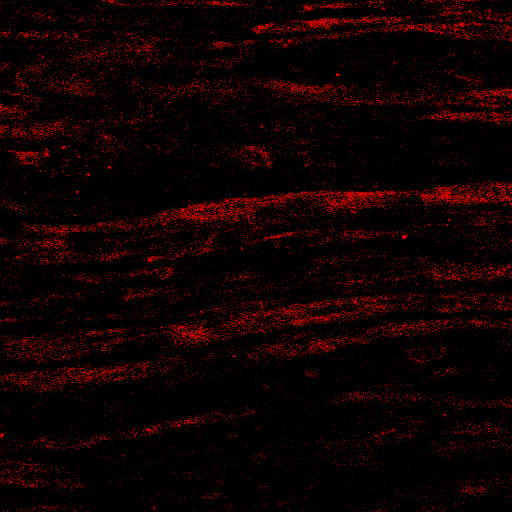

Supplement: Supplementary file 6 [file DataSheet6.ZIP › Figure3/figure 3A sham (3).tiff]

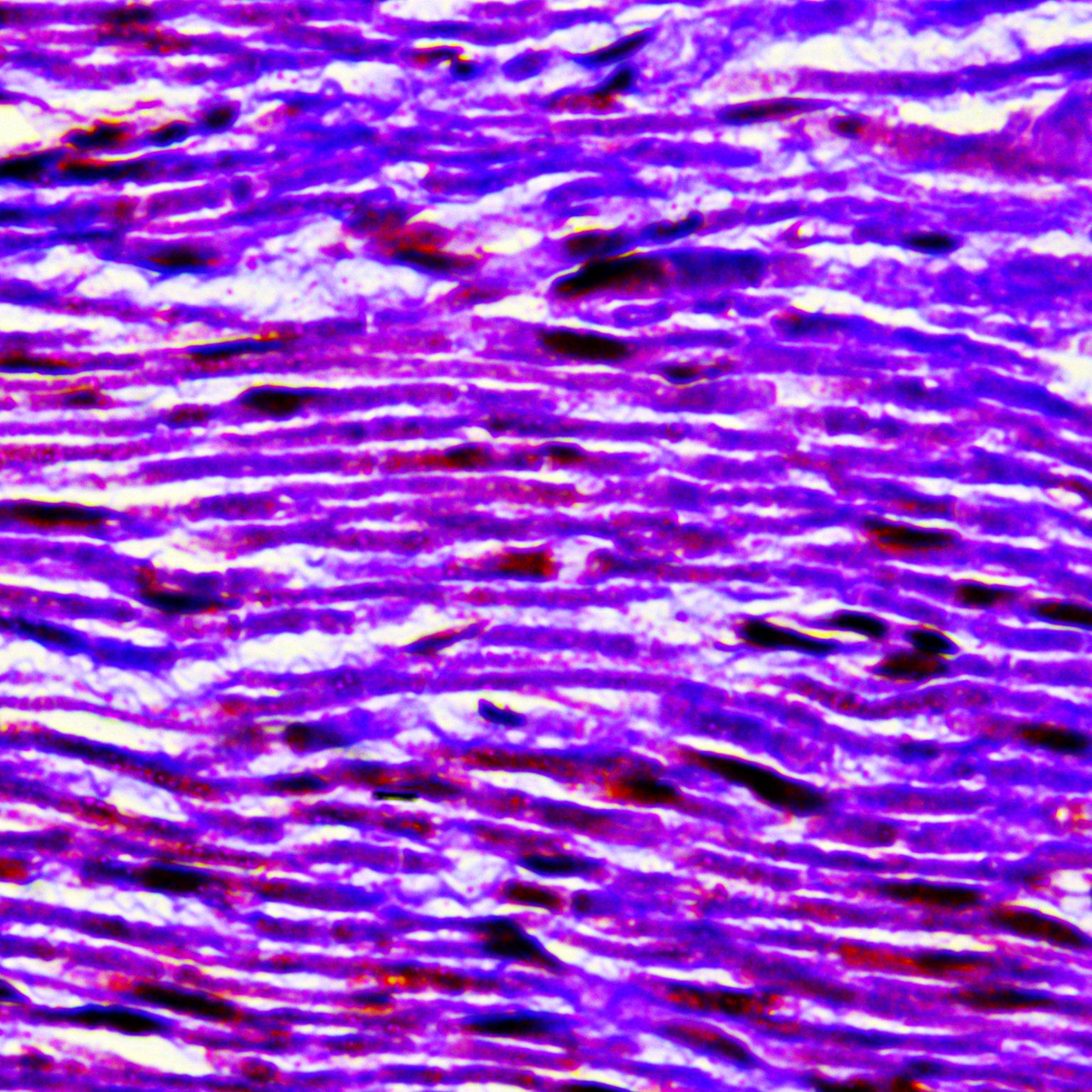

Supplement: Supplementary file 7 [file DataSheet2.ZIP › figure 2B BMASSON (1).jpg]

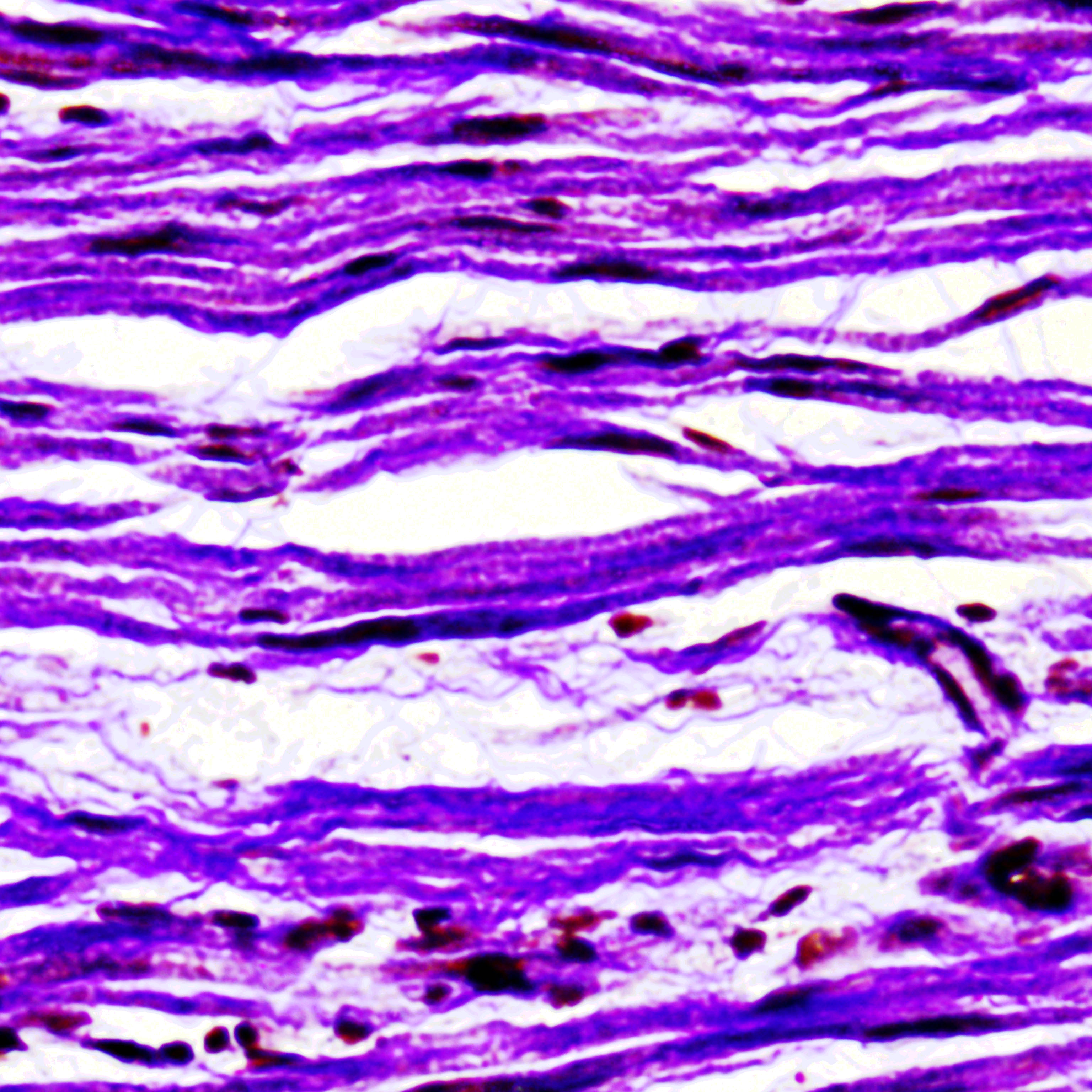

Supplement: Supplementary file 7 [file DataSheet2.ZIP › figure 2B BMASSON (2).jpg]

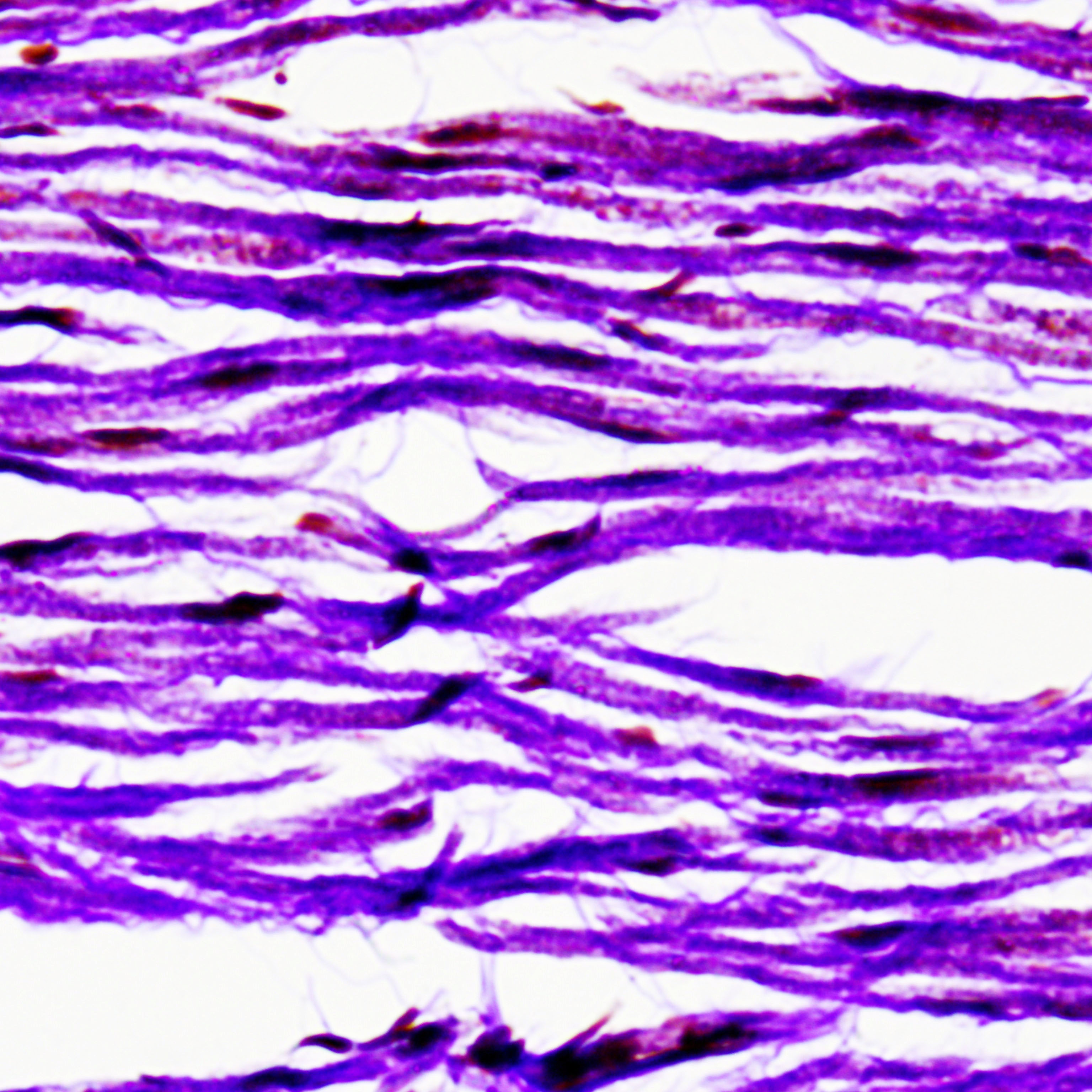

Supplement: Supplementary file 7 [file DataSheet2.ZIP › figure 2B BMASSON (3).jpg]

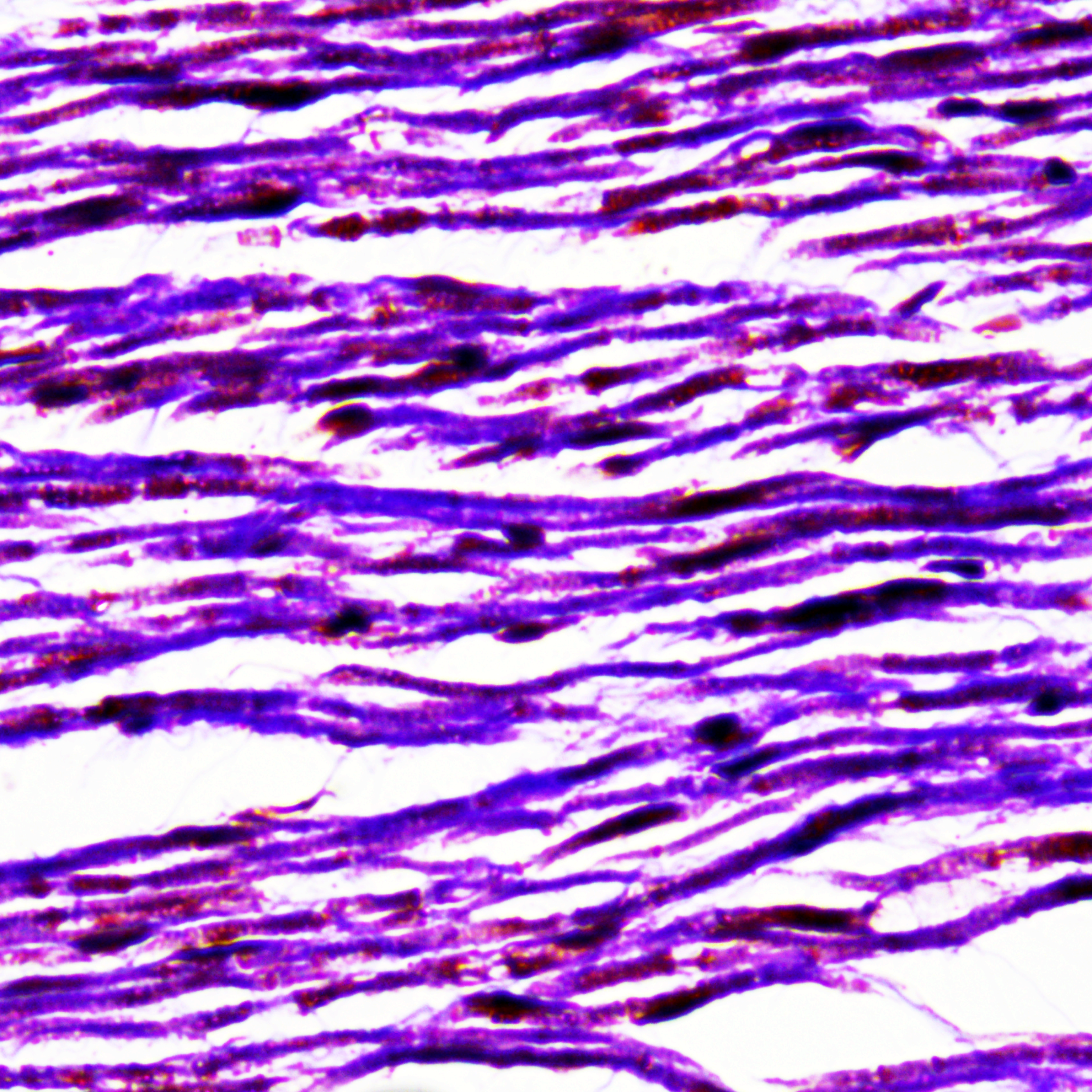

Supplement: Supplementary file 7 [file DataSheet2.ZIP › figure 2B BMASSON (4).jpg]

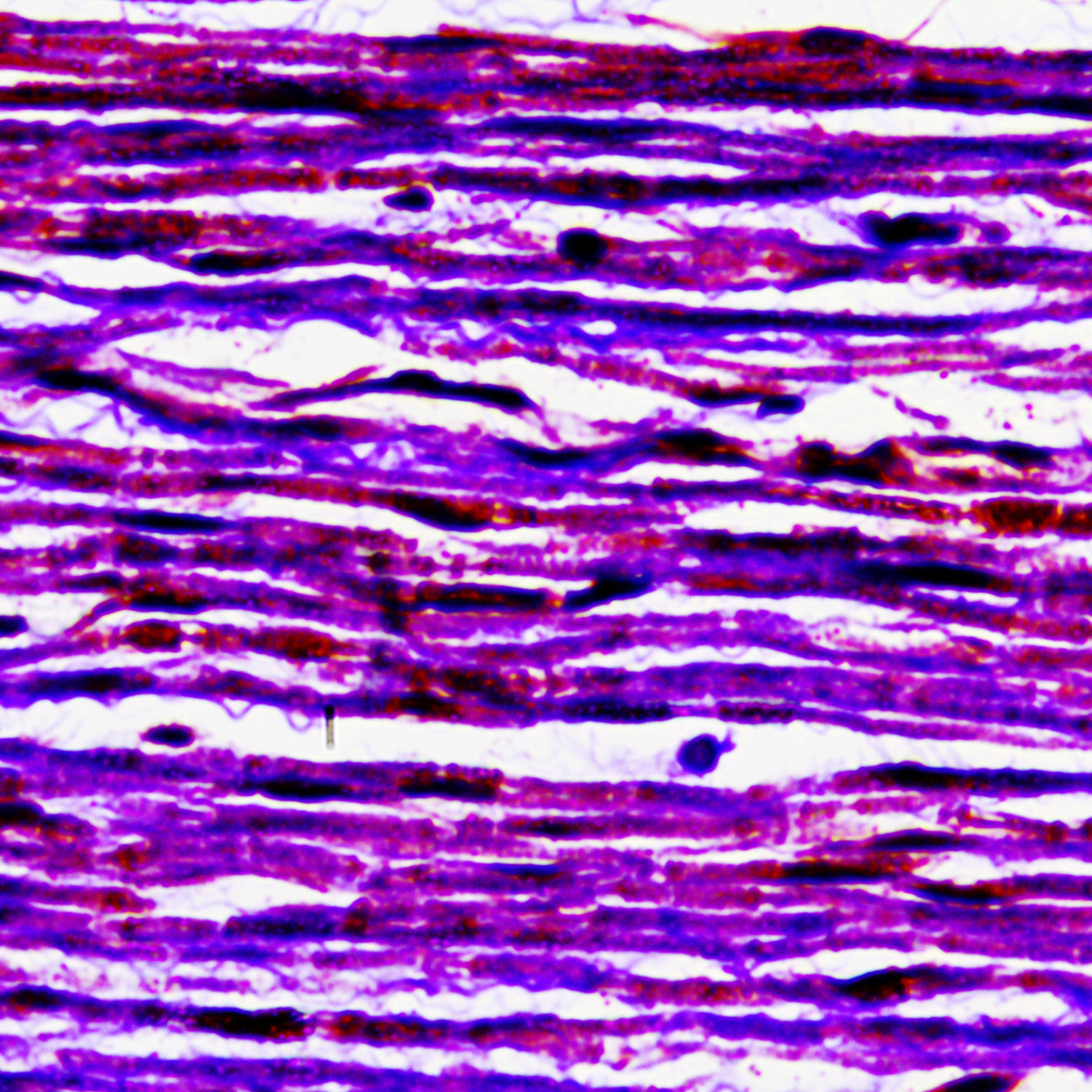

Supplement: Supplementary file 7 [file DataSheet2.ZIP › figure 2B BMASSON (5).jpg]

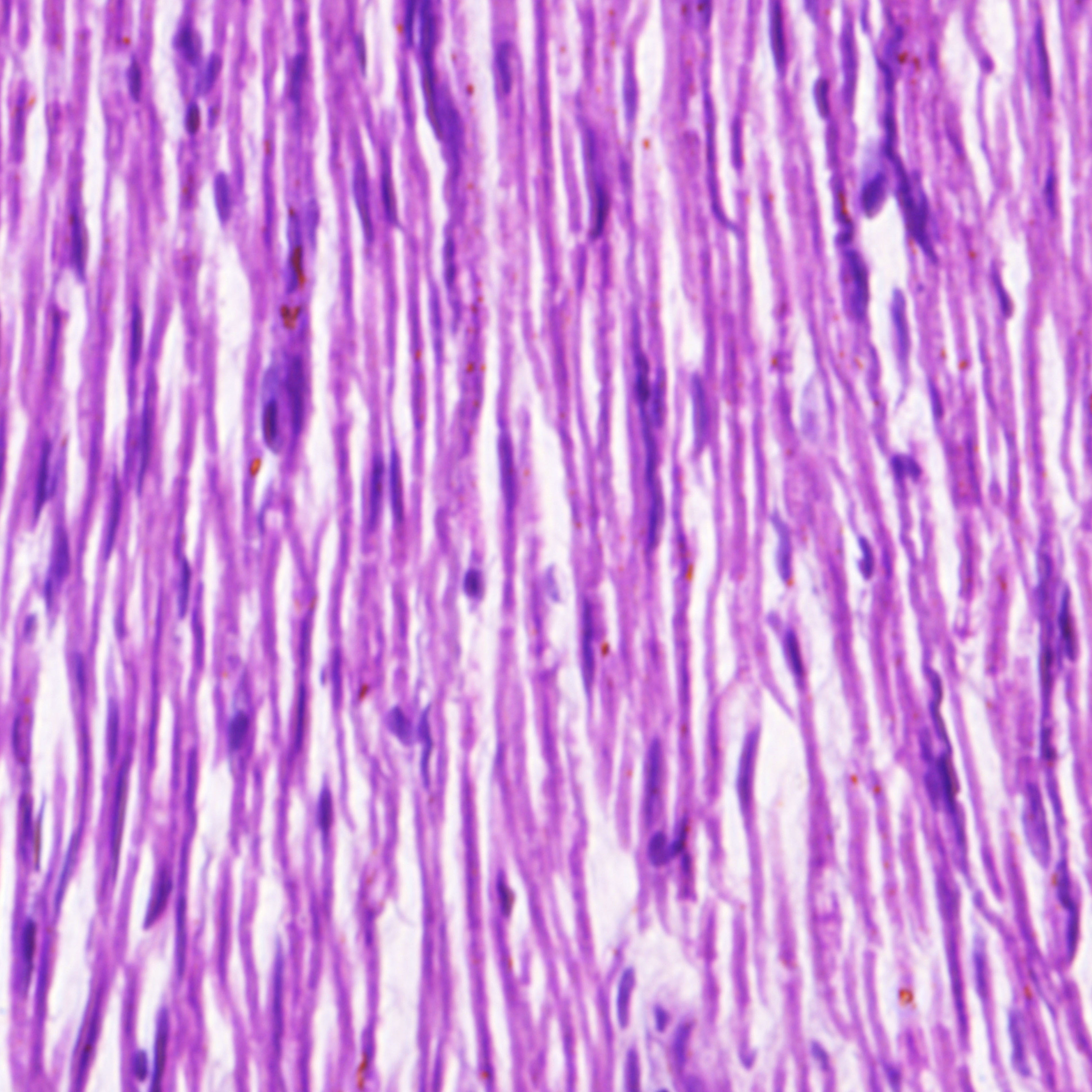

Supplement: Supplementary file 7 [file DataSheet2.ZIP › figure 2B HE (1).jpg]

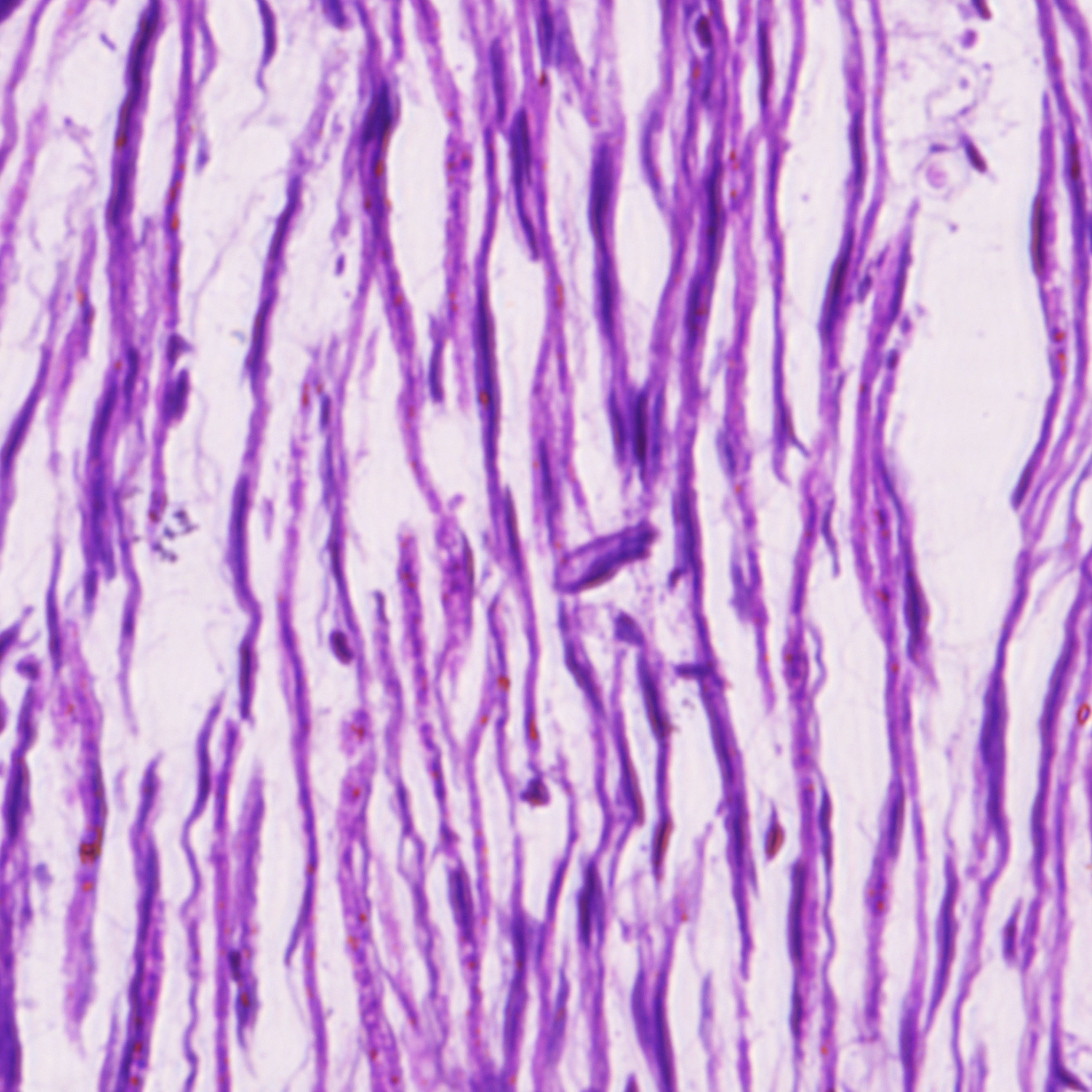

Supplement: Supplementary file 7 [file DataSheet2.ZIP › figure 2B HE (2).jpg]

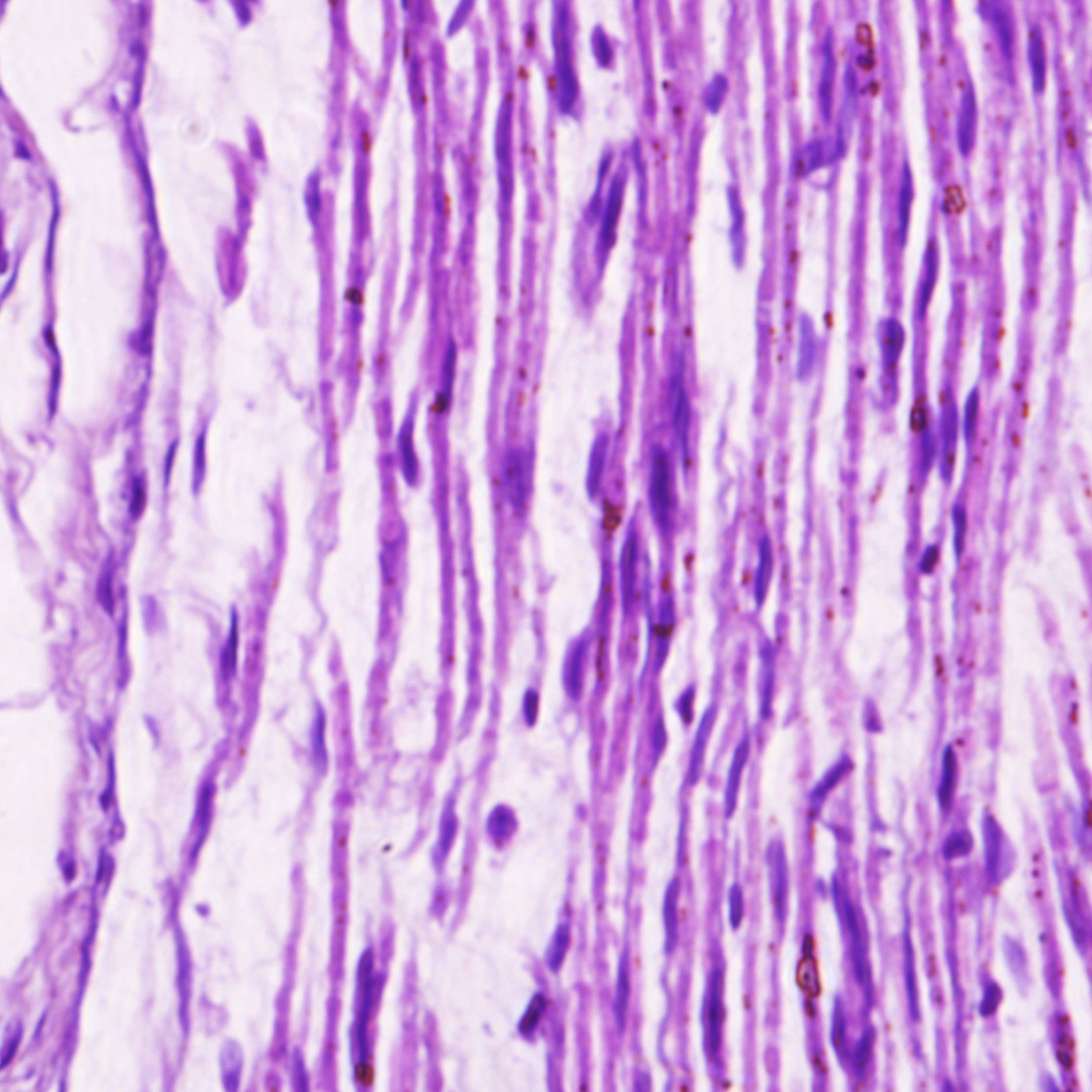

Supplement: Supplementary file 7 [file DataSheet2.ZIP › figure 2B HE (3).jpg]

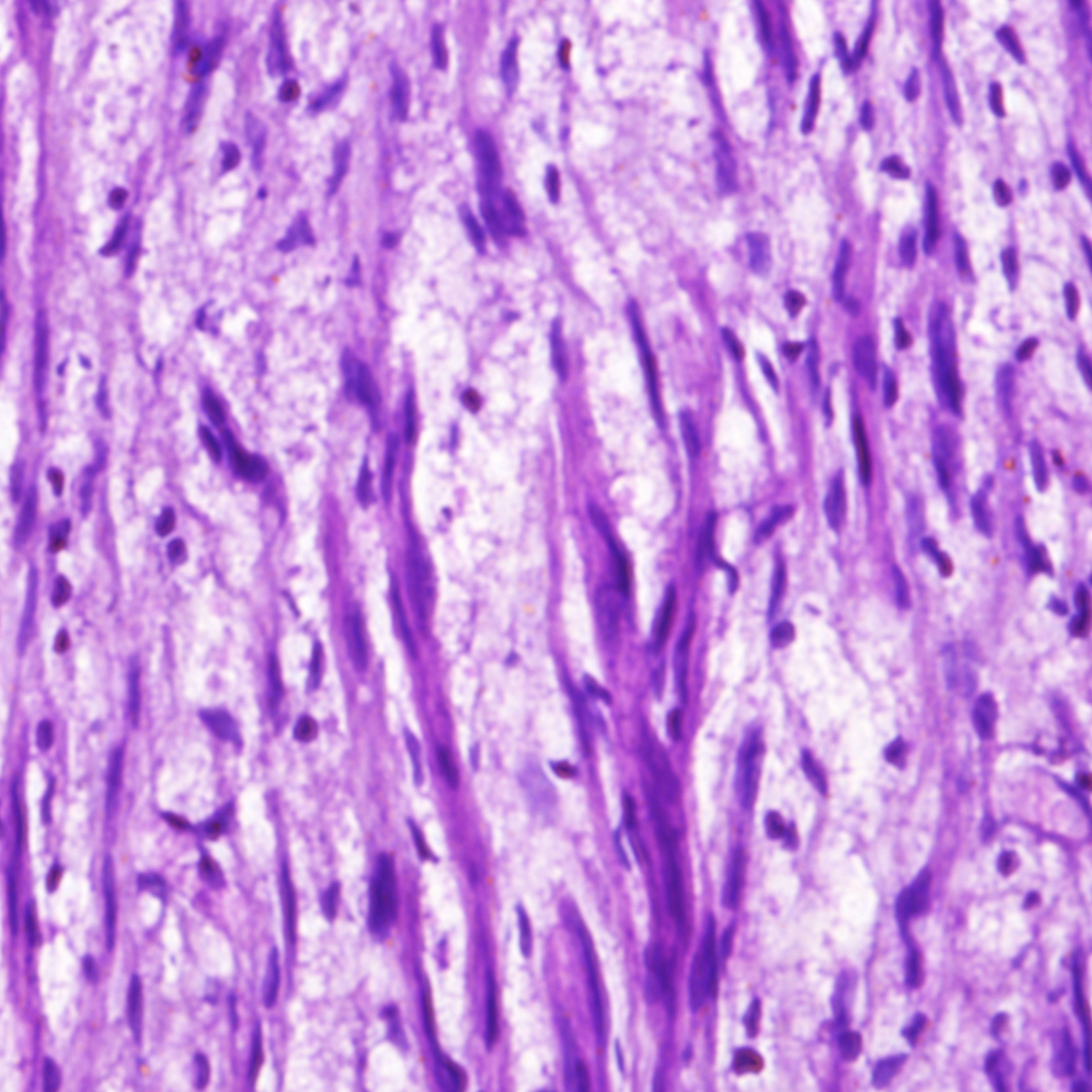

Supplement: Supplementary file 7 [file DataSheet2.ZIP › figure 2B HE (4).jpg]

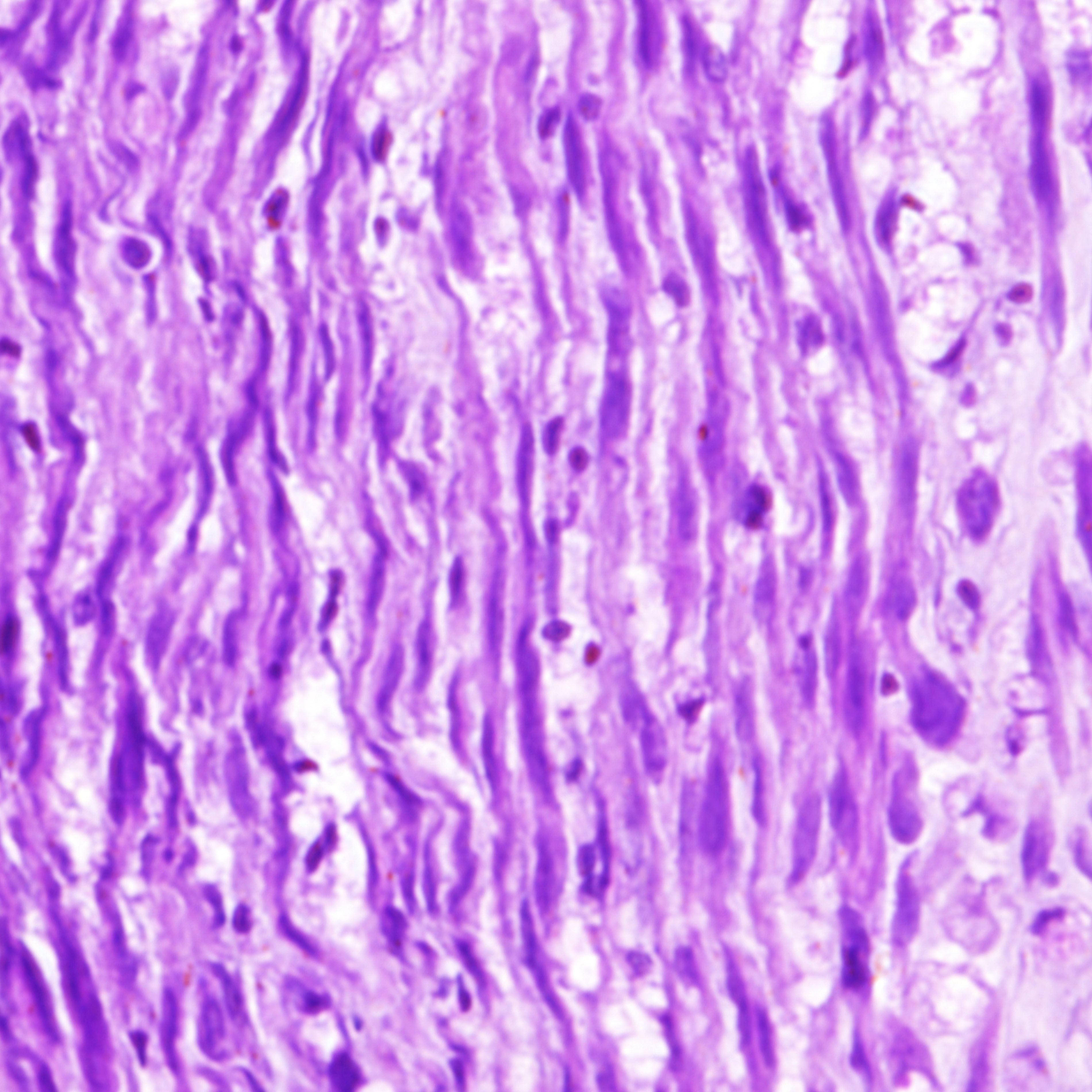

Supplement: Supplementary file 7 [file DataSheet2.ZIP › figure 2B HE (5).jpg]

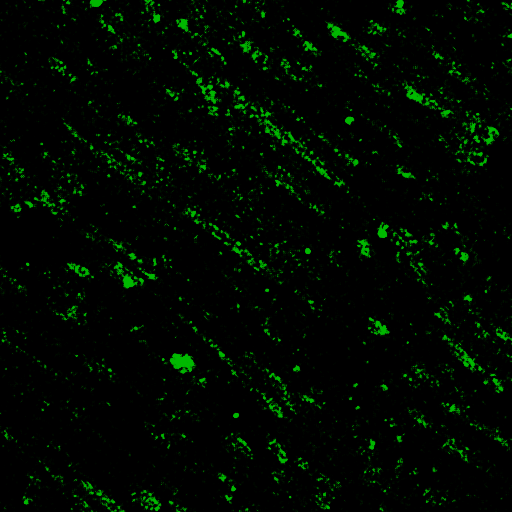

Supplement: Supplementary file 8 [file DataSheet5.ZIP › figure 2C bfgf (2).tiff]

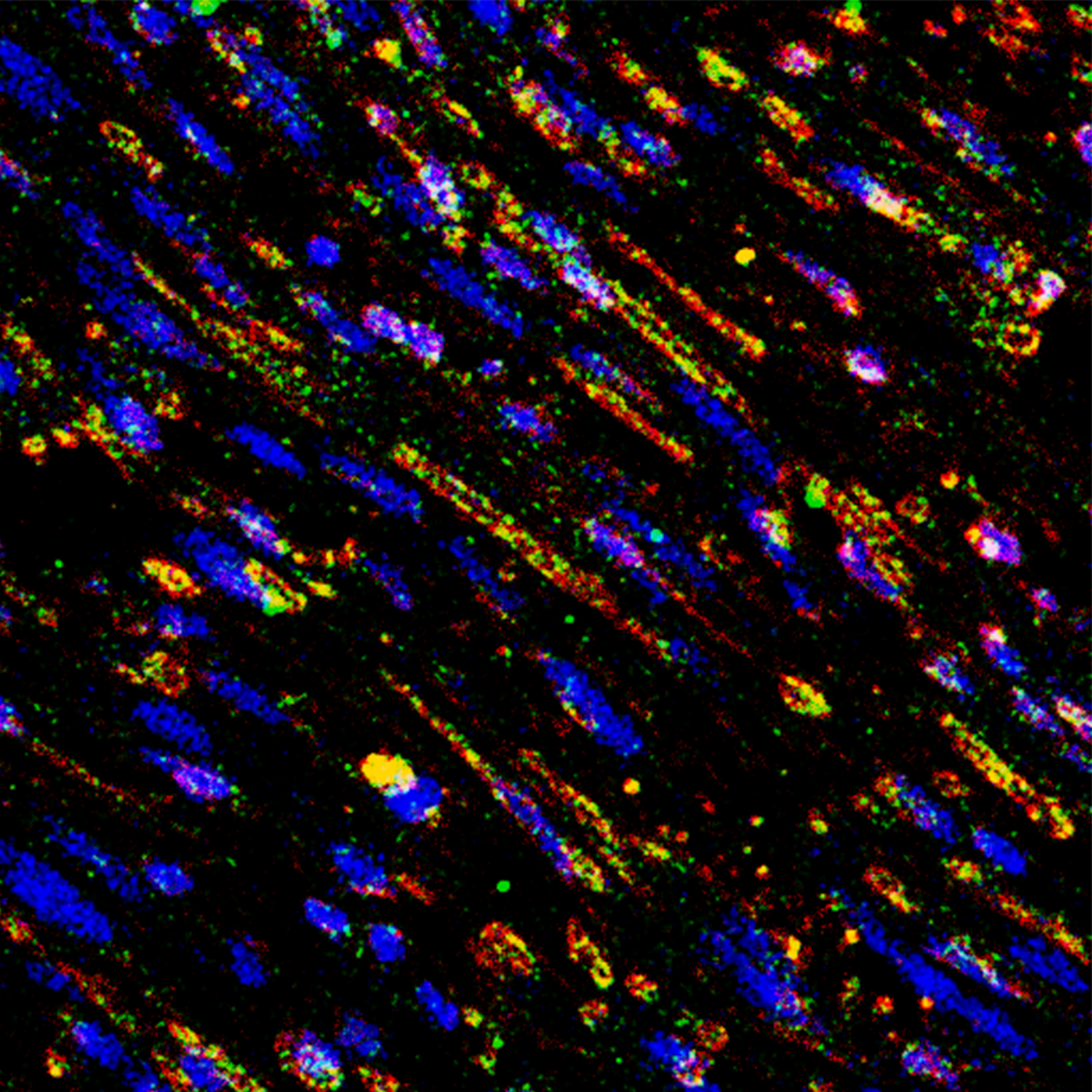

Supplement: Supplementary file 8 [file DataSheet5.ZIP › figure 2C bfgf (3).tiff]

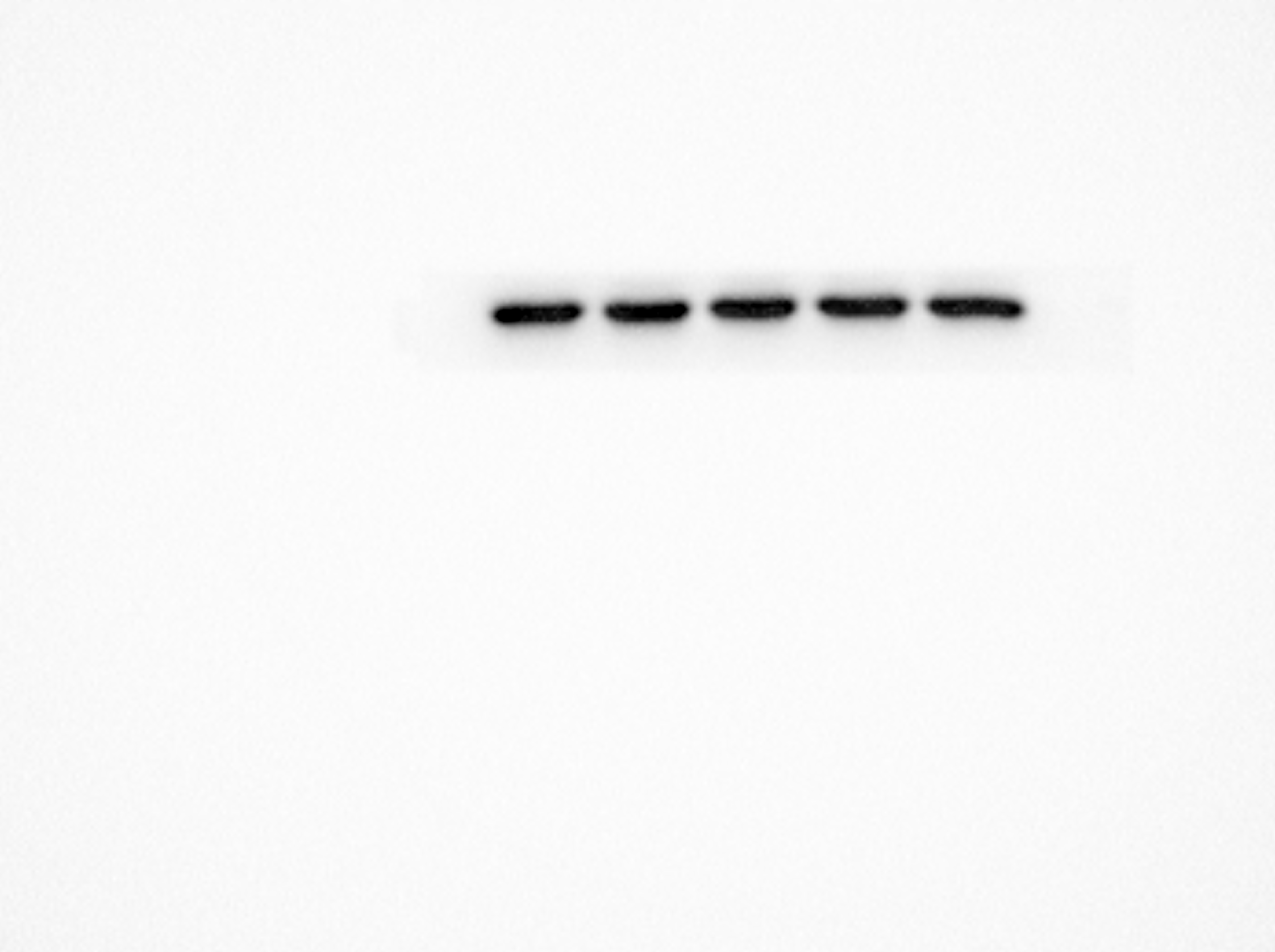

Supplement: Supplementary file 9 [file DataSheet7.ZIP › figure4/figure 4 GADPH.tif]

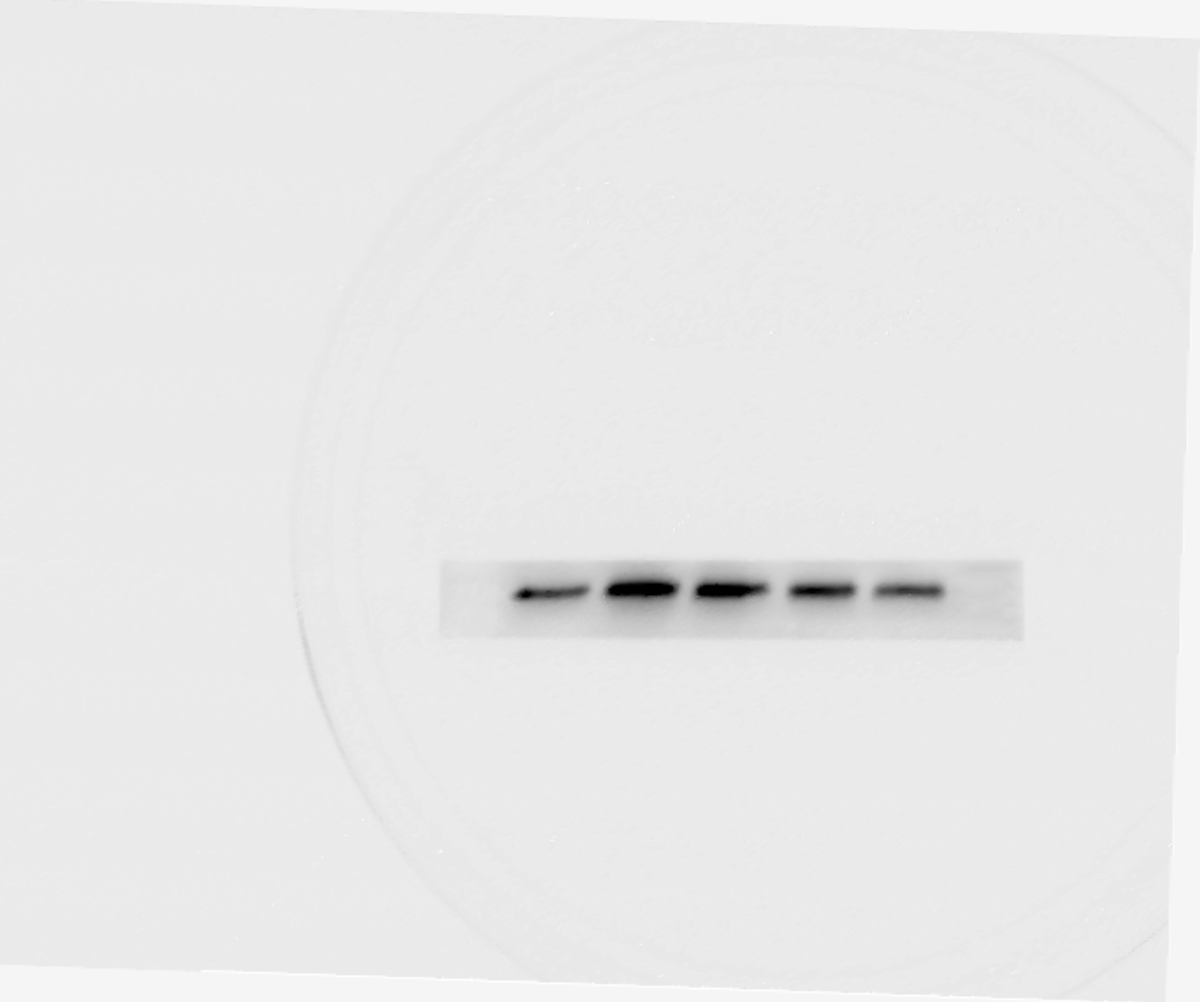

Supplement: Supplementary file 9 [file DataSheet7.ZIP › figure4/figure 4 bax.png]

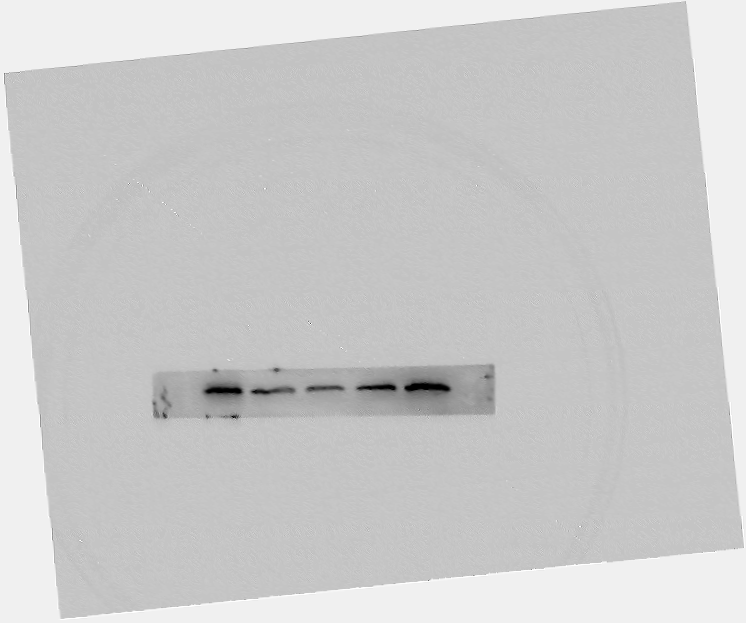

Supplement: Supplementary file 9 [file DataSheet7.ZIP › figure4/figure 4 bcl2 .tif]

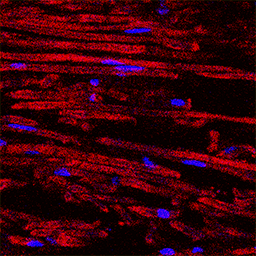

Supplement: Supplementary file 9 [file DataSheet7.ZIP › figure4/figure 4A FNI (1).tif]

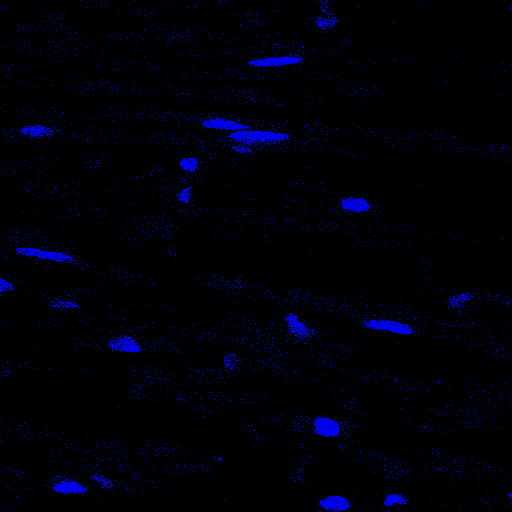

Supplement: Supplementary file 9 [file DataSheet7.ZIP › figure4/figure 4A FNI (2).tif]

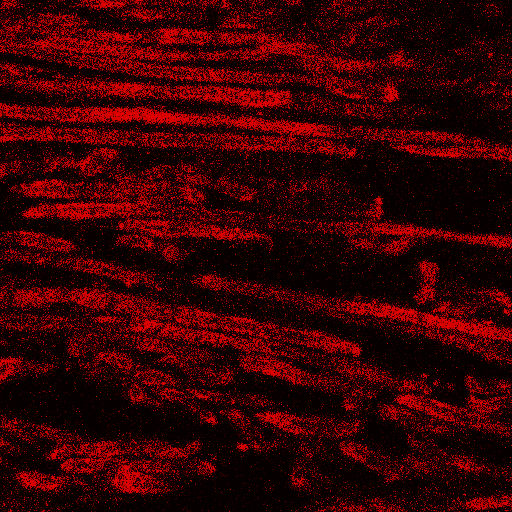

Supplement: Supplementary file 9 [file DataSheet7.ZIP › figure4/figure 4A FNI (3).tif]
