# Supplementary figures and images for: Delivery of Basic Fibroblast Growth Factor Through an In Situ Forming Smart Hydrogel Activates Autophagy in Schwann Cells and Improves Facial Nerves Generation via the PAK-1 Signaling Pathway (part 2 of 2)
Source: Front Pharmacol. 2022 Apr 1;13:778680. doi: 10.3389/fphar.2022.778680 (PMC9011134; doi:10.3389/fphar.2022.778680)

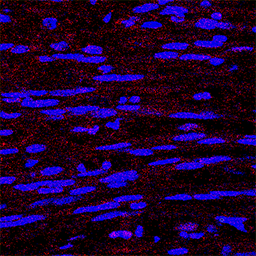

Supplement: Supplementary file 9 [file DataSheet7.ZIP › figure4/figure 4A P-bFGF (1).tif]

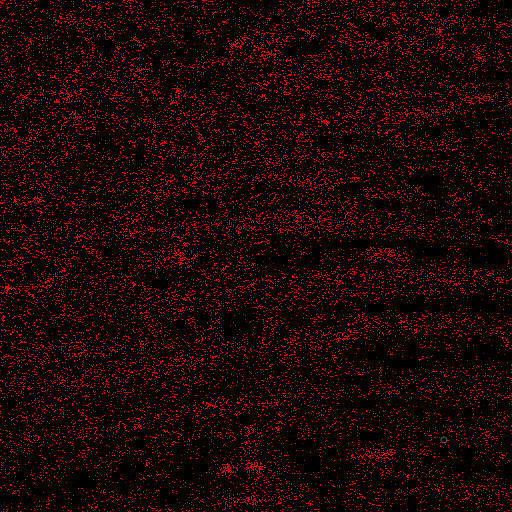

Supplement: Supplementary file 9 [file DataSheet7.ZIP › figure4/figure 4A P-bFGF (2).tif]

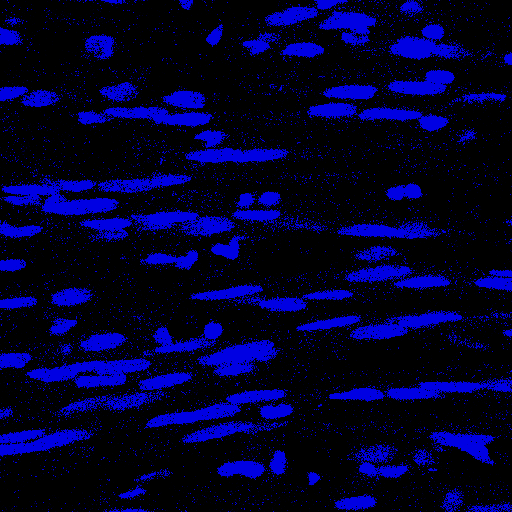

Supplement: Supplementary file 9 [file DataSheet7.ZIP › figure4/figure 4A P-bFGF (3).tif]

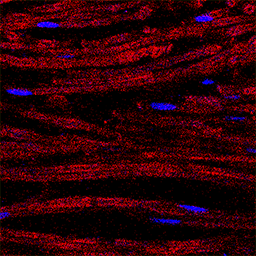

Supplement: Supplementary file 9 [file DataSheet7.ZIP › figure4/figure 4A Poloxamer (1).tif]

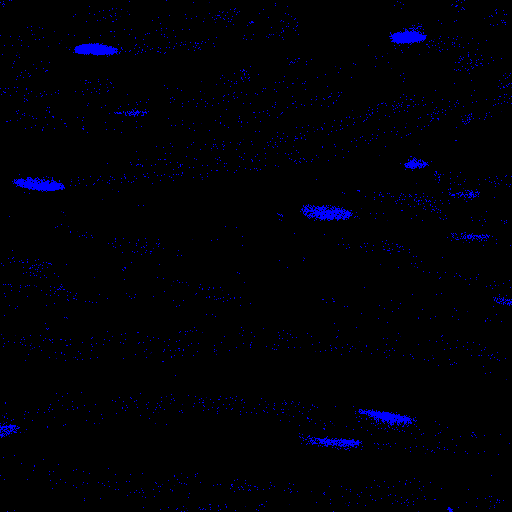

Supplement: Supplementary file 9 [file DataSheet7.ZIP › figure4/figure 4A Poloxamer (2).tif]

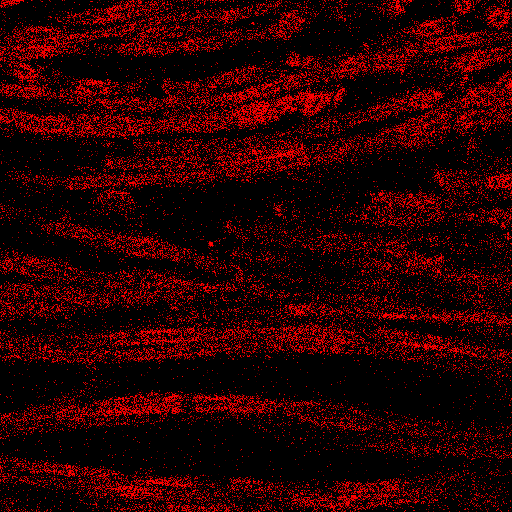

Supplement: Supplementary file 9 [file DataSheet7.ZIP › figure4/figure 4A Poloxamer (3).tif]

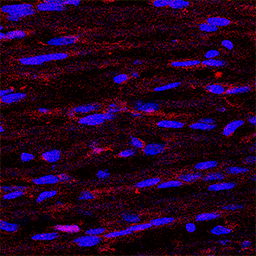

Supplement: Supplementary file 9 [file DataSheet7.ZIP › figure4/figure 4A bFGF (1).tif]

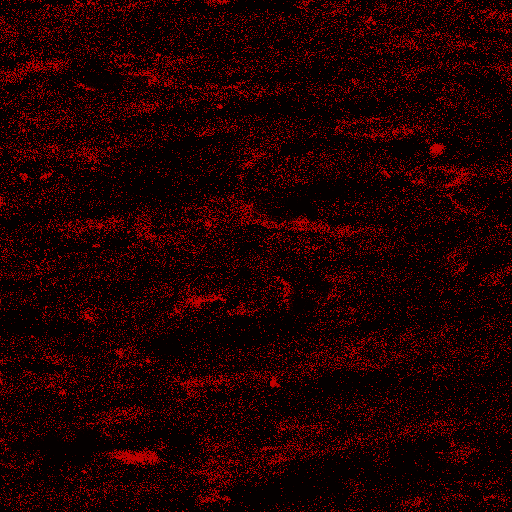

Supplement: Supplementary file 9 [file DataSheet7.ZIP › figure4/figure 4A bFGF (2).tif]

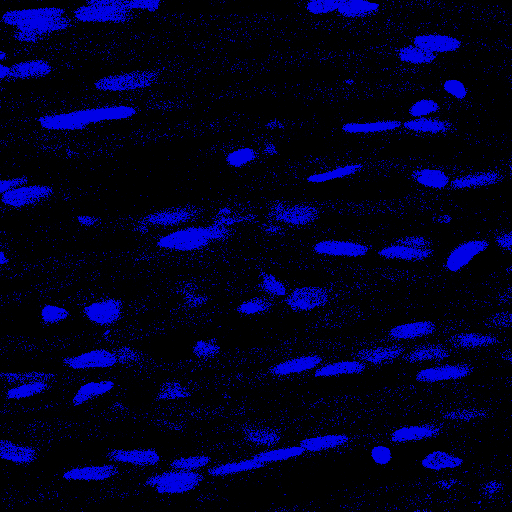

Supplement: Supplementary file 9 [file DataSheet7.ZIP › figure4/figure 4A bFGF (3).tif]

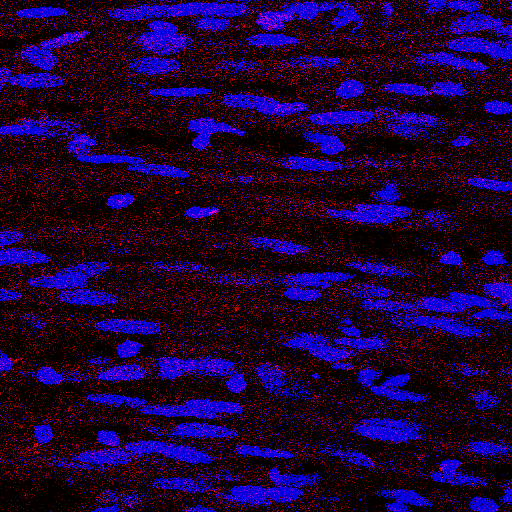

Supplement: Supplementary file 9 [file DataSheet7.ZIP › figure4/figure 4A sham (1).tif]

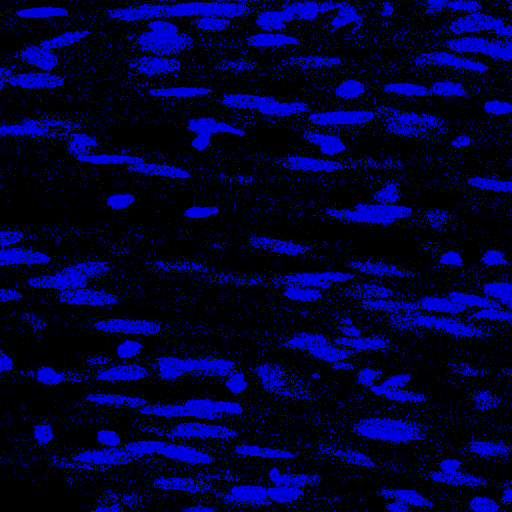

Supplement: Supplementary file 9 [file DataSheet7.ZIP › figure4/figure 4A sham (2).tif]

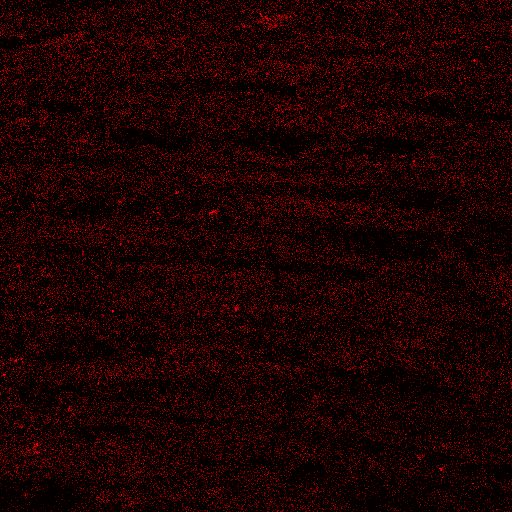

Supplement: Supplementary file 9 [file DataSheet7.ZIP › figure4/figure 4A sham (3).tif]
